# Unexpected *E*-to-*Z* Isomerizations during the Negishi-Type Homocoupling of *E-*Iodoalkenes

**DOI:** 10.1021/acs.joc.3c02957

**Published:** 2024-09-17

**Authors:** Fernanda A. Crovara, Josep Martí, Anna M. Costa, Jaume Vilarrasa

**Affiliations:** Organic Chemistry Section, Facultat de Química, Universitat de Barcelona, Diagonal 645, 08028 Barcelona, Catalonia, Spain

## Abstract

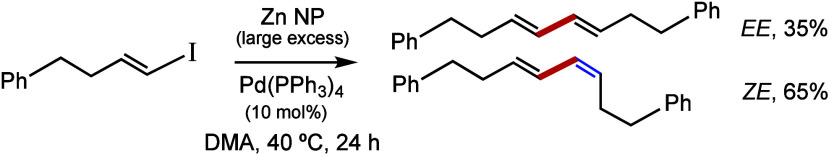

The direct insertion of Zn into olefin–halide bonds is a challenge. When (*E*)-alkenyl iodides were treated with a very large excess of Zn nanoparticles, in the presence of Pd(PPh_3_)_4_, the dimerization was observed but, unexpectedly, yielding mainly *Z,E*-1,3-dienes. This apparently contrathermodynamic *E*-to-*Z* isomerization of organometallic intermediates is predicted to be general and is explained with the aid of DFT [principally M06/6-311+G(d,p)], MP2, and CCSD(T) calculations.

In the past 25 years our research group has been involved in the synthesis, bioevaluation, and molecular docking studies of several cytotoxic macrolides.^1^ Often, the presence of various conjugated dienes in their structures has posed the problem of how to control the stereoselectivity of the formation of the second double bond by C(sp^2^)–C(sp^2^) coupling reactions.^2^ When this coupling is planned to be carried out with advanced fragments/synthons/chiroblocks in a multistep synthesis, all the methods have pros and cons. The Pd-catalyzed Negishi reaction has advantages when alkenylzinc halides to be coupled (R*CH=CH–ZnX) contain various functional groups and prone-to-inversion stereocenters (in R*). However, as is known,^3^ the direct zincation of haloalkenes (nonactivated by electron-withdrawing groups (EWGs)) is particularly complicated; that is, it is more difficult to insert Zn into olefin–halide bonds of nonactivated alkenes than into most other C–X bonds.^4^ It is common to resort to lithiation (with ≥2 equiv of ^*t*^BuLi) or magnesiation, followed by in situ Li-to-Zn or Mg-to-Zn exchange with ZnX_2_, but it may be incompatible with the functional and protecting groups of R*. The question is how to carry out direct Zn insertion into a vinyl iodide.

In preliminary experiments, before attempting cross-couplings with expensive advanced fragments, we prepared simple iodovinyl derivatives as substrates (RCH=CHI) and examined their dimerization with a simple and well-known “activator” of C(sp^2^)–X bonds, Pd^0^. In our hands, with Zn and Pd, the expected conversion of (*E*)-1-iodo-4-phenyl-1-butene (**1**) to (*E,E*)-**2** (Scheme 1), henceforward also *EE-***2**, occurred in 76% yield (not optimized). There are, obviously, many precedents of homocouplings of alkenylmetal derivatives (prepared from vinyl iodides),^5^ but we wanted to focus on Zn-mediated Negishi-type reactions.

**Scheme 1:**
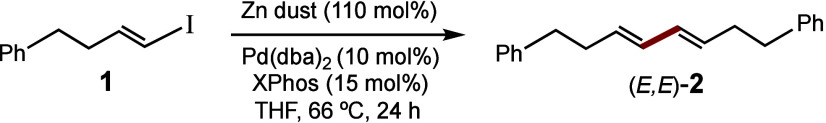
Dimerization of Alkenyl Iodide 1

To our surprise, in some experiments with activated Zn dust a byproduct was detected, which under appropriate conditions and a large excess of Zn nanopowder (NP) turned out to be the major compound in the final mixture. This product was the *ZE* diene. Sometimes, during the reaction of metalated alkenes, a partial inversion of configuration of the double bond has been reported,^6^ but the *E*-to-*Z* isomerization detected here is unprecedented, to the best of our knowledge (searching with SciFinder^n^).^7^ We therefore investigated the self-coupling of (*E*)-alkenyl iodides to afford *ZE* dimers. This is the subject of the present Note.

To pure **1** in *N,N*-dimethylacetamide (DMA) a large excess of Zn NP (up to 500 mol %) was added, and afterward Pd(PPh_3_)_4_ (10 mol %). The mixture was shaken or vigorously stirred at 40 °C, under Ar, overnight or for 24 h. After dilution with hexane(s), filtering the excess metal, and washing with dilute acid, unexpected *ZE*-**2** was the major compound. The crude mixture was not separated but was analyzed by NMR and GC-MS. The symmetry of the *EE* isomer allowed us to distinguish it from its *ZE* isomer by ^1^H NMR spectroscopy (Figure 1). Reference samples of pure *EE*-**2** and *ZE*-**2** were prepared by standard reactions, that is, from **1** + 2 ^*t*^BuLi + ZnBr_2_ in THF, addition of Pd(PPh_3_)_4_, and of a second equiv of **1** or *Z-***1**, respectively.

**Figure 1:**
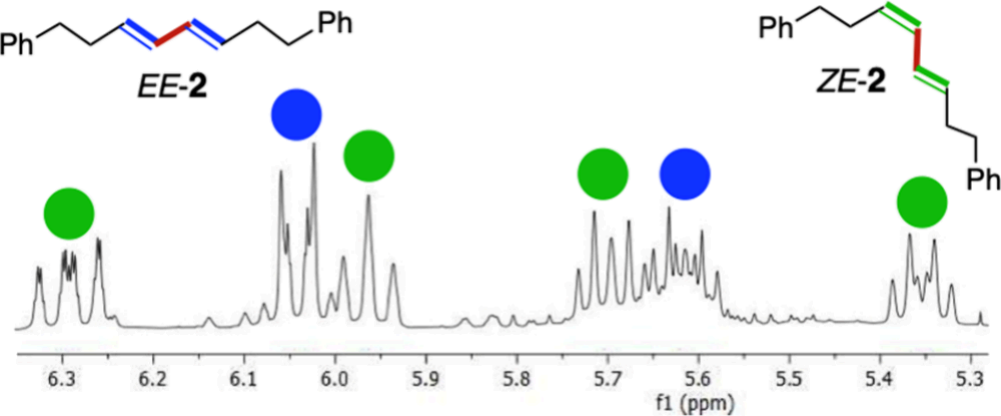
^1^H NMR spectrum of the olefinic region of the crude product (mixture of *EE-***2** and *ZE*-**2**) obtained from **1**/Zn NP/Pd^0^.

Other reaction conditions and substrates were examined. The results are summarized in Table 1 and the following paragraphs.

**Table 1:**
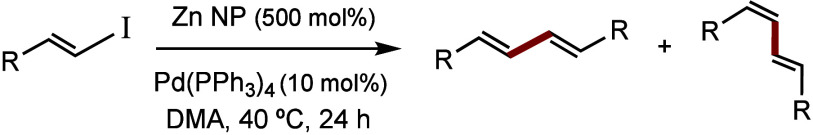

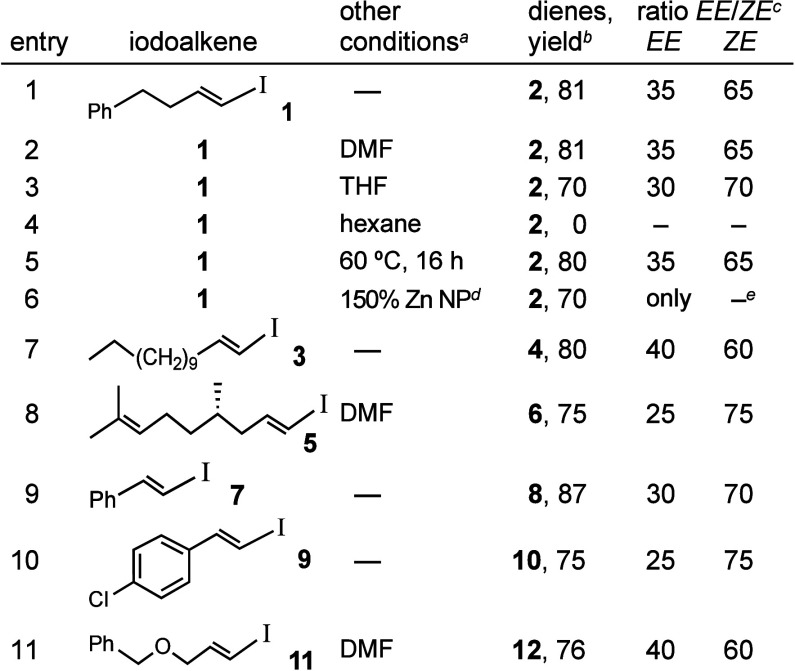
Dimerization of Iodovinyl Derivatives

a Variations from the standard conditions.

b Yields of the mixtures.

c These ratios are mean values from different trials and from ^1^H NMR and GC or HPLC. Small percentages (2–9%) of suspected-to-be dienes *ZZ* were often detected, see the Supporting Information. With the *Z* isomer of **1** (not included in Table 1 for the sake of simplicity) we obtained a mixture of *ZZ/ZE/EE* dimers; the possible partial stereoinversion of *Z* vinylmetal intermediates, although less surprising,^8^ would deserve to be studied independently.

d With 250 mol %, the *EE/ZE* ratio was nearly 1:1; with 1000 mol %, the ratio was the same as with 500 mol %.

e Not detected.

Table 1 shows that similar results were obtained: (i) with DMA, DMF, and THF; (ii) by increasing the temperature to 60 °C; and (iii) with other vinyl iodides, linked to either aliphatic chains or aromatic rings. The large excess of reducing agent, which may shorten the lifetime of Pd(II) species, thus relatively slowing the homocoupling step, is crucial.

Also with 500 mol % of Zn NP, the addition of 10 mol % of either Pd(dba)_2_/Xantphos, Pd(dba)_2_/XPhos, or Pd(OAc)_2_/2PPh_3_ yielded lower percentages of *ZE*-**2** than of *EE*-**2**. In short, although *EE*/*ZE* ratios were around 1:2 as a mean value with Pd(PPh_3_)_4_, they were around 2:1 with other Pd sources and ligands. Thus, a Pd(0) source less reactive or more amenable to undergo a rapid Pd(II) to Pd(0) reduction is instrumental. We believe that the surprising formation of *ZE* dimers from *E*-vinyl iodides has not been reported previously because Zn NP is seldom used in Negishi reactions. Moreover, it made no sense to add such an excess of Zn; in our trial experiments, we did so merely to accelerate the reduction of RCH=CH–PdL_2_X to Pd^0^, with the intention of filtering the large excess of Zn when the zincation reaction was completed.

The stereoinversion did not occur at the end of the reaction. As expected, we did not observe a partial conversion of *EE* dienes into *ZE* dienes under the reaction conditions,^9^ that is, in the presence of Pd^0^, Pd^II^, PPh_3_, Zn, or combinations of them.

Organometallic compounds of *Z* configuration must thus be formed in one or another intermediate step of the process, whatever the reaction mechanism (ionic or radical). As shown in Table 2, first four rows, we compared the relative stability of *E* and *Z* isomers of vinylzinc halides with the M06/6-311+G(d,p) method, which is recommended for organometallic compounds.^10^ For confirmation, we often applied other DFT methods, as well as MP2 and CCSD(T);^11^ the LANL2DZ basis set was used for elements > Kr.^12^

**Table 2:**
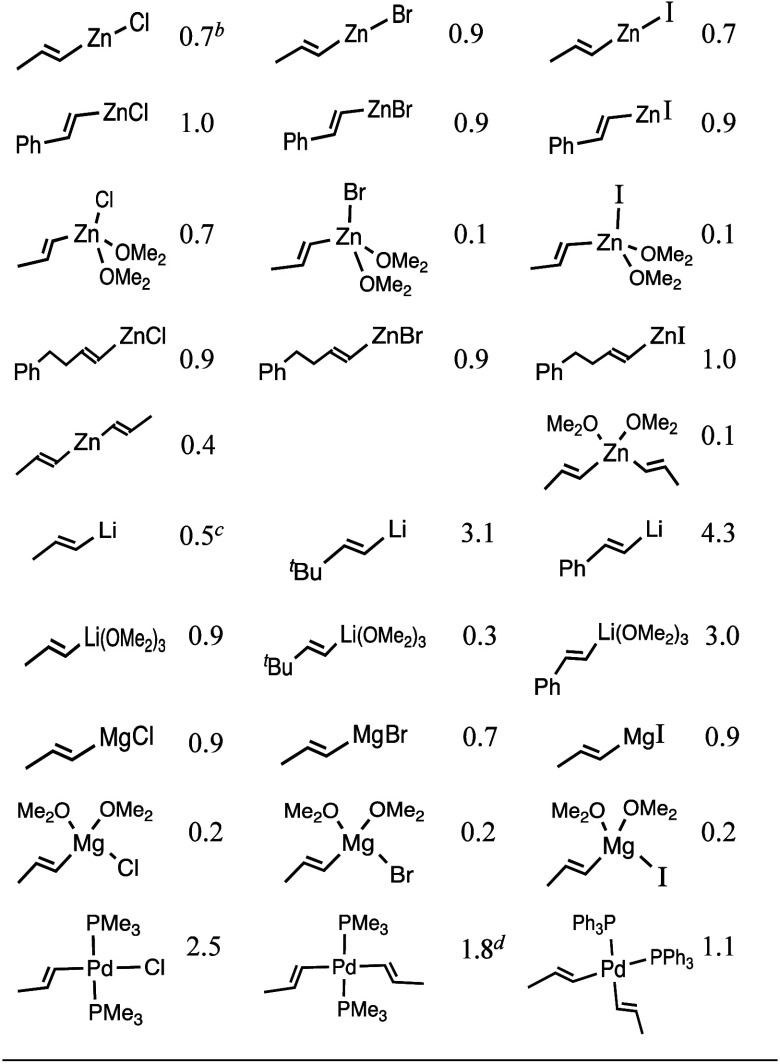
Relative Energies, in kcal/mol, of *E* vs. *Z* Alkenylmetal Halides and of *EE* vs. *ZE* Dialkenylmetal Compounds^a^

a From M06/6-311+G(d,p) energies. Other DFT methods, MP2/6-311+G(d,p), and CCSD(T)/6-311+G(d,p) gave similar gaps in most cases (see Supporting Information).

b 0.7 in vacuum, 0.7 in THF/CPCM, and 0.6 in DMF/CPCM.

c 0.4 in THF/CPCM and 0.4 in DMF/CPCM.

d For analogous Ni compounds, see also the Supporting Information (Table S1).

To our initial surprise, the *Z*-alkenyl intermediates were predicted to be favored with respect to the respective *E*-alkenyl intermediates. In fact, Table 2 shows that (*E*)-MeCH=CHZnX and (*E*)-MeCH=CHZn(OMe_2_)_2_X are generally less stable than the corresponding *Z* isomers. This also occurs with PhCH=CHZnX and other RCH=CHZnX, such as derivatives of **1** (PhCH_2_CH_2_CH=CHZnX). The gaps are smaller at the CCSD(T) level (Supporting Information), but a *cis* effect (*Z* effect) is evident. In organic chemistry the classical *cis* effect refers to the 1,2-disubstituted double bonds in which isomer *Z* is more stable than isomer *E* (*cis* effect in olefins, cEO); in inorganic chemistry the concept is used to explain the *cis-*destabilizing effect of some ligands in octahedral coordination complexes (cECC).

To summarize, the final *ZE* dienes are not thermodynamically favored, as expected, but the *Z*-alkenylzinc halides are. As these *Z* intermediates have lower energies than or similar energies as the respective *E* species, the products that arise from the former intermediates may be considered to be (slightly) kinetically favored.

Furthermore, we calculated the total energies of the *E* and *Z* isomers of other alkenylmetal halides and dialkenyl metals (also see Table 2). M06/6-311+G(d,p)·LANL2DZ(Pd)//M06/6-31G(d)·LANL2DZ(Pd) and M06/6-311+G(d,p)·SDD(Pd)//M06/6-31G(d)·SDD(Pd)^12^ values were also compared.^13^ The effect of solvents and of the entropy and thermal corrections (calculation of *G°*) were also evaluated in several cases, but in general they did not significantly change the outcome of the comparison of the total energies (cf. the Supporting Information). For the complexes, besides THF, we used Me_2_O as a surrogate of Et_2_O and sometimes Me_3_P instead of Ph_3_P.

The results were in agreement: alkenylmetal halides of the *Z* configuration are thermodynamically favored. In other words, Table 2 indicates that the above-mentioned *cis* effect is general. One explanation may be based on favorable intramolecular interactions (vdW forces, noncovalent interactions). A related explanation is that the polarization of the C–M bond favors the species with the *cis* Me group (or R or Ar groups), from an electrostatic point of view, in the same way as the 1-propenyl anion with the negative charge *cis* to Me is thermodynamically more stable than its *trans* anion,^14^ as shown in Figure 2. For PhCH=CH^–^ there is a difference of ≥2.0 kcal/mol in favor of the species with the negative charge *cis* to Ph. All these values are in the gas phase; in THF and in DMF the predicted gaps are smaller (0.8–1.0 kcal/mol). B3LYP-D3 calculations^15^ with Pople, Dunning, or Ahlrichs basis sets gave similar results to those indicated in Figure 2.

**Figure 2:**
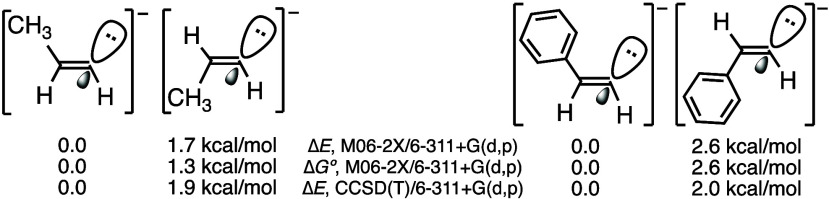
Relative energies of the two stereoisomers of the 1-propenyl anion and of the two isomers of the 2-phenylethenyl anion.

Proposals and hypotheses for *Z*-to-*E* isomerizations have been published.^2a,5a,7^ To complement these proposals and to try to understand the present *E*-to-*Z* case, further mechanisms may be considered. For example, the coordination of Pd(0) to (*E*)-alkenylzinc iodides (Scheme 2) might facilitate the *E*/*Z* equilibrium as the C=C bond order may decrease. The Pd^0^/Pd^II^ ratio may be relatively high throughout due to the large excess of the reducing agent (Zn NP) in the medium. It can thus be assumed that some (*E*)-RCH=CH–PdIL_2_ is converted into (*E*)-RCH=CH–ZnI, which in part reacts with the remaining (*E*)-RCH=CH–PdIL_2_ and in part equilibrates with its *Z* isomer (by stereoinversion at C1 or at C2); this isomer also reacts with (*E*)-RCH=CH–PdIL_2_, as suggested in Scheme 2. For the sake of simplicity, we depict the active species as PdL_2_ rather than as PdL_n_, that is, rather than a PdL_3_/PdL_2_/PdL equilibrium (from the probably most abundant but least reactive Pd complex to the least abundant but much more reactive species), in a ratio depending on the features of the ligands.

**Scheme 2:**
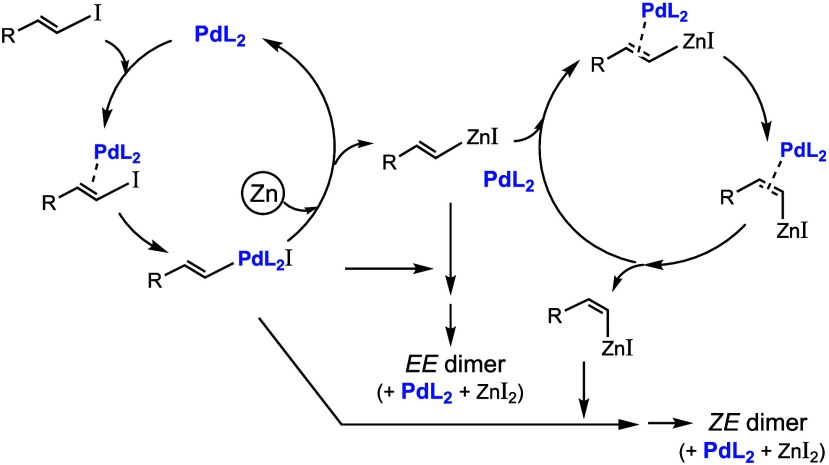
Pd-Catalyzed Dimerization of Alkenyl Halides with a Possible *E*-to-*Z* Isomerization Step of Alkenylzinc Halides

We also speculated that the configuration inversion may occur through aggregates by migration insertion.^2a,5a^ Another possibility is that it takes place during the formation of Pd(CH=CHMe)_2_L_2_ species^8^ or by equilibration of these Pd(II) complexes. DFT calculations^16^ (see Scheme 3) suggest that the *ZE* isomers of these complexes may have lower energies than the respective *EE* isomers. Thus, partial isomerization to *ZE* complexes is feasible.

**Scheme 3:**
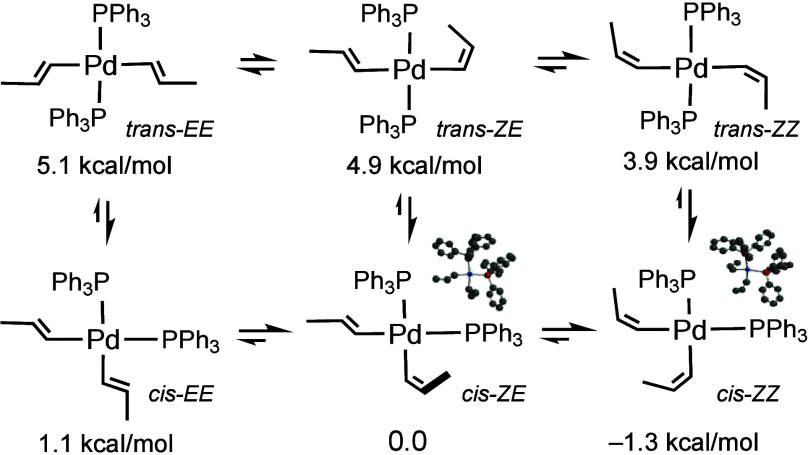
Calculated Relative Energies of Pd–Dialkenyl Intermediates From M06/6-311+G(d,p)//M06/6-31G(d) energies, with the LANL2DZ basis set for Pd.

Independently, it is worth noting that the M06 method predicts that the *cis*-dialkenylpalladium intermediates shown in Scheme 3 are favored with regard to the respective *trans*-isomers. The short lifetime of these *cis-EE* and *cis-ZE* intermediates, when formed, might be the cause of the limited isomerization of *ZE* to the even more stable *ZZ* intermediates, given that the *ZZ*-dienes have only been observed in very small percentages (<9%, see footnote c in Table 1 and the Supporting Information).

In conclusion, by means of the Negishi organozinc chemistry, it is possible to dimerize *E-*vinyl iodides with stereoretention (*EE*-dienes, Scheme 1), as expected, but under appropriate conditions *ZE* dienes are the major products (Table 1). The *Z* effect (classical *cis* effect in olefins, cEO) explains that *E-*to-*Z* isomerizations of alkenylmetal intermediates are thermodynamically feasible and general, according to DFT, MP2, and CCSD(T) calculations (Tables 2 and S1). We look forward to gaining more insight into the mechanism(s) of these isomerizations, optimizing the reaction conditions for the practical preparation of pure *ZE*-1,3-dienes, and carrying out alkenyl–alkenyl cross-couplings with multifunctional substrates sensitive to the previous lithiation or magnesiation.

## Data Availability

The data underlying this study are available in the published article and its Supporting Information.

## Supporting Information Available

Experimental section (general methods, preparation and characterization of **1**–**12**), calculation of energies of *E* and *Z* alkenylmetal halides and of *EE*/*ZE*/*ZZ* dialkenylmetal intermediates, M06-2X, B3LYP-D3, MP2, and CCSD(T) energies of alkenide ions, calculation of plausible Schlenk-type equilibria, calculated energies of dialkenylpalladium(0) reaction intermediates, equilibrium geometries, and references (PDF)
