# Supplementary material for: Unexpected E-to-Z Isomerizations during the Negishi-Type Homocoupling of E-Iodoalkenes
Source: J Org Chem. 2024 Sep 17;89(19):14483–8. doi: 10.1021/acs.joc.3c02957 (PMC11580178; doi:10.1021/acs.joc.3c02957)
Supplement: Supplementary file 1 — jo3c02957_si_001.pdf [file jo3c02957_si_001.pdf]

Supporting Information

Unexpected *E*-to-*Z* Isomerizations during the Negishi-Type Homocoupling of *E*-Iodoalkenes

Fernanda A. Crovara, Josep Martí, Anna M. Costa,* and Jaume Vilarrasa*

Organic Chemistry Section, Facultat de Química, Universitat de Barcelona, Diagonal 645, 08028 Barcelona, Catalonia, Spain

amcosta@ub.edu, jvilarrasa@ub.edu

Experimental Section
General Methods S2
Preparation and Characterization of **1–12** S2
Calculation of Energies of *E* and *Z* Alkenylmetal Halides and of *EE*/*ZE*/*ZZ* Dialkenylmetal Intermediates S14
M06-2X, B3LYP-D3, MP2, and CCSD(T) Energies of Alkenide Ions (Alkenyl Anions) S16
Calculation of Plausible Schlenk-Type Equilibria S17
Calculated Energies of Dialkenylpalladium(0) Reaction Intermediates S17
Equilibrium Geometries (Cartesian Coordinates) S19
References S32

Experimental Section

General Methods

Unless otherwise noted, all reagents were purchased from commercial suppliers and used without further purification. Zn nanopowder (Zn NP, 40–60 nm average particle size) was purchased from Merck/Sigma–Aldrich. All reactions were conducted under an inert argon atmosphere and using anhydrous solvents. Solvents were obtained from commercial sources, dried, and purified, when necessary, according to standard procedures; when used in purification processes (by extraction and column chromato-graphy), they were only distilled. Thin layer chromatography (TLC) was carried out on analytical plates with silica gel (F254 Merck) of 0.2 nm thickness and analyzed with UV light (254 nm) and then stained with a basic solution of $KMnO_4$ or an acidic solution of *p*-anisaldehyde. Column chromatography was carried out under low pressure (flash) conditions and performed on silica gel 60 (35–70 µm). Preparative TLC on alumina was employed in some cases to purify very small quantities of compounds. Gas chromatography/mass spectrometry (GC-MS) experiments were performed using an HP 6890 Series, GC system, and an HP 5973 Mass Selective Detector, with a HP-5MS column (5% phenyl methyl siloxane, 30 m x 250 µm x 0.25 µm), a temperature gradient of 5 ºC/min, from 100 ºC to 300 ºC, and a N_2 flow of 0.8 mL/min. High-performance liquid chromatography (HPLC) was carried out using a Shimadzu LC-6A pump under isocratic conditions, a manual Rheodyne in-jector (with a 20 µL loop), a Shimadzu UV detector (254 nm), and a Shimadzu C-R6A integrator-recorder; a Chiralpak IA column with a flow rate of 0.5 mL/min was used. The mobile phase and retention times are specified in each case. The solvent used in ^{1}H NMR (400 MHz) and ^{13}C NMR (100.6 MHz) experiments was $CDCl_3$ in all cases. The spectra were referenced to internal TMS (δ 0.00 for ^{1}H NMR) and $CDCl_3$ (δ 77.0 for ^{13}C NMR), with chemical shifts given in units of δ (ppm).

Preparation and Characterization of 1–12

Vinyl iodides **1**, **3**, **5**, **7**, **9**, and **11** were prepared by us from the corresponding aldehydes by iodomethylenation with CHI_3 and $CrCl_2$ according to the procedure of Takai et al.[S1] Iodoalkene **1** was also prepared by treatment of the corresponding alkyne with DIBALH or with $ZrCp_2Cl_2$/DIBALH, followed by iodination.[S2] Compounds **1**,[S3] **2**,[S4] **3**,[S1,S5] **5**,[S6] **7**,[S7] **8**,[S8] **9**,[S9] **10**,[S8,S10] **11**,[S11] and **12**[S12] are known; many of them are commercially available.

Thus, **4** and **6** were unknown; the HRMS of the *ZE+EE* mixtures were registered. NMR spectra and GC-MS of the crude products were recorded to ensure that purification did not alter the ratios. Some NMR spectra of known compounds are also reproduced just to show its purity, the *ZE/EE* ratios, and/or the presence of *ZZ* isomers in trace amounts (or, less frequently, up to 9%).

General procedure for the dimerization of iodoalkenes. Representative example (from 1 to the *EE*-2/*ZE*-2 mixture)
A solution of $Pd(PPh_3)_4$ (46 mg, 0.040 mmol, 10 mol%) in DMA (0.5 mL) was added, via cannula, to a suspension of Zn NP (131 mg, 2.0 mmol, 500 mol%) and vinyl iodide **1** (104 mg, 0.40 mmol) in DMA (0.5 mL) under Ar. After vigorous stirring or shaking for 24 h at 40 ºC (aluminum reaction block), hexane(s) (10 mL) was added, and the suspension was filtered (to remove the excess of Zn). Dilute acid (2 M HCl, 10 mL) was then added, and the phases were separated. The aqueous phase was extracted with hexane (3 x 5 mL). The combined organic phases were washed with acid and with water, dried over Na_2SO_4, filtered, and con-centrated to dryness. The residue (the crude product mixture) was first analyzed by GC-MS (around 40:60 *EE*-**2**/*ZE*-**2** ratio), sometimes also by HPLC (35:65 *EE*-**2**/*ZE*-**2**), and by ^{1}H NMR spectroscopy (see next page). It was then purified by column chromatography or, in other cases, by preparative TLC on alumina, to obtain 43 mg (81%) of mixture, the ^{1}H NMR spectrum of which was registered again (nearly 35:65 *EE*-**2**/*ZE*-**2**) as well as its ^{13}C NMR spectrum. These spectra agree with those reported.[S4]

400 MHz ^{1}H NMR spectrum of 1[S3] in $CDCl_3$

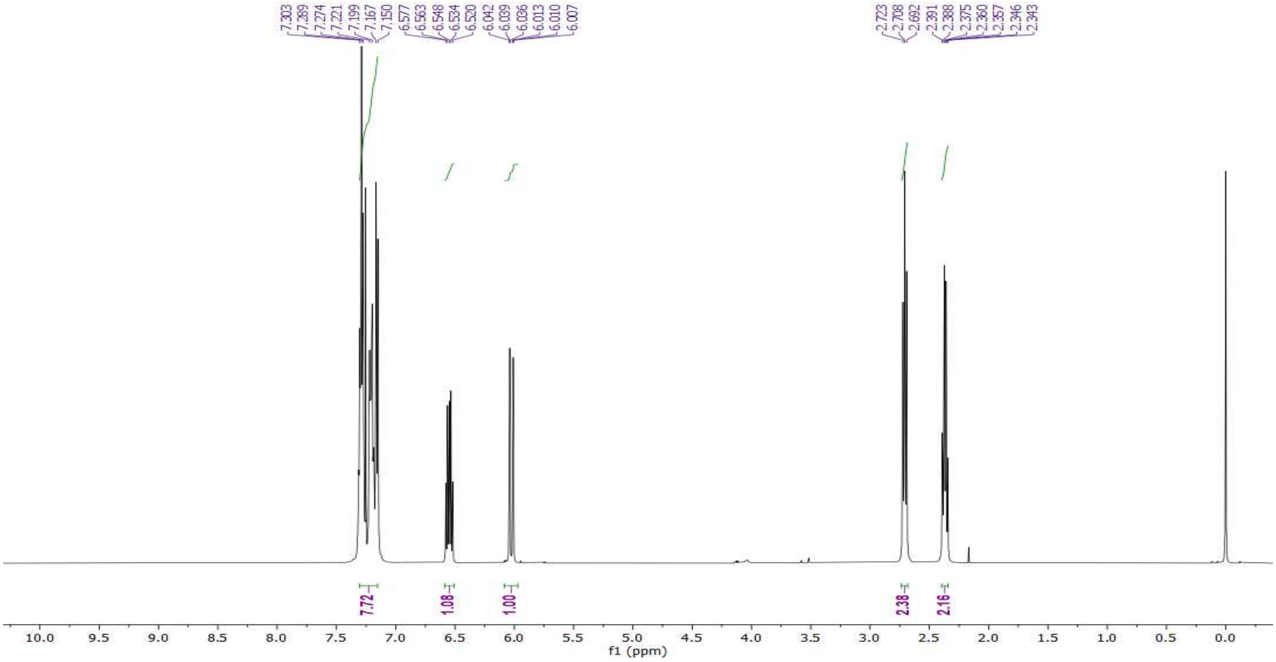

100.6 MHz $^{13}C\{^{1}H\}$ NMR spectrum of 1[S3] in $CDCl_3$

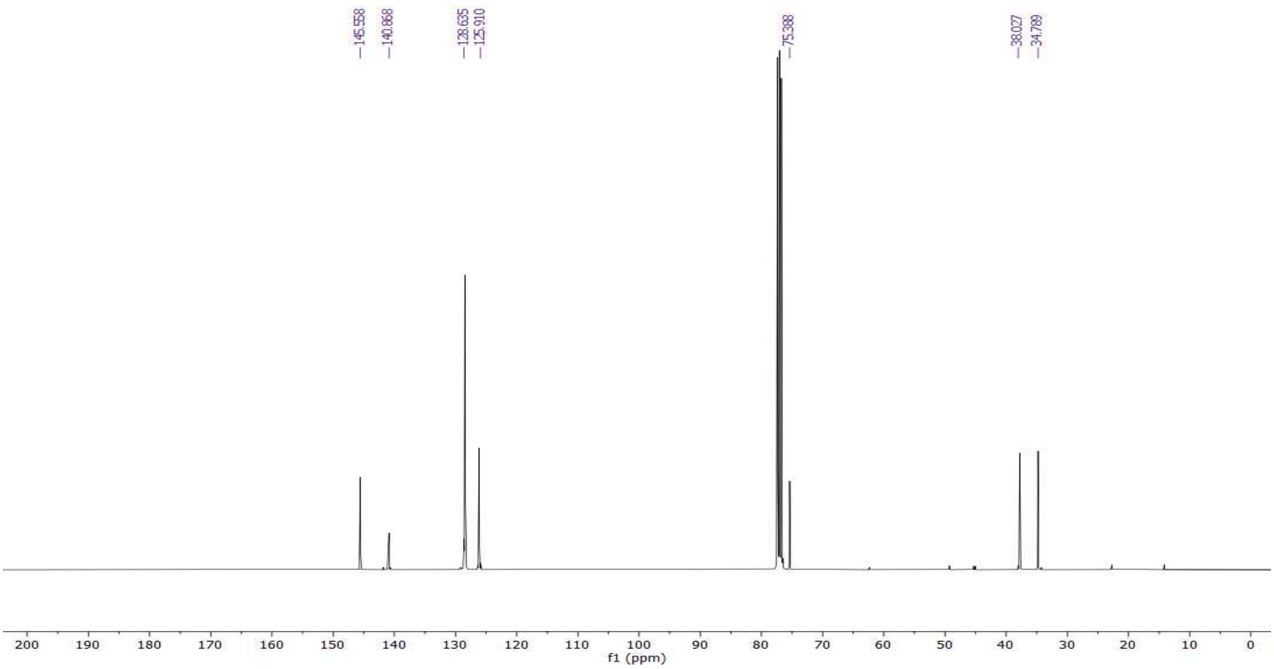

400 MHz ^{1}H NMR spectrum of 2 (crude mixture where *EE*-2 and *ZE*-2 predominated)[S4] in $CDCl_3$

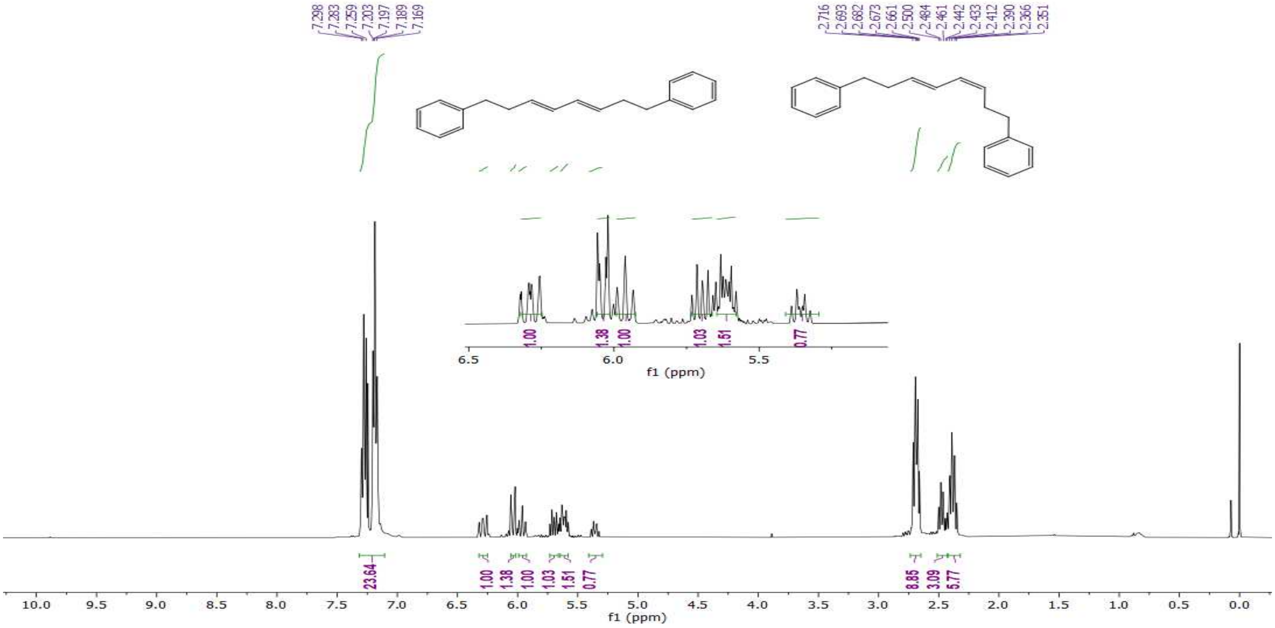

Expansion of the olefinic region of the crude mixture of 2 (400 MHz ^{1}H NMR spectrum in $CDCl_3$)

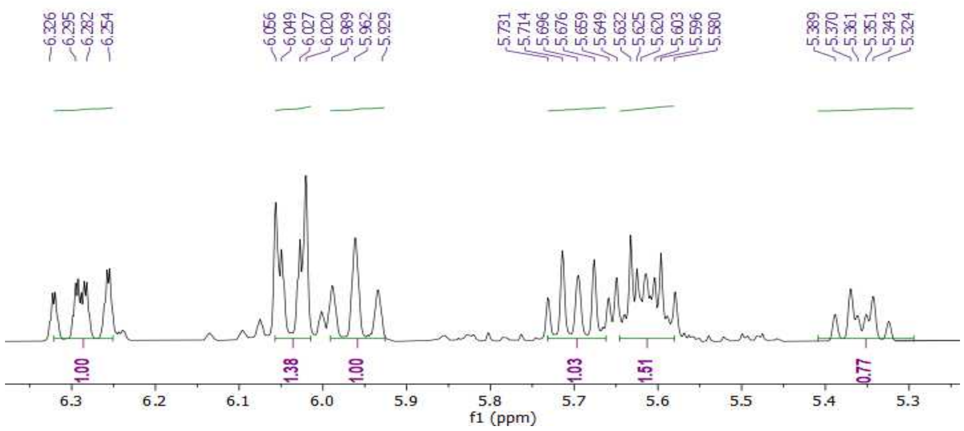

Small signals centered at 6.28 ppm (below the downfield proton of *ZE*-**2**) and around 5.5 ppm might be due to *ZZ*-**2**.
Other impurities have not been characterized.

Reference signals. 400 MHz ^{1}H NMR spectrum (in $CDCl_3$) of the olefinic region of a pure sample of *EE*-2, prepared independently, from 1 + 2 ^{t}BuLi + $ZnBr_2$ followed by the Negishi coupling reaction with 1

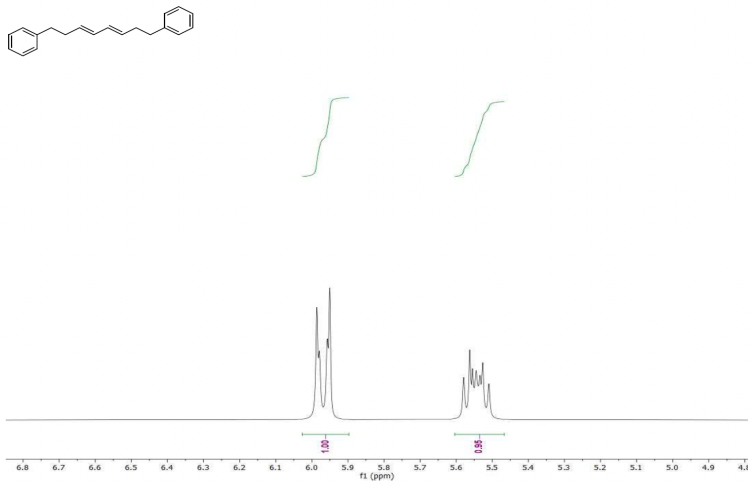

Reference signals. 400 MHz ^{1}H NMR spectrum (in $CDCl_3$) of the olefinic region of a pure sample of *ZE*-2, prepared independently, from 1 + 2 ^{t}BuLi + $ZnBr_2$ followed by the Negishi coupling reaction with *Z*-1

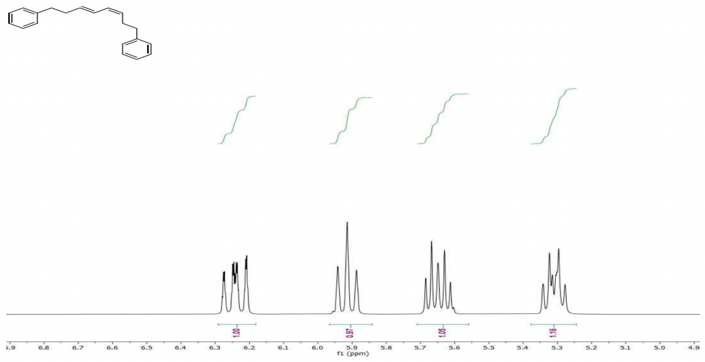

400 MHz ^{1}H NMR spectrum, in $CDCl_3$, of 2 (*EE*-2 + *ZE*-2)[S4] once purified by chromatography on alumina

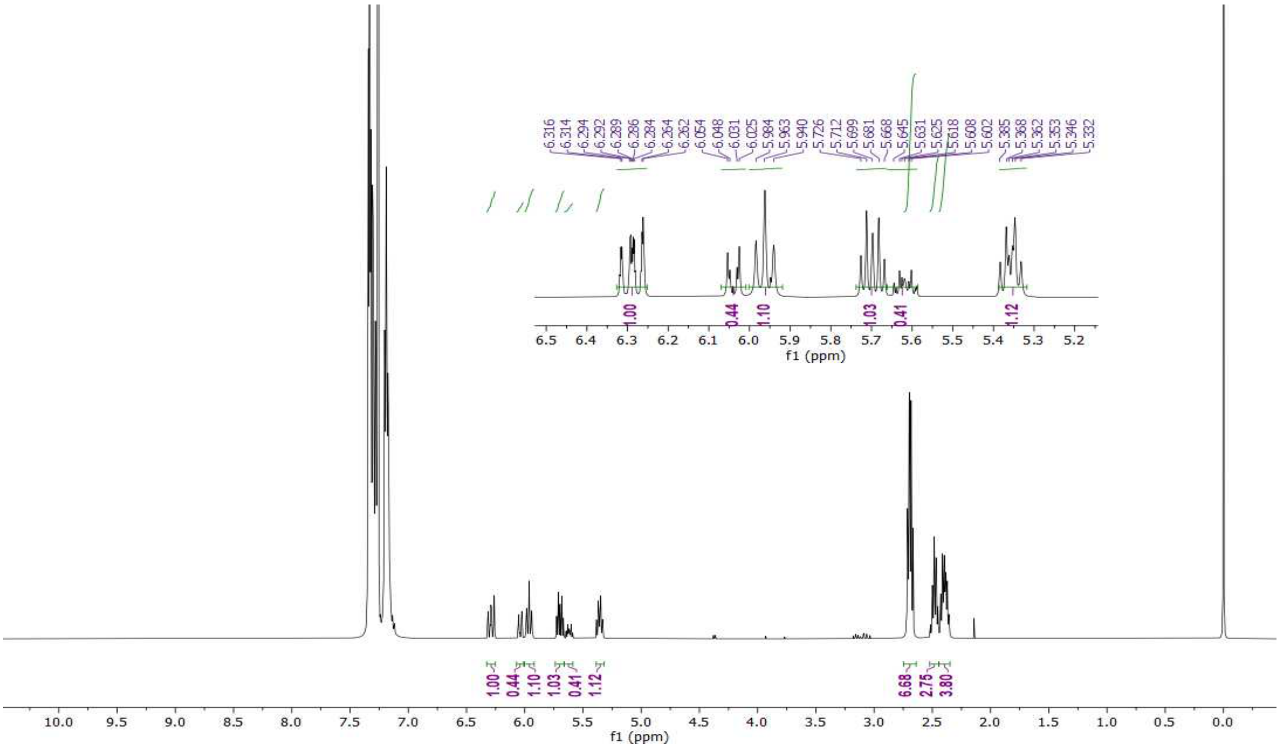

100.6 MHz $^{13}C\{^1H\}$ NMR spectrum of 2 (mixture of *ZE*-2 and *EE*-2)[S4] in $CDCl_3$

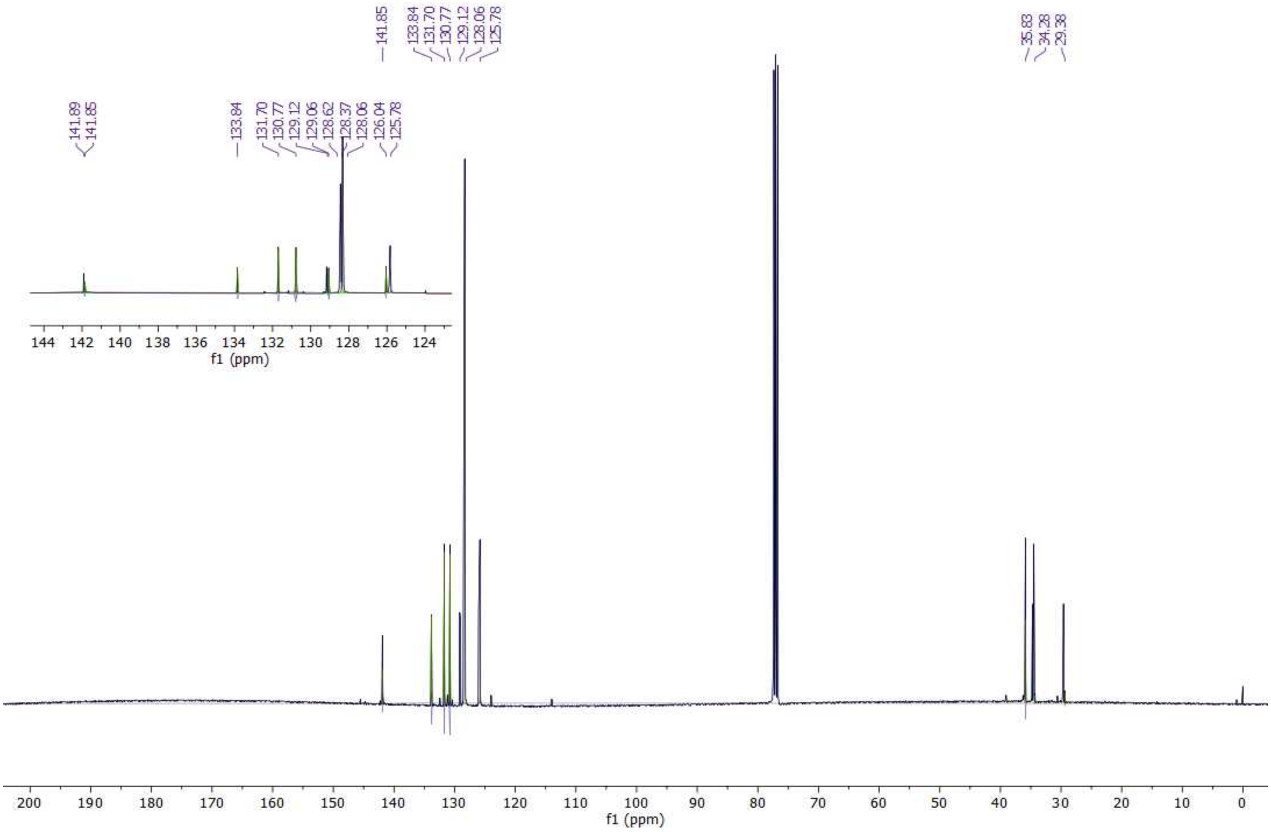

GC-MS of the crude mixture of *ZE*-2 and *EE*-2

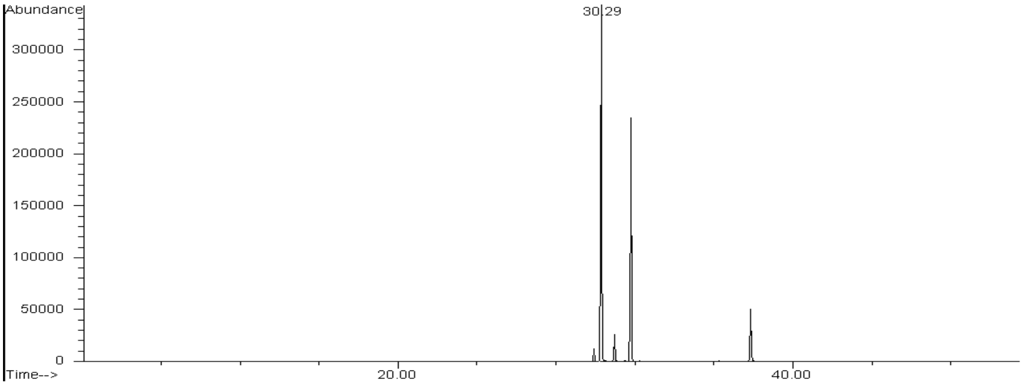

MS of the more intense peak (t_R = 30.3 min)

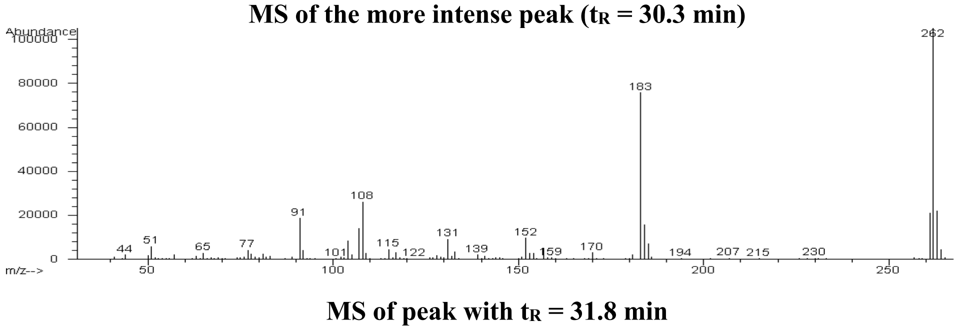

MS of peak with t_R = 31.8 min

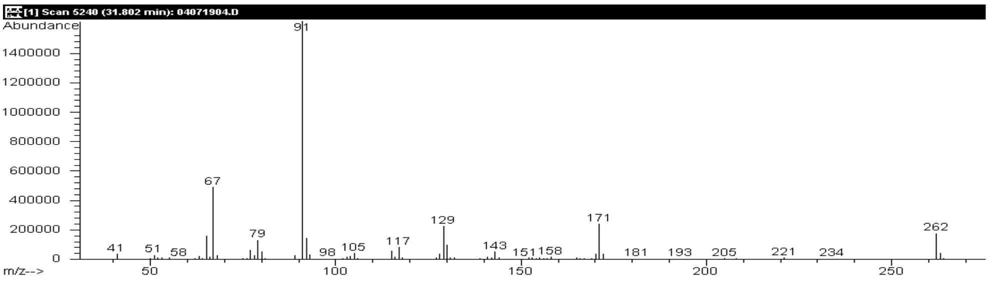

HPLC of the mixture of *ZE*-2 and *EE*-2

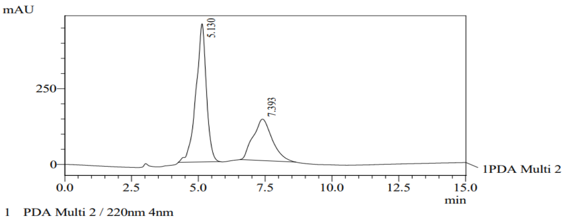

PeakTable

PDA Ch2 220nm 4nm

Peak#	Ret. Time	Area	Height	Area %	Height %
1	5.130	12423598	455591	64.530	76.919
2	7.393	6828799	136706	35.470	23.081
Total		19252398	592297	100.000	100.000

12,14-Hexacosadiene, (*Z,E*)**-4 and** (*E,E*)**-4 mixture**

This dimer was obtained from iodoalkene **3**[S1,S5] (92 mg, 0.30 mmol) following the general procedure. The *ZE*/*EE* ratio of the crude product was determined by GC to be 61:26 (that is, 70:30). A minor peak of an isomer (presumably the *ZZ* isomer, with a *ZE/EE/ZZ* ratio equal to 61:26:8.5 (that is, 64:27:9, nearly) was detected by GC-MS. Purification of the crude mixture by column chromatography on silica gel, with hexane as the eluent, afforded 43 mg (80% yield) of an oil, which according to ^{1}H NMR spectroscopy (first spectrum) is mainly a *ZE*-**4** + *EE*-**4** mixture; the possible *ZZ* isomer looks like an impurity). A second chromatography, on alumina, gave the sample the spectrum of which is reproduced at the bottom of this page. HRMS (ESI, 1% HCOOH): calcd for $[M + 1]^+$, $C_{26}H_{51}^+$, 363.3985; found, 363.3981.

400 MHz ^{1}H NMR spectrum of the crude product (4) in CDCl$_3$

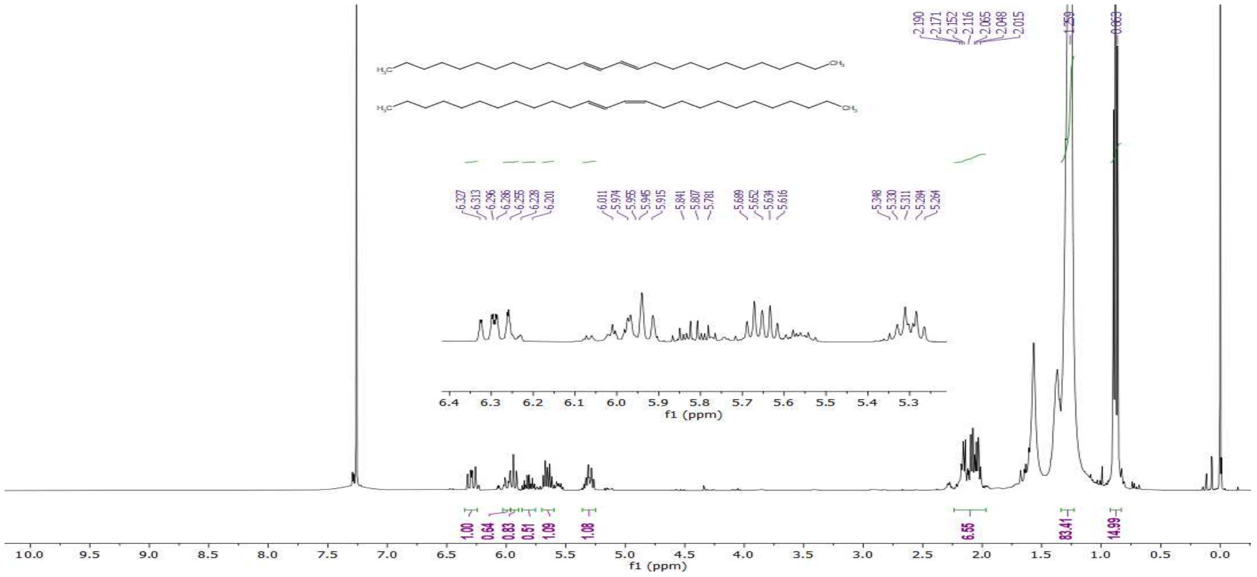

400 MHz ^{1}H NMR spectrum, in CDCl$_3$, of the mixture (4) once purified by chromatography on alumina

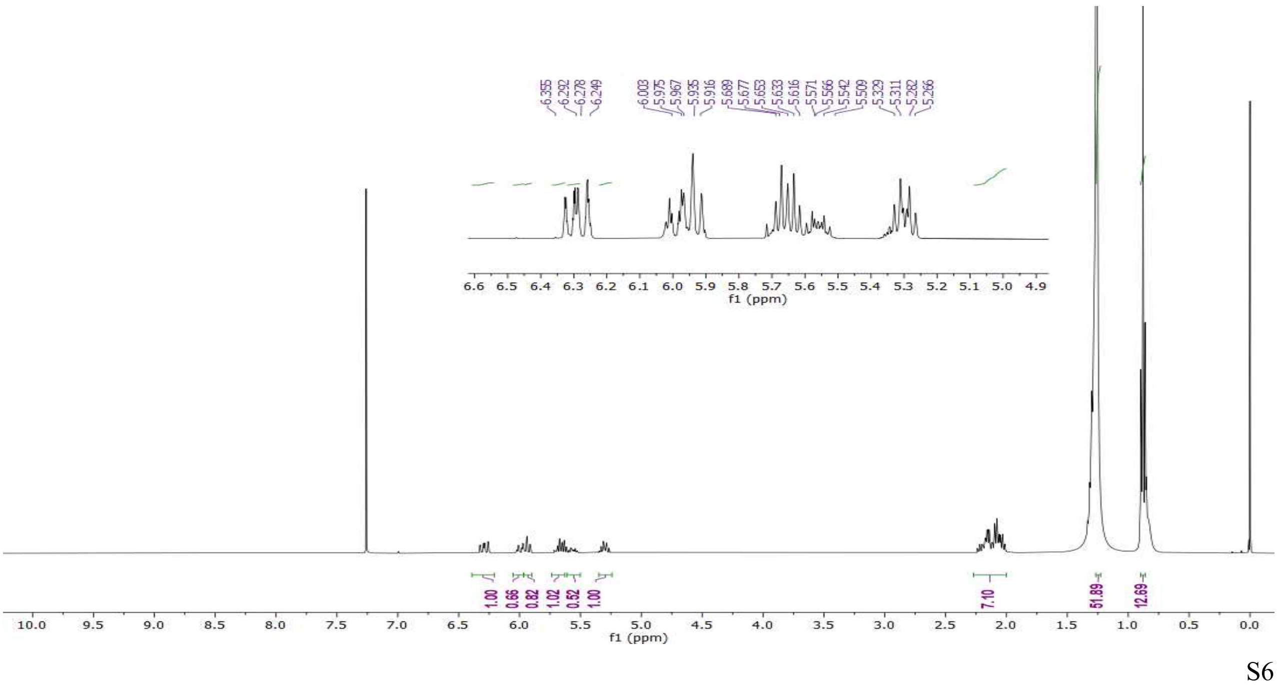

100.6 MHz $^{13}C\{^1H\}$ NMR spectrum of the mixture (4) in $CDCl_3$

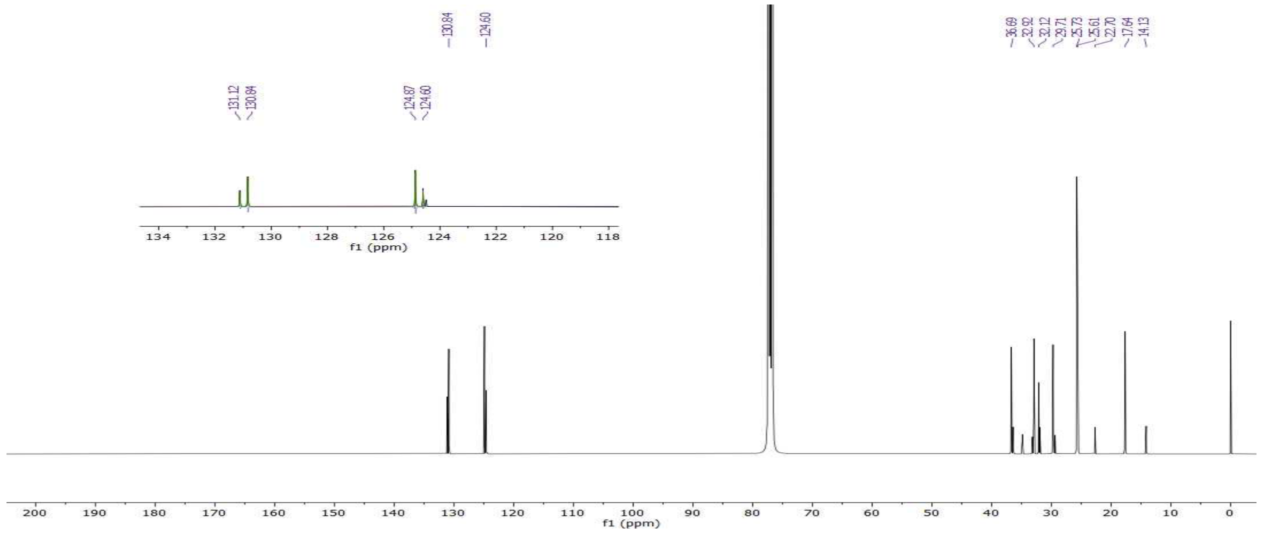

GC-MS of the crude (*Z,E*)-4 and (*E,E*)-4 mixture

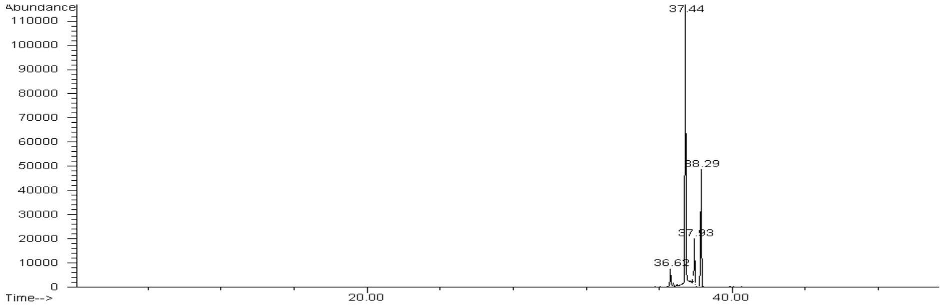

MS of the more intense peak (t_R = 37.4 min)*

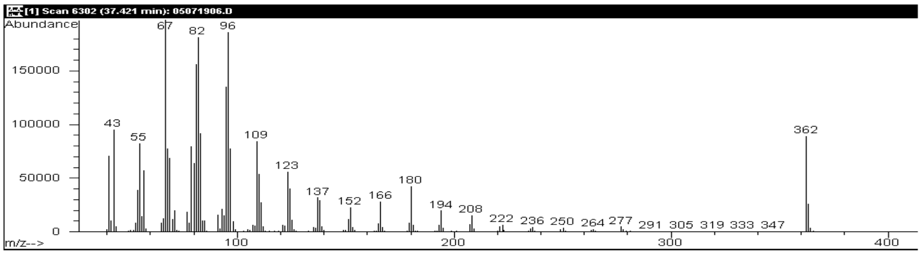

MS of the third peak (t_R = 37.9 min)*

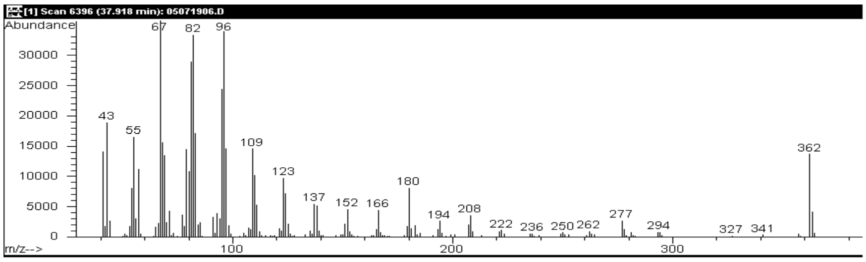

MS of the fourth peak (t_R = 38.3 min)

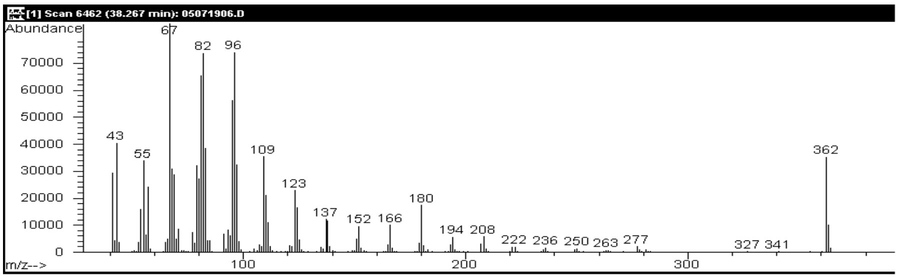

* The first small peak (t_R = 36.6) is an impurity (its mass spectrum does not show the molecular peak at *m/z* 362). By contrast, the third peak, also small (8.5%), might correspond to *ZZ*-**4**, but this isomer was not clearly observed by ^1H NMR after purification of the crude product mixture by chromatography (and disappeared after a second chromatography).

```
                                   Area Percent Report

     Data File : D:\DATA\05071906.D                               Vial: 1
     Acq On    :  5 Jul 2019  13:10                          Operator: jmi
     Sample    : FR 628                                      Inst     : GC/MS Ins
     Misc      :                                             Multiplr: 1.00
                                                        Sample Amount: 0.00
     MS Integration Params: autoint1.e

     Method    : C:\HPCHEM\1\METHODS\ARE1.M (Chemstation Integrator)
     Title     :

     Signal    : EIC Ion 362.00 (361.70 to 362.70)

    peak  R.T. first  max last  PK   peak      corr.   corr.    % of
     #    min   scan scan scan  TY  height     area    % max.   total
    ---  ----- ----- ---- ---- ---  -------   -------  ------  -------
     1  36.622  6132 6151 6164  M      7332    232894   7.15%   4.376%
     2  37.437  6289 6305 6321  M2  115339   3256271 100.00%  61.183%
     3  37.934  6384 6399 6414  M     18173    451883  13.88%   8.491%
     4  38.283  6449 6465 6477  M2   48521   1381138  42.41%  25.951%


                          Sum of corrected areas:     5322186

     05071906.D  ARE1.M      Mon Jul 08 11:35:48 2019   KAYAK
```

(*S,S*)-2,6,13,17-Tetramethyl-2,8,10,16-octatetraene, (*Z,E*)-**6 and** (*E,E*)-**6 mixture.**
Obtained from (*S*)-1-iodo-4,8-dimethyl-1-nonene, **5**,[S6] prepared from commercially available (*S*)-citronellal. Yellowish oil. HRMS (ESI, 1% HCOOH): calcd for $[M + 1]^+$, $C_{22}H_{39}^+$, 303.3046; found, 303.3044.

400 MHz ^{1}H NMR spectrum of the mixture (6) in $CDCl_3$

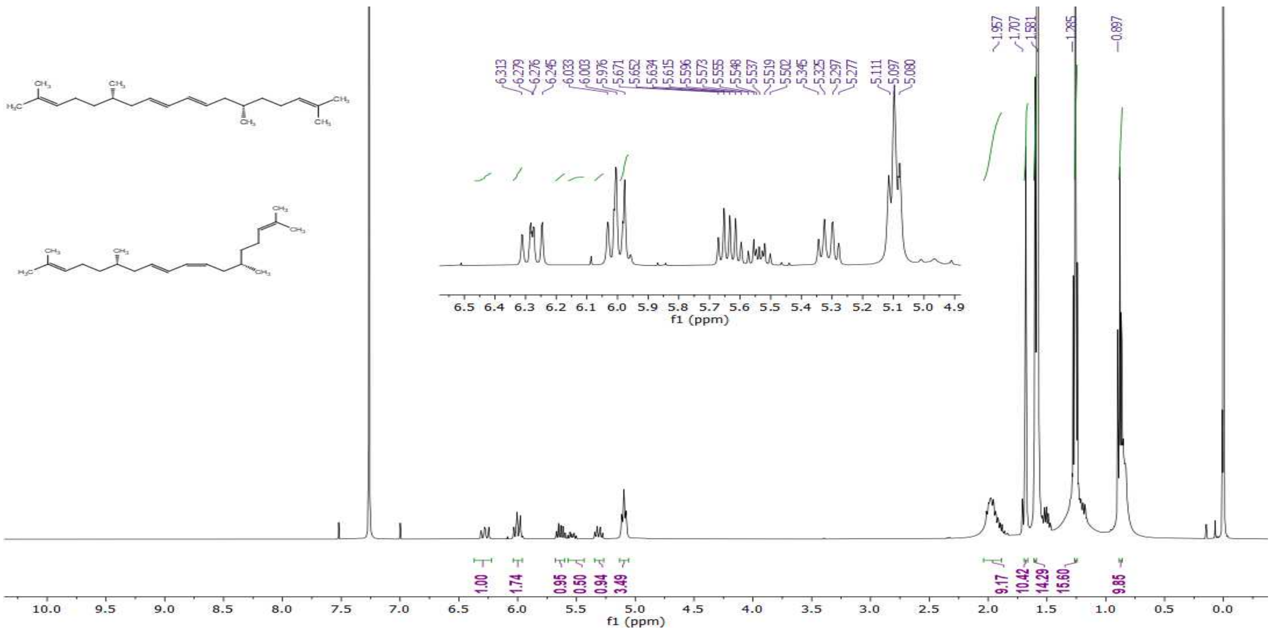

100.6 MHz $^{13}C\{^1H\}$ NMR spectrum of the mixture (6) in $CDCl_3$

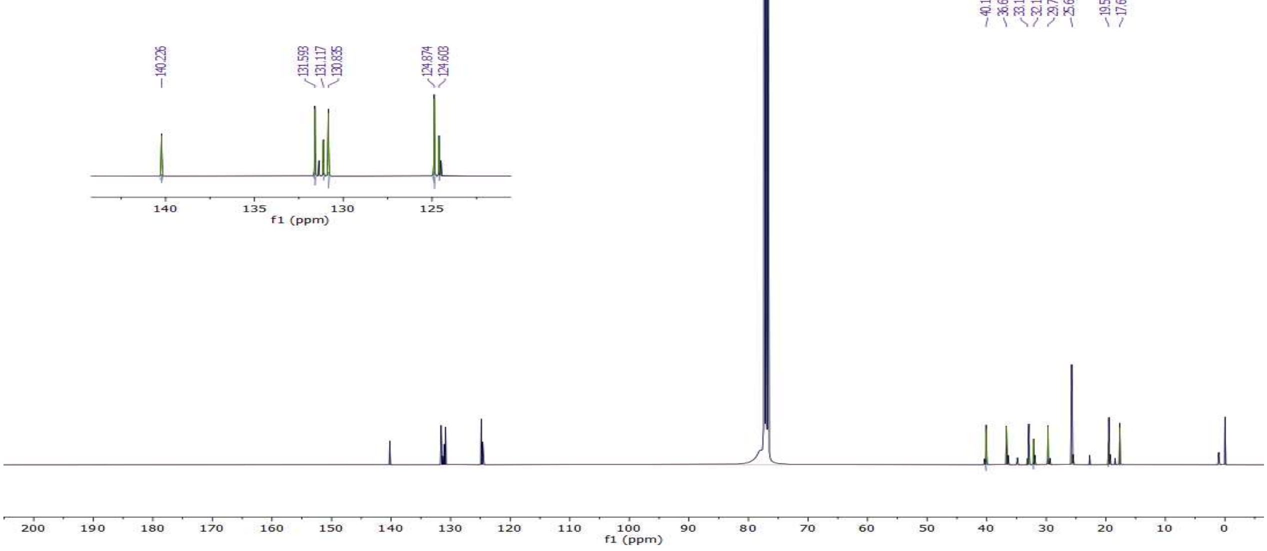

400 MHz ^{1}H NMR spectra of the crude mixture of *ZE*-8 and *EE*-8[S8] in $CDCl_3$

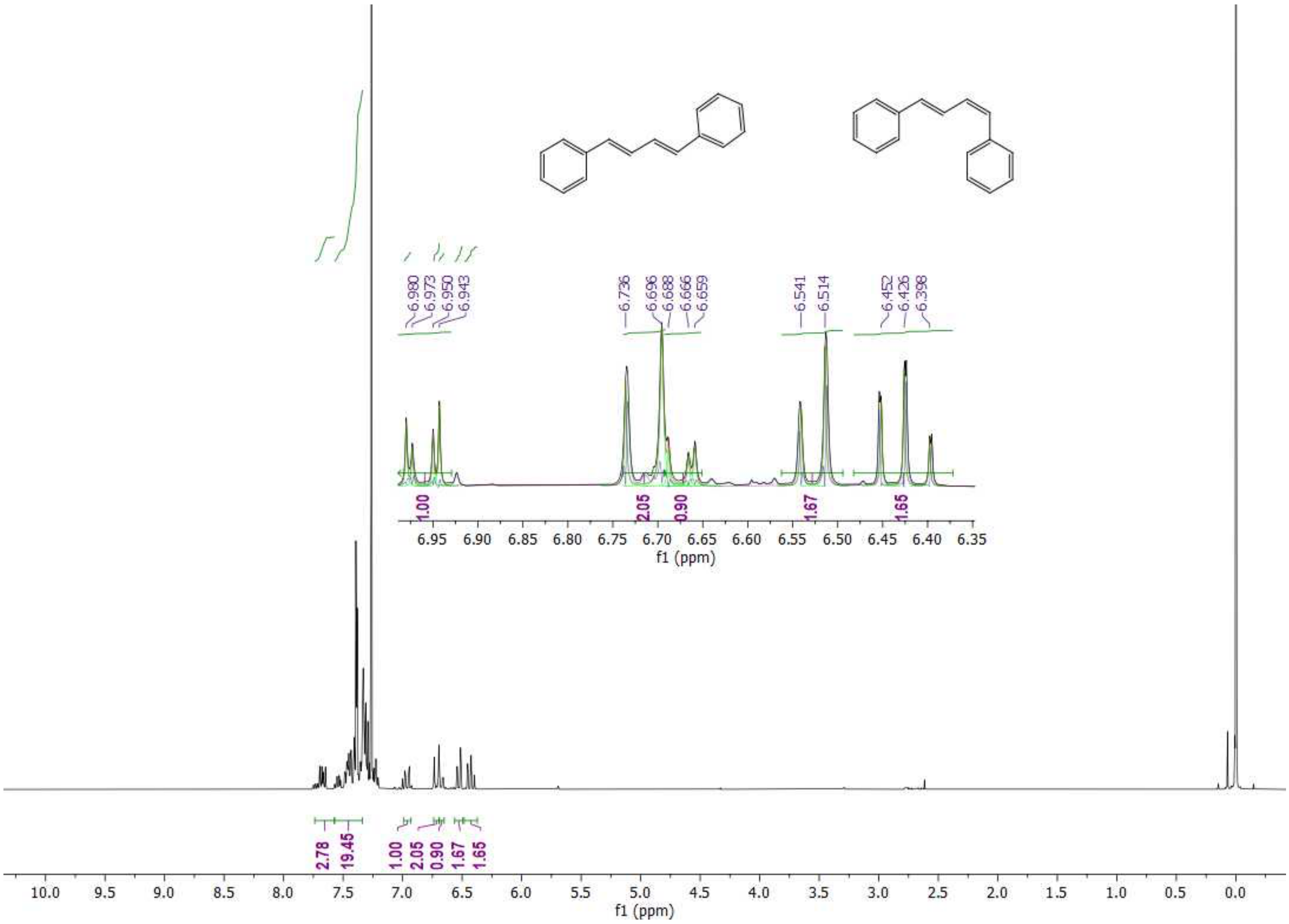

100.6 MHz ^{13}C{^{1}H} NMR spectrum of 8 (mixture of *ZE*-8 and *EE*-8)[S8] in $CDCl_3$

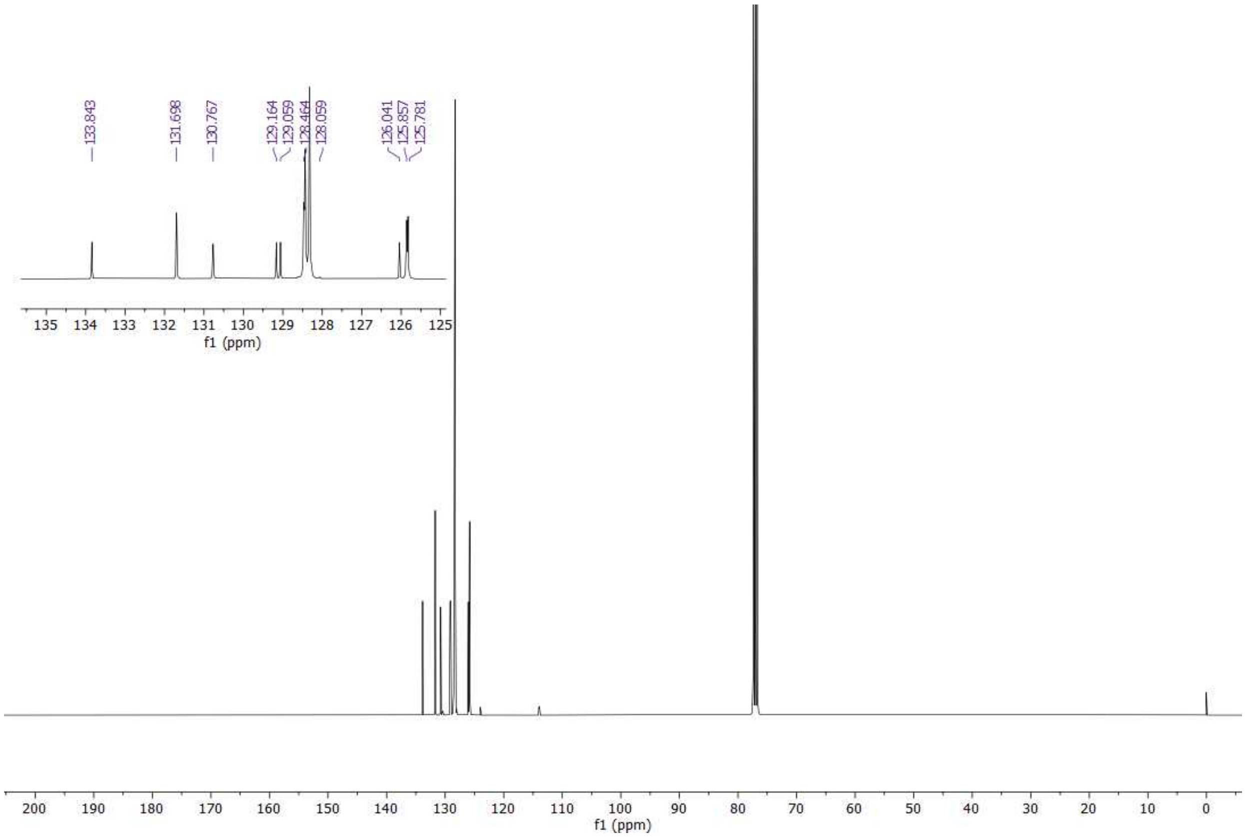

GC-MS of the crude mixture (8)

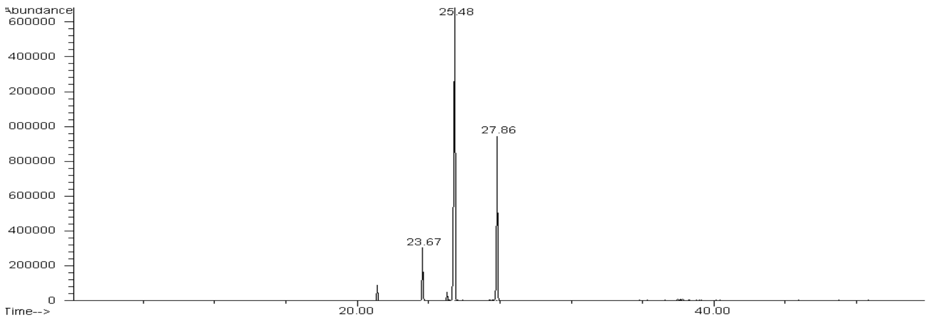

MS of the peak with t_R = 23.7 min*

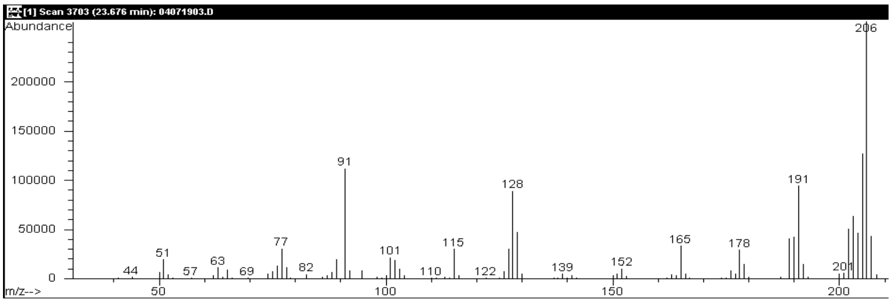

* This peak (6.9% of the mixture) may belong to *ZZ*-**10**, which is however difficultly detected in the ^{1}H NMR reproduced above.

MS of the more intense peak (t_R = 25.5 min)

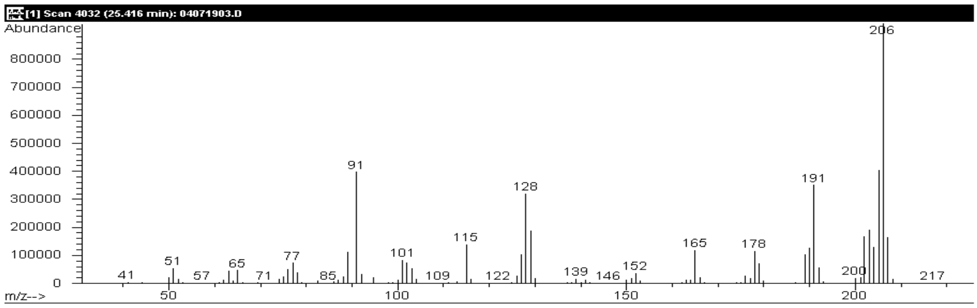

MS of peak with t_R = 27.9 min

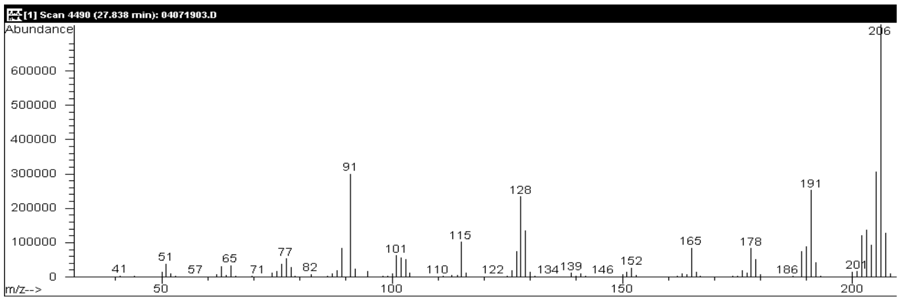

Area Percent Report

Data File : D:\DATA\04071903.D Vial: 1
Acq On : 4 Jul 2019 12:49 Operator: jmi
Sample : FR 631 Inst : GC/MS Ins
Misc : Multiplr: 1.00
Sample Amount: 0.00
MS Integration Params: autoint1.e

Method : C:\HPCHEM\1\METHODS\ARE1.M (Chemstation Integrator)
Title :

Signal : EIC Ion 206.00 (205.70 to 206.70)

peak #	R.T. min	first scan	max scan	last scan	PK TY	peak height	corr. area	corr. % max.	% of total
1	23.671	3684	3702	3717	BB 2	300184	9193816	10.46%	6.902%
2	25.480	4009	4044	4062	BB	1680712	87896347	100.00%	65.984%
3	27.860	4467	4494	4511	BB	942483	36118075	41.09%	27.114%

Sum of corrected areas: 133208238

04071903.D ARE1.M Mon Jul 08 10:36:59 2019 KAYAK

400 MHz ^{1}H NMR spectra of 10 (crude mixture of *ZE*-10 and *EE*-10)[S8,S10] in $CDCl_3$

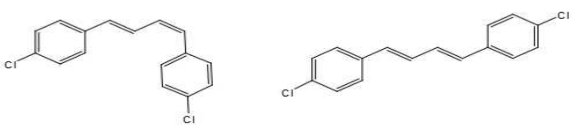

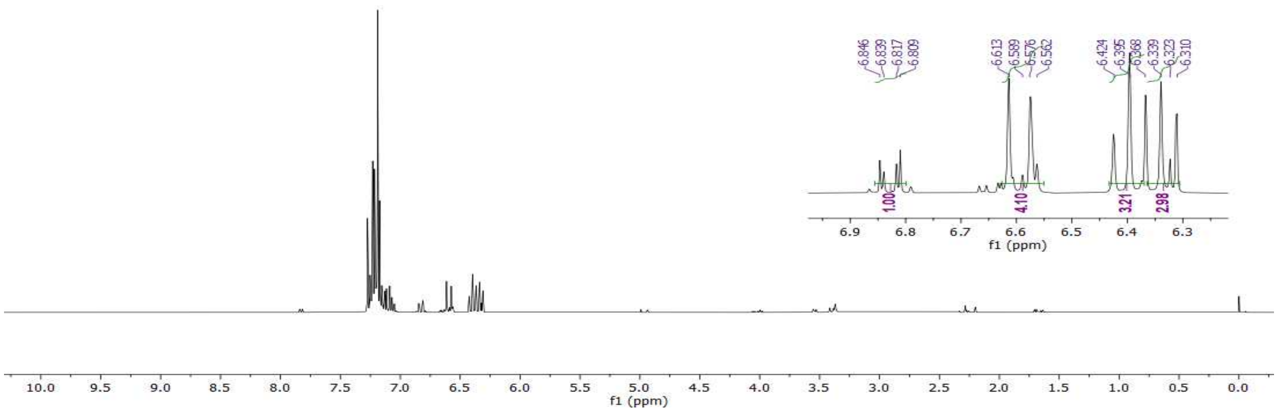

The *ZE/EE* ratios obtained from different experiments were not constant, as it can be observed in this case, in which isomer *ZE* predominated much more than usual. In Table 1 of the main text, however, we indicate mean ratios from different experiments and analytical techniques.

100.6 MHz $^{13}C\{^1H\}$ NMR spectrum of 10 (mixture of *ZE*-10 and *EE*-10) in $CDCl_3$

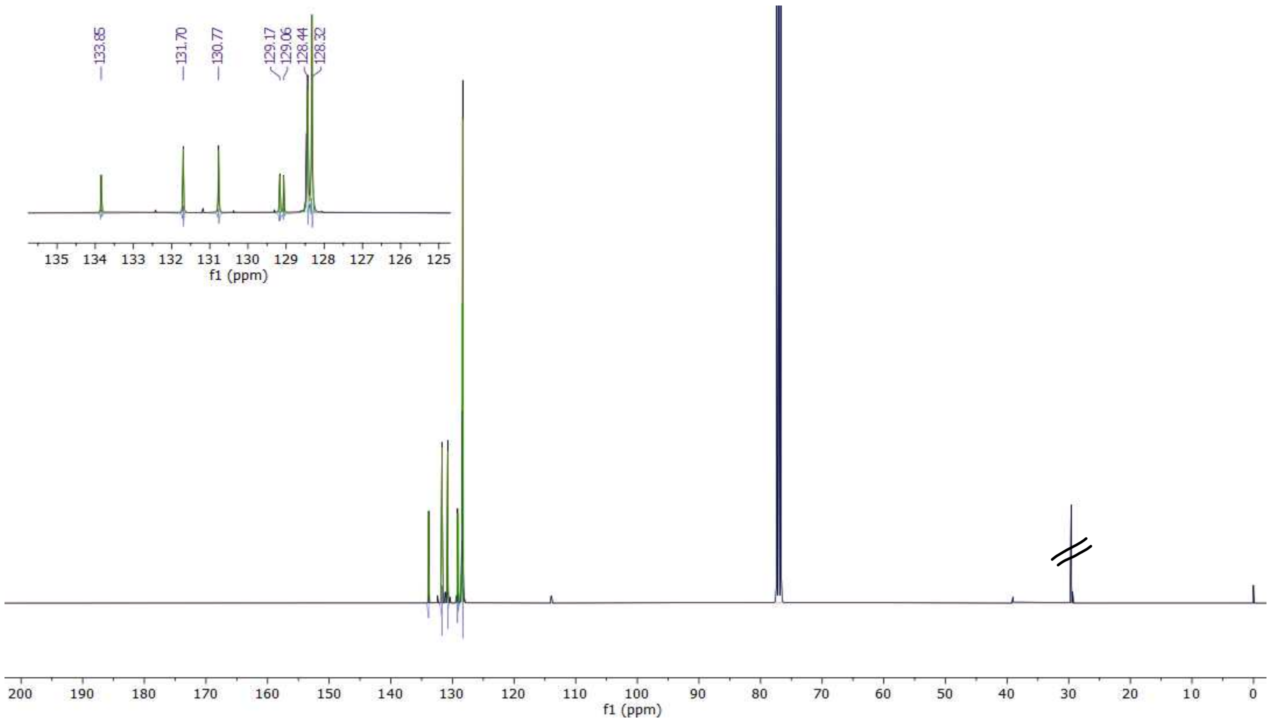

Acetone (from the NMR tube) is observed as an impurity.

GC-MS of the crude product mixture

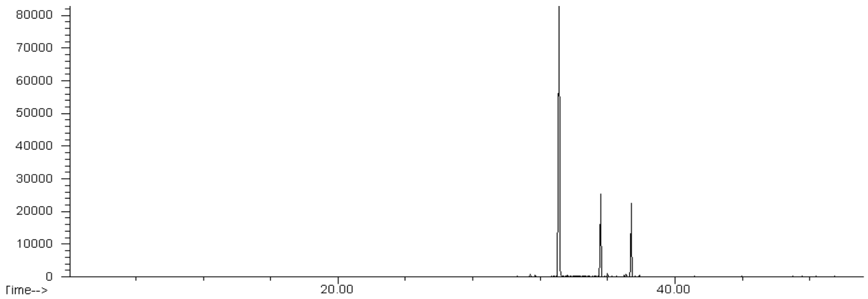

MS of peak 1 (the more intense peak)

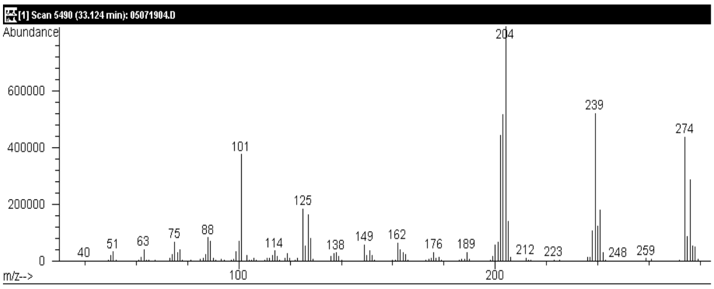

MS of peak 2

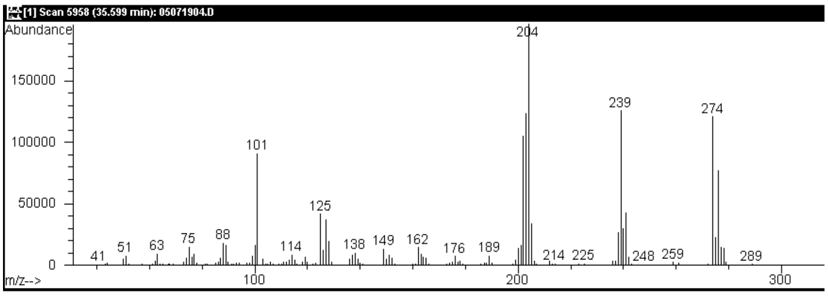


```
                              Area Percent Report

Data File : D:\DATA\05071904.D                          Vial: 1
Acq On    :  5 Jul 2019  11:10                       Operator: jmi
Sample    : FR 632                                   Inst    : GC/MS Ins
Misc      :                                          Multiplr: 1.00
                                                Sample Amount: 0.00
MS Integration Params: autoint1.e

Method    : C:\HPCHEM\1\METHODS\ARE1.M (Chemstation Integrator)
Title     :

Signal    : EIC Ion 275.00 (274.70 to 275.70)

peak  R.T. first  max last  PK   peak      corr.   corr.    % of
 #    min   scan scan scan  TY  height     area    % max.   total
---  ----- ----- ---- ---- ---  -------   -------  ------  -------
 1  33.145  5460 5494 5525  M2  103830   4754424 100.00%  76.082%
 2  35.589  5936 5956 5980  M    25316    847076  17.82%  13.555%
 3  37.483  6274 6299 6332  M2   22486    647585  13.62%  10.363%

                    Sum of corrected areas:     6249085

05071904.D  ARE1.M      Mon Jul 08 11:09:11 2019   KAYAK
```

* The third peak is an impurity (not an isomer of **10**).

400 MHz ^{1}H NMR spectrum of (*E*)-3-benzyloxy-1-iodo-1-propene (11)[S11] in $CDCl_3$

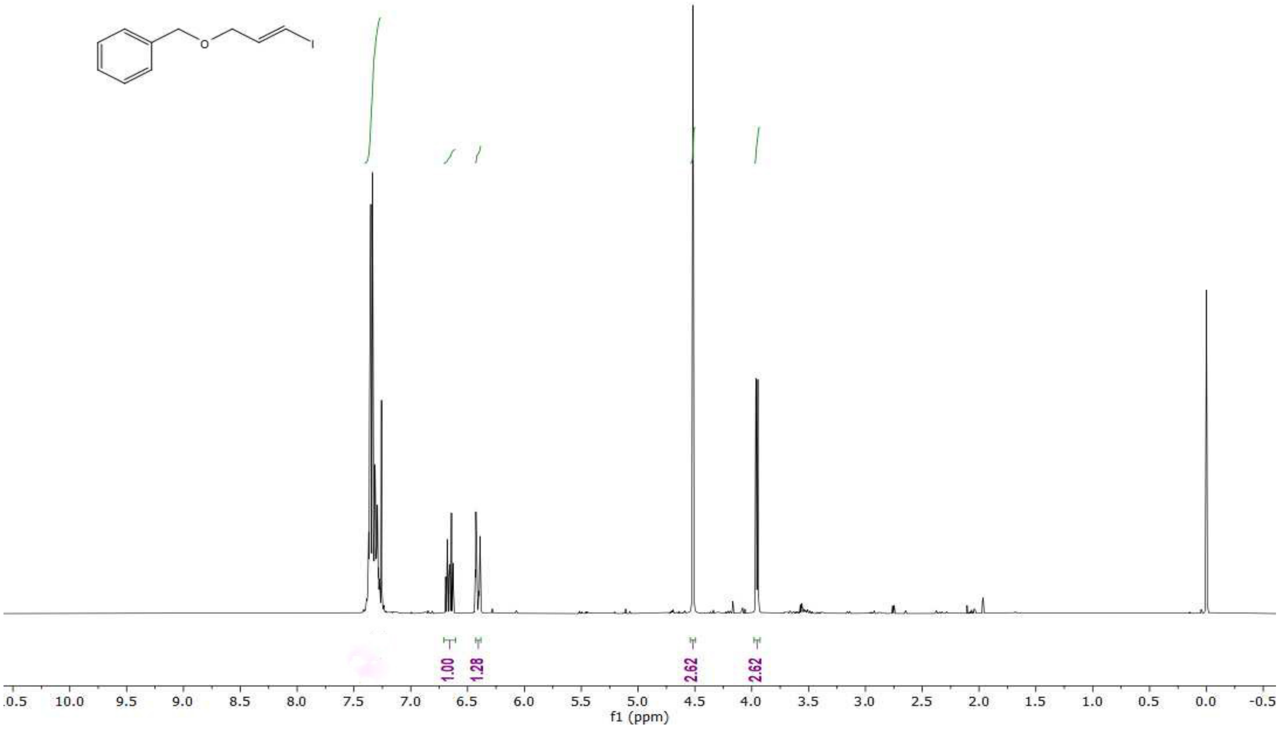

400 MHz ^{1}H NMR spectrum of 1,6-bis(benzyloxy)-2,5-hexadiene (crude mixture of *ZE*-12 and *EE*-12)[S12] in $CDCl_3$

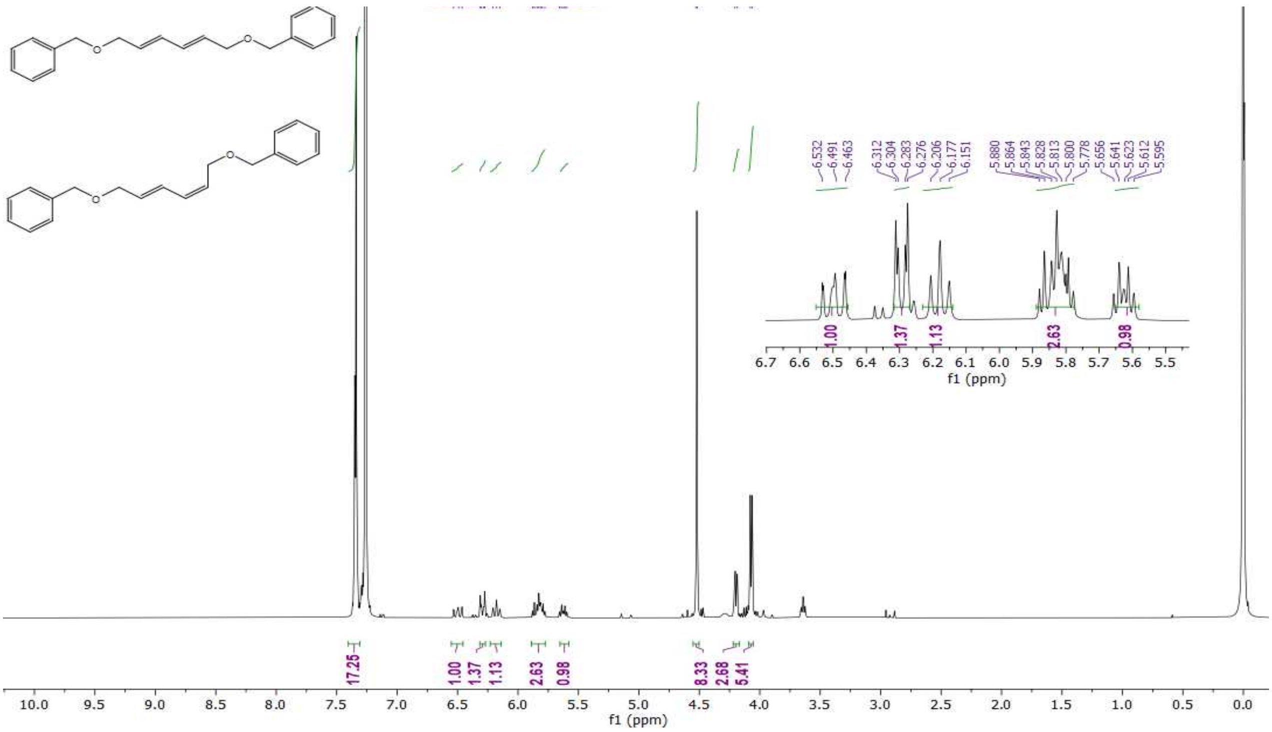

GC of 12 (mixture of *ZE*-12 and *EE*-12)

```
Data Path: C:\msdchem\1\data\2023\ Data File: 22092023-03.D
Acq On: 22 Sep 2023  11:21
Sample: 76
Integration Parameters: autoint1.e Integrator: ChemStation
Method: C:\msdchem\1\methods\SCV1.M Title:
Signal: TIC: 22092023-03.D\data.ms
```

peak #	R.T. min	first scan	max scan	last scan	PK TY	peak height	corr. area	corr. % max.	% of total
1	14.499	1677	1682	1688	M	267995	3067866	19.72%	16.469%
2	15.169	1774	1781	1801	M	1009307	15560372	100.00%	83.531%

```
Sum of corrected areas: 18628238 SCV1.M Wed Sep 27 10:02:29 2023
```

The base peaks of the two spectra (EI, of such a CG-MS experiment) were the benzyl cation ($C_7H_7^+$, *m/z* 91), as it could be expected. Molecular peaks were hardly observed. However, the electrospray ionization MS (HRMS, ESI+) showed peaks at *m/z* 312.1969, calcd for $[M + NH_4]^+$ 312.1958, and *m/z* 317.1528, calcd for $[M + Na]^+$ 317.1512, in accordance with the dimeric structure of the products.

Calculation of Energies of *E* and *Z* Alkenylmetal Halides and of *EE*/*ZE*/*ZZ* Dialkenylmetal Intermediates[S13]

Table S1 shows in the first row the M06/6-311+G(d,p) total energies in au/a.u., or Hartrees, with geometries optimized at such a level unless otherwise indicated, for series of *E* and *Z* isomers of alkenylmetal derivatives. The lowest-energy conformer of each species is depicted; when there are two or more conformers with similar energies, all of them are drawn, if the comparison seems relevant. Other calculations [M06-L, other DFT, MP2, CCSD(T), etc.] are often written below those with M06/6-311+G(d,p). Top-level CCSD(T) calculations have generally confirmed the results at lower levels: since M06 and MP2 methods are sometimes prone to slightly overestimate the van der Waals forces (dipole–dipole, dipole–induced dipole, induced dipole–induced dipole), we have repeated a few calculations at the CCSD(T) level to confirm whether the *Z* intermediates are also predicted to be thermodynamically more stable than, or as stable as, the *E* intermediates. The basis set is 6-311+G(d,p), unless otherwise indicated. Thus, **in Table S1, M06 means M06/6-311+G(d,p), M06-L means M06-L/6-311+G(d,p), ωB97X-D means ωB97X-D/6-311+G(d,p), B3LYP-D3 means B3LYP-D3/6-311+G(d,p), MP2 means MP2/6-311+G(d,p), and CCSD(T) or CCSD mean CCSD(T)/6-311+G(d,p)**.

For Pd and I-containing compounds (for elements > Kr), the LANL2DZ basis set was used for these elements (Spartan'20); the M06·LANL2DZ//M06/6-31G(d)·LANL2DZ energies are of course approximate since the f shells are not considered, but what matters are the relative values. With Gaussian 16, the energies come from the M06·LANL2DZ(Pd or I)//M06/6-31G(d)·LANL2DZ(Pd or I), M06·LANL2DZ, M06·SDD(Pd or I)//M06/6-31G(d)·SDD(Pd or I) or M06·SDD calculations, as indicated.

Calculations of the effect of solvents with the CPCM model on the relative energies (insignificant) and of G^o values (changes often irrelevant), were obtained with Spartan'20 and, when indicated, with Gaussian 16 (Gauss). The values obtained with these two packages are not identical, but the relative energies are coincident. Some calculations were repeated with the just-released Spartan'24 for iMac, affording the same results as Spartan'20 (as expected); in Table S1 they are labeled with an asterisk (as Spa'24).

Some calculations with the Ahlrichs def2-TZVP and def2-TZVPPD basis sets for species containing very large atoms are also included, with similar results to those obtained with the 6-311+G(d,p)·LANL2DZ basis sets. In a few cases, including the derivatives of **1** (see $PhCH_2CH_2CH=CHZnX$, row/box #6), we have confirmed that other DFT calculations, such as those carried out with the ωB97X-D and B3LYP-D3 methods,[S13] afford similar energy differences between *Z* and *E* isomers.

Table S1 (Extended Table 2). Relative Energies (kcal/mol) in Red Bold (Gas Phase) or Blue Bold (Solvent, CPCM)

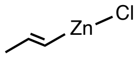<br>–2356.84115* **0.7** **0.7** Gaussian 16<br>(G^o .80208* **0.7**) (G^o **0.75** Gaussian 16)<br>–2356.85794 **0.7** THF<br>–2356.86066 **0.6** DMF<br>–2356.90480 **0.7** M06-2X **0.7** Gauss<br>–2357.01262 **0.5** ωB97X-D **0.5** Gauss<br>–2357.08027 **0.7** B3LYP-D3 **0.7** Gauss<br>–2354.76136 **0.1** MP2<br>–2354.80351 **0.1** CCSD(T)/M06<br>–2354.80433 **0.2** CCSD(T)/MP2<br>*–2356.84115 (G^o.80208) Spa'24	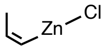<br>–2356.84224* **0.0**<br>(G^o .80318 **0.0**)<br>–2356.85900 **0.0** THF<br>–2356.86173 **0.0** DMF<br>–2356.90587 **0.0** M06-2X<br>–2357.01334 **0.0** ωB97X-D<br>–2357.08134 **0.0** B3LYP-D3<br>–2354.76150 **0.0** MP2<br>–2354.80365 **0.0** CCSD(T)/M06<br>–2354.80458 **0.0** CCSD(T)/MP2<br>*–2356.84224 (G^o .80318) Spa'24	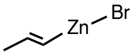<br>–4470.66787* **0.9**<br>–4470.88228 **0.7** M06-2X<br>–4470.62732 **0.7** M06-L<br>–4467.61476 **0.1** MP2<br>–4467.65562 **0.1** CCSD(T)/M06<br>*–4470.66787 Spa'24	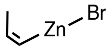<br>–4470.66892* **0.0**<br>–4470.88336 **0.0** M06-2X<br>–4470.62848 **0.0** M06-L<br>–4467.61489 **0.0** MP2<br>–4467.65579 **0.0** CCSD(T)/M06<br>*–4470.66892 Spa'24	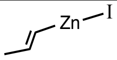<br>–8815.91771 **0.7**<br>–8815.93194 **0.7** THF<br>–8815.93419 **0.8** DMF<br>–2194.43296 **0.6** M06/def2-TZVPPD	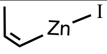<br>–8815.91881 **0.0**<br>–8815.93302 **0.0** THF<br>–8815.93542 **0.0** DMF<br>–2194.43388 **0.0**
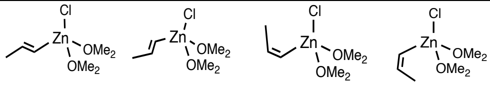<br>*syn*/*s-cis*(Cl/C=C) *anti*/*s-trans*(Cl/C=C)<br>–2666.81521 **1.7** **1.5** Gauss .81672 **0.7**<br>–2667.04616 **1.7** M06-L .04761 **0.8**	*syn* *anti*<br>–2666.81753 **0.2** –2666.81785 **0.0**<br>–2667.04785 **0.7** M06-L .04892 **0.0**	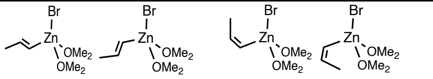<br>*syn* *anti*<br>–4780.64033 **1.4** –4780.64240 **0.1**<br>–4780.85388 **1.0** M06-L .85592 **0.1**	*syn* *anti*<br>–4780.64254 **0.0** –4780.64242 **0.1**<br>–4780.85548 **0.4** M06-L .85609 **0.0**	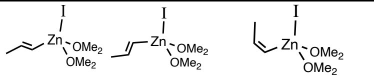<br>*syn* *anti*<br>–9125.89000 **0.9** –9125.89129 **0.1**<br>(G^o.70198) **0.5** (G^o.70128) **1.0**	–9125.89150 **0.0**<br>(G^o.70281) **0.0**
THF instead of Me_2O, *syn* *anti*<br>–2821.58597 **1.8** –2821.58801 **0.5**<br>–2818.47894 **2.3** MP2 –2818.48150 **0.7**<br>CCSD/6-31G(d)//MP2 –2815.84836 **1.2**	THF instead of Me_2O, *syn* *anti*<br>–2821.58878 **0.0** –2821.58753 **0.8**<br>–2818.48106 **1.0** MP2 –2818.48259 **0.0**<br>CCSD/6-31G(d)//MP2 –2815.85035 **0.0**	THF instead Me_2O, *syn* *anti*<br>–4935.41106 **1.7** –4935.41291 **0.6**	THF instead of Me_2O, *syn* *anti*<br>–4935.41382 **0.0** –4935.41278 **0.7**		
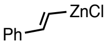<br>–2548.47560 **1.0**<br>–2548.67810 **0.8** M06-L<br>–2548.61522 **1.9** M06-2X<br>–2545.97891 **1.8** MP2	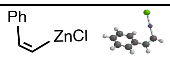<br>–2548.47717 **0.0**<br>–2548.67934 **0.0** M06-L<br>–2548.61831 **0.0** M06-2X<br>–2545.98184 **0.0** MP2	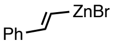<br>–4662.30222 **0.9**<br>–4658.83198 **1.2** MP2	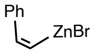<br>–4662.30372 **0.0**<br>–4658.83384 **0.0** MP2	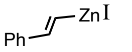<br>–9007.55224 **0.9**	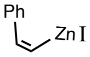<br>–9007.55224 **0.0**
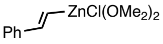<br>–2858.34213 **4.6**<br>–2858.70769 **4.6** M06-L<br>–2858.57328 **6.0** M06-2X	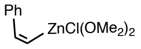<br>–2858.34951 **0.0**<br>–2858.71505 **0.0** M06-L<br>–2858.58280 **0.0** M06-2X	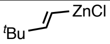<br>–2474.71873 **1.4**<br>–2474.77200 **2.0** M06-L<br>–2474.73217 **2.1** M06-2X	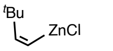<br>–2474.72103 **0.0**<br>–2474.77511 **0.0** M06-L<br>–2474.73545 **0.0** M06-2X	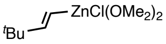<br>–2784.58409 **2.0**<br>–2784.90066 **2.7** M06-L	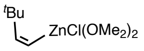<br>–2784.58727 **0.0**<br>–2784.90491 **0.0** M06-L
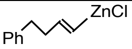<br>–2627.05395 **0.9** **1.0** Gaussian 16<br>(G^o.91198 **0.5**) (G^o **0.3** Gaussian 16)<br>–2627.21973 **0.9** M06-2X<br>–2627.34969 **0.8** ωB97X-D<br>–2627.52310 **1.1** B3LYP-D3<br>–2624.36757 **1.1** MP2	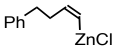<br>–2627.05541 **0.0**<br>(G^o.91282 **0.0**)<br>–2627.22114 **0.0** M06-2X<br>–2627.35098 **0.0** ωB97X-D<br>–2627.52488 **0.0** B3LYP-D3<br>–2624.36928 **0.0** MP2	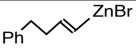<br>–4740.88054 **0.9**	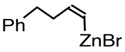<br>–4740.88192 **0.0**	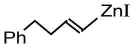<br>–9086.13044 **1.0**	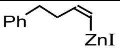<br>–9086.13206 **0.0**
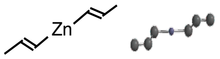<br>–2013.82212 **1.1**<br>(G^o .71779 **0.8**)<br>–2013.83425 **1.4** DMF<br>(G^o .73091 **0.8**) DMF<br>–2012.00324 **0.0** MP2<br>–2012.08212 **0.0** CCSD(T)/MP2	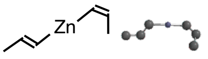<br>–2013.82270 **0.7**<br>(G^o .71902 **0.0**)<br>–2013.83500 **0.2** DMF<br>(G^o .73186 **0.2**) DMF<br>–2012.00309 **0.1!** MP2<br>–2012.08208 **0.0** CCSD(T)/MP2	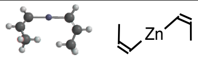<br>–2013.82388 **0.0** .82302 **0.5**<br>(G^o.71892 **0.1**) (G^o.71874 **0.2**)<br>–2013.82650 **0.0** DMF<br>(G^o .73221 **0.0**) DMF<br>–2012.00302 **0.1!** MP2 .00247 **0.5**<br>–2012.08212 **0.0** CCSD(T)/MP2	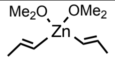<br>–2323.78692 **1.3**<br>(G^o .53089 **1.0**)<br>–2321.28740 **0.0** MP2	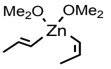<br>–2323.78710 **1.2**<br>(G^o .53158 **0.6**)<br>–2321.28684 **0.35!** MP2	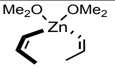<br>–2323.78907 **0.0**<br>(G^o .53247 **0.0**)<br>–2321.28697 **0.3!** MP2

(E)-CH₃CH=CH–Li
–124.73040 0.5 0.5 Gaussian 16
(*G*° .72083 0.6)
–124.74900 0.4 THF
–124.75159 0.4 DMF
–124.38579 0.2 MP2
–124.43968 0.2 CCSD//B3LYP/6-31G(d)
–124.43924 0.2 CCSD(T)//M06
–124.43991 0.2 CCSD(T)//MP2

(Z)-CH₃CH=CH–Li
–124.73113 0.0
(*G*° .72174 0.0)
–124.74964 0.0 THF
–124.75228 0.0 DMF
–124.38612 0.0 MP2
–124.44004 0.0 CCSD//B3LYP/6-31G(d)
–124.43963 0.0 CCSD(T)//M06
–124.44029 0.0 CCSD(T)//MP2

(E)-^{t}Bu–CH=CH–Li
–242.60854 3.1
–242.77065 3.3 M06-L
–241.98182 2.8 MP2
–241.91169 2.6 CCSD/6-31+G(d)//M06
–242.08592 2.9 CCSD(T)//M06
–242.08691 2.9 CCSD(T)//MP2

(Z)-^{t}Bu–CH=CH–Li
–242.61341 0.0
–242.77589 0.0 M06-L
–241.98632 0.0 MP2
–241.91585 0.0 CCSD/6-31+G(d)/M06
–242.09044 0.0 CCSD(T)//M06
–242.09146 0.0 CCSD(T)//MP2

(E)-Ph–CH=CH–Li
–316.36910 4.3
–316.58002 3.7 M06-L
dimer (*EE* aggregate):
–632.81263 5.3 M06//M06/6-31G(d)

(Z)-Ph–CH=CH–Li
–316.37596 0.0
–316.58597 0.0 M06-L
dimer (*ZE* aggregate):
–632.81655 2.8
dimer (*ZZ* aggregate):
–632.82109 0.0

(E)-CH₃CH=CH–Li(OMe₂)₃
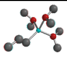
–589.70586 0.9
–589.72160 1.0 THF
–589.72444 1.5 DMF
–588.34097 0.5 MP2//MP2/6-31+G(d)
–588.34137 0.5 MP2

(Z)-CH₃CH=CH–Li(OMe₂)₃
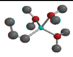
–589.70704 0.0
–589.72320 0.0 THF
–589.72688 0.0 DMF
–588.34097 0.0 MP2//MP2/6-31+G(d)
–588.34216 0.0 MP2

(E)-^{t}Bu–CH=CH–Li(OMe₂)₃
–707.58508 0.3
–707.97789 0.5 M06-L
–707.75009 0.1 M06-2X

(Z)-^{t}Bu–CH=CH–Li(OMe₂)₃
–707.58549 0.0
–707.97875 0.0 M06-L
–707.75020 0.0 M06-2X

(E)-Ph–CH=CH–Li(OMe₂)₃
–781.34589 3.0
–781.78758 3.7 M06-L
–781.54918 4.0 M06-2X

(Z)-Ph–CH=CH–Li(OMe₂)₃
–781.35072 0.0
–781.79346 0.0 M06-L
–781.55556 0.0 M06-2X

(E)-CH₃CH=CH–MgCl
–777.57158 0.9 0.9 Gaussian 16
(*G*° 53270 0.9)
–777.59082 0.5 THF
–777.59391 0.6 DMF
–777.60411 0.7 M06-2X
(*G*° .56370 0.8)
–776.31667 0.1 MP2
–776.38642 0.1 CCSD(T)//M06-2X
–776.38628 0.0 CCSD(T)//M06
–776.38700 0.1 CCSD(T)//MP2

(Z)-CH₃CH=CH–MgCl
–777.57296 0.0
(*G*° 53418 0.0)
–777.59170 0.0 THF
–777.59479 0.0 DMF
–777.60521 0.0 M06-2X
(*G*° .56502 0.0)
–776.31678 0.0 MP2
–776.38657 0.0 CCSD(T)//M06-2X
–776.38634 0.0 CCSD(T)//M06
–776.38723 0.0 CCSD(T)//MP2

(E)-CH₃CH=CH–MgBr
–2891.39447 0.7
–2891.41240 0.6 THF
–2891.41534 0.6 DMF
–2891.40459 0.9 M06/6-311+G(2df,2p)//M06
–2891.37022 0.8 M06/6-311+G(3df,2p)//M06

(Z)-CH₃CH=CH–MgBr
–2891.39586 0.0
–2891.41329 0.0 THF
–2891.41635 0.0 DMF
–2891.40596 0.0 M06/6-311+G(2df,2p)//M06
–2891.37155 0.0 M06/6-311+G(3df,2p)//M06

(E)-CH₃CH=CH–MgI
–7236.64041 0.9
–7236.65039 0.9 M06/6-311+G(2df,2p)// M06·LANL2DZ
–615.15368 0.9 M06/def2-TZVPPD// M06·LANL2DZ

(Z)-CH₃CH=CH–MgI
–7236.64190 0.0
–7236.65178 0.0 M06/6-311+G(2df,2p)// M06·LANL2DZ
–615.15516 0.0 M06/def2-TZVPPD// M06·LANL2DZ

(E)-CH₃CH=CH–Mg(OMe₂)₂Cl (142.4°)
–1087.57732 0.2
(*G*° .39164 0.1) Gaussian 16
–1087.67507 0.8 M06-2X
(*G*° .48555 0.0) Gaussian 16
–1085.63893 0.1 MP2//MP2/6-31+G(d)
–1085.63921 0.1 MP2
–1085.45910 0.0 CCSD/6-31+G//M06
–1085.46097 0.0 CCSD/6-31+G//MP2
–1085.81115 0.2 CCSD(T)//M06

(Z)-CH₃CH=CH–Mg(OMe₂)₂Cl
–1087.57765 0.0
(*G*° .39181 0.0) Gaussian 16
–1087.67637 0.0 M06-2X
(*G*° .48548 0.0) Gaussian 16
–1085.63907 0.0 MP2//MP2/6-31+G(d)
–1085.63941 0.0 MP2
–1085.45876 0.2! CCSD/6-31+G//M06
–1085.46085 0.1! CCSD/6-31+G//MP2
–1085.81154 0.0 CCSD(T)//M06

(E)-CH₃CH=CH–Mg(OMe₂)₂Br
–3201.39941 0.2
(*G*° .21505 0.0) Gaussian 16
–3198.65660 0.5 CCSD(T)//M06

(Z)-CH₃CH=CH–Mg(OMe₂)₂Br
–3201.39965 0.0
(*G*° .21471 0.2!) Gaussian 16
–3198.65736 0.0 CCSD(T)//M06

(E)-CH₃CH=CH–Mg(OMe₂)₂I
–7546.64491 0.2
–7546.67206 0.1 M06/6-311+G(2df,2p)// M06/6-31G(d)·LANL2DZ
–925.19693 0.0 M06/def2-TZVPPD

(Z)-CH₃CH=CH–Mg(OMe₂)₂I
–7546.64526 0.0
–7546.67228 0.0 M06/6-311+G(2df,2p)// M06/6-31G(d)·LANL2DZ
–925.19694 0.0 M06/def2-TZVPPD

THF instead of OMe₂
–1242.34938 0.5
–1240.06242 0.4 MP2//MP2/6-31+G(d)
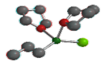

THF instead of OMe₂
–1242.35018 0.0
–1240.06302 0.0 MP2//MP2/6-31+G(d)
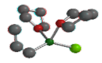

THF instead of OMe₂
–3356.17152 0.5
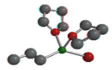

THF instead of OMe₂
–3356.17231 0.0
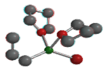

THF instead of OMe₂
–7701.41826 0.3
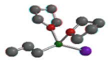

THF instead of OMe₂
–7701.41873 0.0
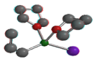

(E,E)-Mg(CH=CHCH₃)₂
–434.52886 0.6
–433.53540 0.0 MP2
–433.64374 0.0 CCSD(T)/MP2

(E,Z)-Mg(CH=CHCH₃)₂
–434.52984 0.0
–433.53533 0.1! MP2
–433.64368 0.0 CCSD(T)/MP2

(Z,Z)-Mg(CH=CHCH₃)₂
–434.53130 –0.9
–433.53531 0.1! MP2
–433.64381 0.0 CCSD(T)/MP2

–434.53060 –0.5

trans-Ni(OMe₂)₂(CH=CHCH₃)₂ (E,E)
–2052.67515 3.1

trans-Ni(OMe₂)₂(CH=CHCH₃)₂ (E,Z)
–2052.67835 1.1

trans-Ni(OMe₂)₂(CH=CHCH₃)₂ (Z,Z)
–2052.67998 0.0

cis-Ni(OMe₂)₂(CH=CHCH₃)₂ (E,E)
–2052.70687 3.4
–2049.92826 1.8 CCSD(T)/ 6-31+G(d)//M06
–2050.46572 1.4 CCSD(T)//M06

cis-Ni(OMe₂)₂(CH=CHCH₃)₂ (E,Z)
–2052.70955 1.7
–2049.92977 0.8 CCSD(T)/ 6-31+G(d)//M06
–2050.46700 0.6 CCSD(T)//M06

cis-Ni(OMe₂)₂(CH=CHCH₃)₂ (Z,Z)
–2052.71223 0.0
–2049.93110 0.0 CCSD(T)/ 6-31+G(d)//M06
–2050.46794 0.0 CCSD//M06

trans-Ni(PPh₃)₂(CH=CHCH₃)₂ (E,E)
–3814.61738 1.8 M06//M06/6-31G(d)
–3814.62108 0.6 M06

trans-Ni(PPh₃)₂(CH=CHCH₃)₂ (E,Z)
–3814.62014 0.0 M06//M06/6-31G(d)
PNiP 170.6°, CNiC 178.8°
–3814.62211 0.0 M06

trans-Ni(PPh₃)₂(CH=CHCH₃)₂ (Z,Z)
–3814.61909 0.7!
–3814.61854 1.0!

cis-Ni(PPh₃)₂(CH=CHCH₃)₂ (E,E)
–3814.61654 2.8 M06//M06/6-31G(d)
PNiP 104.6°, CNiC 78.0°

cis-Ni(PPh₃)₂(CH=CHCH₃)₂ (E,Z)
–3814.61915 1.1 M06//M06/6-31G(d)
PNiP 102.7°, CNiC 77.0°

cis-Ni(PPh₃)₂(CH=CHCH₃)₂ (Z,Z)
–3814.62096 0.0
PNiP 102.6°, CNiC 77.0°

For the Pd compounds the first values (first rows) are M06//M06/6-31G(d), with the LANL2DZ basis set for Pd (and I); 'f shells not fully supported'.

trans-PdCl(PMe₃)₂(E-CH=CHCH₃)
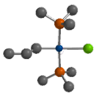
–1627.53673 2.5
–1627.88794 2.5 M06-L
–1626.31220 2.2 M06·LANL2DZ// M06/6-31G(d)·LANL2DZ
–1626.33791 2.5 M06·LANL2DZ
–1627.47848 2.0 M06·SDD// M06/6-31G(d)·SDD
–1627.50233 2.5 M06·SDD
–1627.85123 0.9 ωB97X-D/def2-TZVP
–1627.80745 1.8 ωB97X-D·LANL2DZ
–1622.77542 1.4 MP2/6-31+G(d)
–1624.53461 1.7 MP2//MP2/6-31+G(d)
–1624.53712 1.8 MP2
–1622.94549 1.1 CCSD(T)/6-31G(d)//MP2

trans-PdCl(PMe₃)₂(Z-CH=CHCH₃)
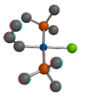
–1627.54074 0.0
–1627.89200 0.0 M06-L
–1626.31570 0.0 M06·LANL2DZ// M06/6-31G(d)·LANL2DZ
–1626.34194 0.0 M06·LANL2DZ
–1627.48172 0.0 M06·SDD// M06/6-31G(d)·SDD
–1627.50632 0.0 M06·SDD
–1627.85260 0.0 ωB97X-D/def2-TZVP
–1627.81036 0.0 ωB97X-D·LANL2DZ
–1622.77764 0.0 MP2/6-31+G(d)
–1624.53735 0.0 MP2//MP2/6-31+G(d)
–1624.53993 0.0 MP2
–1622.94730 0.0 CCSD(T)/6-31G(d)//MP2

cis-PdCl(PMe₃)₂(E-CH=CHCH₃)
–1627.51893 3.2
–1626.29570 1.6 M06·LANL2DZ// M06/6-31G(d)·LANL2DZ
–1626.32011 3.2 M06·LANL2DZ
–1627.45898 3.4 M06·SDD// M06/6-31G(d)·SDD
–1627.48470 3.2 M06·SDD
–1627.83307 1.3 ωB97X-D/def2-TZVP

cis-PdCl(PMe₃)₂(Z-CH=CHCH₃)
–1627.52402 0.0
–1626.29813 0.0 M06·LANL2DZ// M06/6-31G(d)·LANL2DZ
–1626.32516 0.0 M06·LANL2DZ
–1626.46432 0.0 M06·SDD// M06/6-31G(d)·SDD
–1626.48971 0.0 M06·SDD
–1627.83507 0.0 ωB97X-D/def2-TZVP

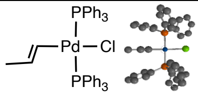<br>–2777.29712 **0.9**<br>–2775.67691 **0.3** M06/6-31G(d)·LANL2DZ<br>–2777.47001 **0.8**<br>M06/def2-TZVP//M06/6-31G(d)	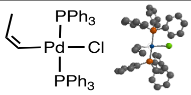<br>–2777.29851 **0.0**<br>–2775.67743 **0.0** M06/6-31G(d)·LANL2DZ<br>–2777.47134 **0.0**<br>M06/def2-TZVP//M06/6-31G(d)	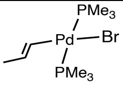<br>–3741.36471 **2.4**<br>–3739.76846 **1.9** M06/6-31G(d)·LANL2DZ<br>(*G*° .51614 **0.8**)	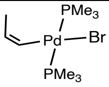<br>–3741.36853 **0.0**<br>–3739.77141 **0.0**<br>(*G*° .51744 **0.0**)	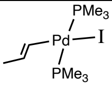<br>–8086.61885 **2.3**<br>–8086.61470 **2.5**<br>M06(3df,2p)·LANL2DZ//<br>M06/6-31G(d)·LANL2DZ	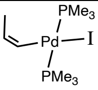<br>–8086.62253 **0.0**<br>–8086.61862 **0.0**<br>M06(3df,2p) LANL2DZ//<br>M06/6-31G(d)·LANL2DZ
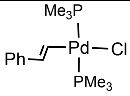<br>–1819.17397 **6.5**	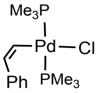<br>–1819.18127 **0.0**		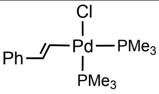<br>–1819.15565 **16.1 (6.4)**	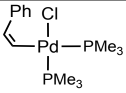<br>–1819.16583 **9.7 (0.0)**	
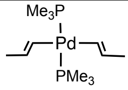<br>–1284.50942 **1.8**<br>–1284.12599* **1.1** M06/6-31G(d)<br>(*G*° .80603* **0.4**)<br>–1284.50972 **1.8** M06 **1.7** Gaussian 16<br>–1279.97311 **1.0** MP2/6-31+G(d)<br>–1281.78091 **1.1** MP2<br>*–1284.12599 (*G*° .80603) Spa'24	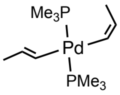<br>–1284.51223 **0.0**<br>–1284.12776* **0.0** M06/6-31G(d)<br>(*G*° .80669* **0.0**)<br>–1284.51262 **0.0** M06<br>–1279.97476 **0.0** MP2/6-31+G(d)<br>–1281.78272 **0.0** MP2<br>*–1284.12599 (*G*° .80669) Spa'24	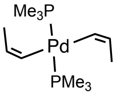<br>–1284.51490 **–1.7**<br>–1284.12910 **–0.8** M06/6-31G(d)<br>(*G*° .80757 **–0.6**)<br>–1284.51521 **–1.6** M06<br>–1279.97584 **–0.7** MP2/6-31+G(d)<br>–1281.78459 **–1.2** MP2	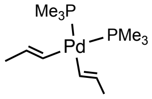<br>–1284.50421 **5.0 (2.1)**<br>–1284.50628 **4.0 (1.0)** M06<br>–1279.97044 **2.7 (1.3)** MP2/6-31+G(d)<br>–1281.77958 **2.0 (1.6)** MP2	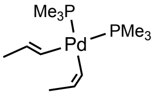<br>–1284.50757 **2.9 (0.0)**<br>–1284.50791 **3.0 (0.0)** M06<br>–1279.97246 **1.4 (0.0)** MP2/6-31+G(d)<br>–1281.78208 **0.4 (0.0)** MP2	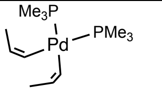<br>–1284.50850 **2.3 (–0.6)**<br>–1284.50968 **1.8 (–1.2)** M06<br>–1279.97215 **1.6 (0.2!)**<br>–1281.78258 **0.1 (–0.3)** MP2
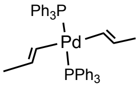<br>–2434.27107 **5.1 (0.2)**	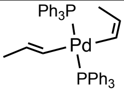<br>–2434.27139 **4.9 (0.0)**	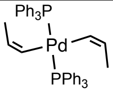<br>–2434.27295 **3.9 (–1.0)**	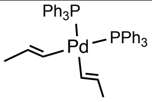<br>–2434.27735 **1.1** **1.2** Gaussian 16<br>(*G*° –2432.47930 **1.3** Gaussian 16)<br>–2432.65070 **0.5** M06/6-31G(d)<br>–2435.08157 **1.4** ωB97X-D<br>–2435.78129 **1.3** B3LYP-D3	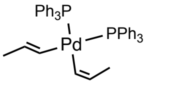<br>–2434.27918 **0.0** **0.0** Gaussian 16<br>(*G*° –2432.48144 **0.0** Gaussian 16)<br>–2432.65148 **0.0** M06/6-31G(d)<br>–2435.08388 **0.0** ωB97X-D<br>–2435.78336 **0.0** B3LYP-D3	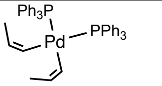<br>–2434.28132 **–1.3** **–1.4** Gauss<br>(*G*° –2432.48217 **–0.5** Gauss)<br>–2432.65289 **–0.9** M06/6-31G(d)<br>–2435.08594 **–1.3** ωB97X-D<br>–2435.78524 **–1.2** B3LYP-D3

The relative energies between *E* and *Z* organometallic compounds or complexes, among *EE*, *ZE*, and *ZZ* diorganometallic intermediates, and/or between *trans* and *cis* square planar intermediates, are given **in kcal/mol, in bold red**. In spite of differences among Δ*E* values obtained by the various methods, the results essentially agree: (i) the energies of the *Z* isomers of MeCH=CHMX are predicted to be slightly lower than (or, at high levels of theory, similar to) the corresponding *E* isomers; (ii) there are no differences among RCH=CHMCl, RCH=CHMBr, and RCH=CHMI species; (iii) the effect of solvents (implicit-solvent model) and the inclusion of entropy do not significantly change the relative energies of *Z* and *E* isomers; (iv) the *Z* isomers of R_LCH=CHMX, where R_L = large group, are much more stable than the corresponding *E* isomers; (v) usually the *ZE* intermediates of $(MeCH=CH)_2M$ have energies close to, or are slightly less stable than, the *EE* isomers, but this is reasonable in the light of the results with alkenide ions, since dialkenylmetals have less ionic M–X bonds (that is, as they contain less polar C–M–C bonds, as they have very low dipole moments). The cases in which the *Z* isomers are sometimes predicted to be slightly less stable than the *E* isomers are highlighted by means of an exclamation mark (**!**).

Obviously, the results are in sharp contrast with the relative energies between *Z*- and *E*-MeCH=CHMe (1.1–1.4 kcal/mol in favor of the *E* isomer, with M06-2X, MP2, and CCSD methods) and ^{t}BuCH=CHMe (3.9–4.3 kcal/mol in favor of the *E* isomer, *idem*).

Many results shown in Table S1 may be accounted for by dipole–dipole and/or dipole–induced dipole intramolecular interactions (vdW forces) involving polar bonds/groups or by simple electrostatic interactions in the case of the more ionic compounds.

The comparisons of the different methods may throw light on their relative performance regarding these organometallics.

M06-2X, B3LYP-D3, MP2, and CCSD(T) Energies of Alkenide Ions (Alkenyl Anions)

The M06-2X total energies and Gibbs free energies (free enthalpies), in au, of the two isomers of the 1-propenyl anion and of the two isomers of the 2-phenylethenyl anion are reproduced in the top of Table S2. The relative values are in kcal/mol, as always in bold. Additional calculations with other methods and basis sets, included in the last rows of Table S2, confirm that the anions in which the negative charge is *cis* to the methyl or phenyl groups are expected to be intrinsically more stable than the corresponding *trans* anions (see Ref 14 of the main text).

Table S2

	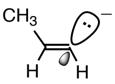	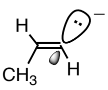	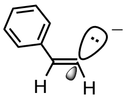	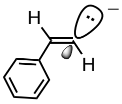
E, M06-2X/6-311+G(d,p)	–117.21154 **0.0**	–117.20888 **1.7**	–308.94218 **0.0**	–308.93808 **2.6**
G°, M06-2X/6-311+G(d,p)	–117.17154 **0.0**	–117.16893 **1.3**	–308.85339 **0.0**	–308.84923 **2.6**
E, M06-2X/6-311+G(d,p)·THF	–117.30442 **0.0**	–117.30299 **0.9**	–309.02609 **0.0**	–309.02453 **1.0**
E, M06-2X/6-311+G(d,p)·DMF	–117.31700 **0.0**	–117.31572 **0.8**	–309.03788 **0.0**	–309.03668 **0.8**
G°, M06-2X/6-311+G(d,p)·DMF	–117.27625 **0.0**	–117.27484 **0.9**	–308.94808 **0.0**	–308.94658 **0.9**
E, B3LYP-D3/6-311+G(d,p)	–117.28646 **0.0**	–117.28399 **1.5**	–309.09665 **0.0**	–309.09347 **2.0**
E, B3LYP-D3/cc-pVTZ	–117.28674 **0.0**	–117.28432 **1.5**	–309.11847 **0.0**	–309.11474 **2.3**
E, B3LYP-D3/def2-TZVP	–117.29190 **0.0**	–117.28994 **1.2**	–309.12682 **0.0**	–309.12379 **1.6**
E, B3LYP-D3/def2-TZVPPD	–117.29900 **0.0**	–117.29668 **1.5**	–309.13343 **0.0**	–309.13040 **1.9**
E, MP2/6-311+G(d,p)	–116.88439 **0.0**	–116.88116 **2.0**	–308.11850 **0.0**	–308.11520 **2.1**
E, CCSD(T)/6-311+G(d,p)// M06-2X/6-311+G(d,p)	–116.93856 **0.0**	–116.93558 **1.9**	–308.21889 **0.0**	–308.21511 **2.4**
E, CCSD(T)/6-311+G(d,p)// MP2/6-311+G(d,p)	–116.93893 **0.0**	–116.93595 **1.9**	–308.22008 **0.0**	–308.21691 **2.0**

Calculation of Plausible Schlenk-Type Equilibria

The following DFT calculations (Scheme S1) at the M06/6-311+G(d,p) level suggest that the formation in situ of dialkenylzinc intermediates is plausible, although partially (as the thought Schlenk-type equilibria, with the chosen models, are not sufficiently shifted towards the dipropen-1-ylzinc complexes). As mentioned above, during these exchanges, some *E*-alkenyl and *EE*-dialkenyl intermediates may partially be converted to the *Z* and *ZE* intermediates, respectively, which are thermodynamically more stable.

Scheme S1

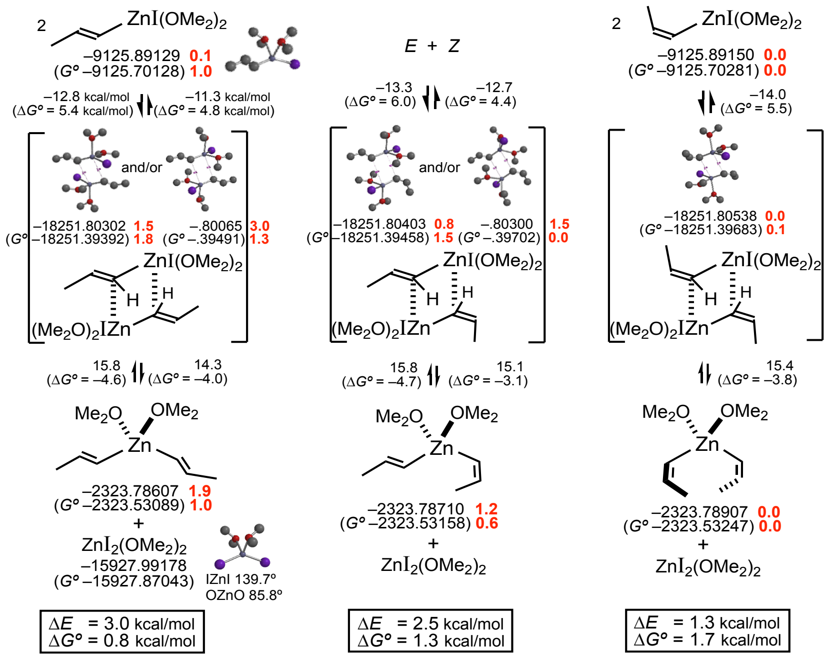

Calculated Energies of Dialkenylpalladium(0) Reaction Intermediates

The dialkenylpalladium intermediates will obviously be formed during the standard Negishi reaction. The well-known addition of RCH=CH–ZnX to RCH=CH–PdXL2 to give the species drawn below does not need comments or calculations.

However, it is interesting to compare the relative stability of all these possible species, when the phosphine/phosphane is a small ligand (e.g., PMe_3) and when the phosphane is PPh_3. In this last case, M06 predicts that the *cis* isomers (of the organometallic complexes) are always more stable than their corresponding *trans* isomers, as shown in the last rows of Table S1.

The fact that for L = PPh_3 the *cis* isomers of these reaction intermediates are thermodynamically favored is crucial for the C–C coupling, as it is thought from the beginning of organometallic chemistry. Known attractive non-covalent interactions (CH–π) among suitably oriented Ph groups of the two PPh_3 ligands are apparent (and, although at the available levels of theory the free energy gaps are calculated to be lower than the *E* gaps, they are still favorable to the *cis* isomers). London dispersion forces may also be invoked.[S14]

Among the *trans* isomers, that of *ZZ* configuration is predicted to be more stable than that of *ZE* configuration, and this more stable than that of *EE* configuration (in agreement with the results shown throughout this work). The same happens with the *cis* isomers, as expected.

Scheme S2

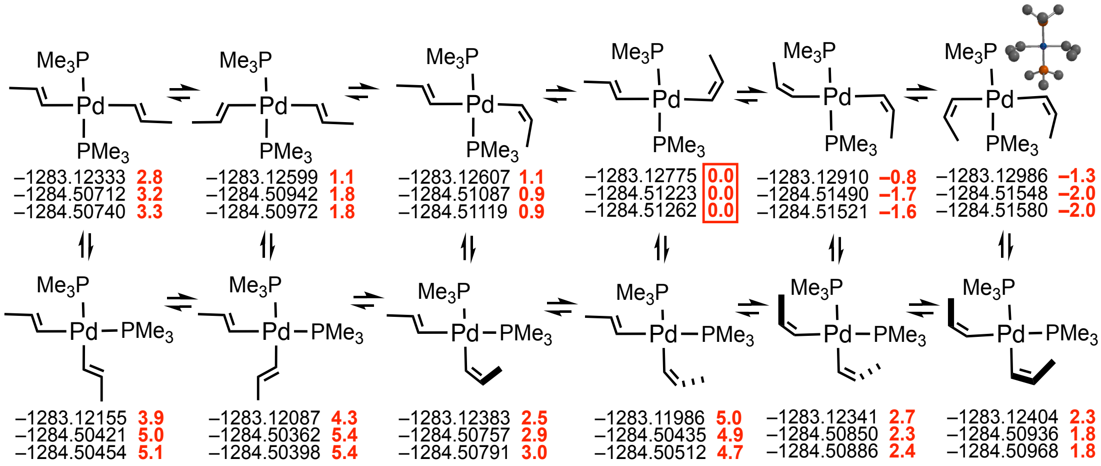

[First row, M06/6-31G(d). Second row, M06/6-311+G(d,p)//M06/6-31G(d). Third row, M06/6-311+G(d,p). For Pd, LANL2DZ. Relative energies (kcal/mol) in bold red.]

Scheme S3

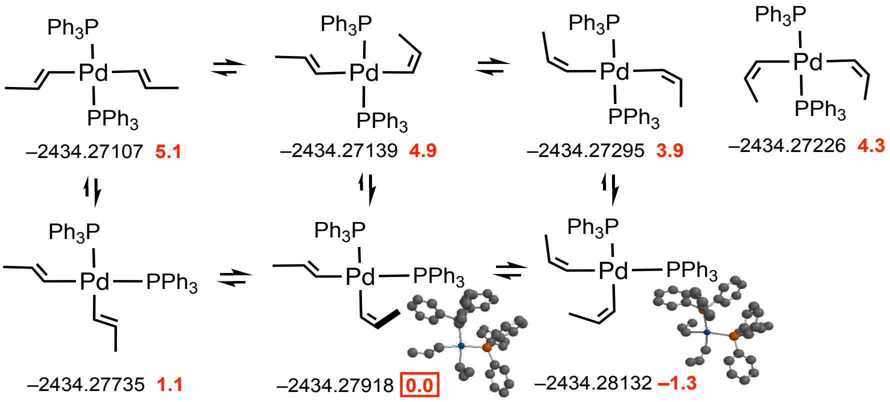

[M06/6-311+G(d,p)//M06/6-31G(d), with LANL2DZ(Pd). Relative energies (kcal/mol) in red bold.]

Scheme S4

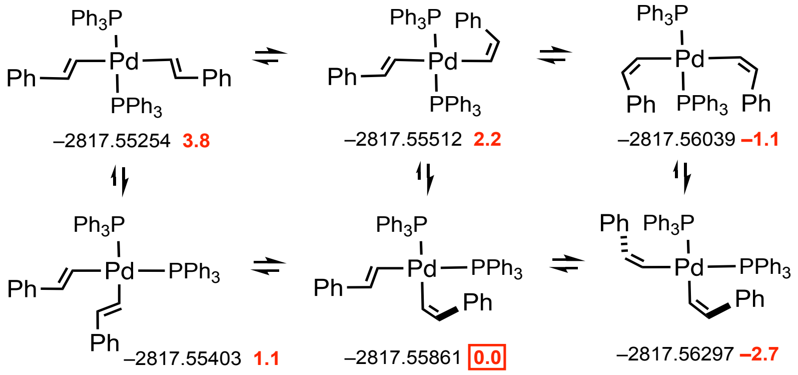

[M06/6-311+G(d,p)//M06/6-31G(d), with LANL2DZ(Pd). Relative energies in red bold (in kcal/mol)]

What is important in the present context is that the **isomerization of *EE* intermediates to** the corresponding ***ZE* intermediates** is feasible. Isomerization of *ZE* to *ZZ* intermediates is feasible as well, but the fact that we did not detect significant percentages of dimers with *ZZ* configuration indicates that the *E*-to-*Z* isomerization of organometallic intermediates is an equilibrium not completely shifted to the right (of course) and/or that lifetimes of Pd-coordinated *cis-EE* and *cis-ZE* species are relatively short (that their C–C coupling rates may be larger than those of stereoinversions), and/or that part of the final products of *ZZ* configuration revert in situ to their more stable partners.

Equilibrium Geometries (Cartesian Coordinates)

M06/6-311+G(d,p)

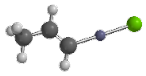

MeCH=CHZnCl, *E* –2356.84115 au

1 H H1	0.6186820	2.0240743	-0.3916051
2 C C1	0.7294442	1.4614181	0.5397359
3 H H2	1.7934968	1.4329860	0.8013277
4 H H4	0.2266766	2.0164541	1.3403706
5 C C2	0.1722521	0.0841650	0.4110905
6 H H5	0.2508730	-0.5311967	1.3098031
7 C C3	-0.3907279	-0.4114201	-0.6930889
8 H H6	-0.4462206	0.2425184	-1.5664968
9 Zn Zn1	-1.0912752	-2.1794647	-0.8167011
10 Cl Cl1	-1.8632010	-4.1395345	-0.9344358

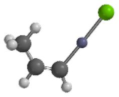

MeCH=CHZnCl, *Z* –2356.84223 au

1 H H1	0.2673417	-1.0598376	0.9375568
2 C C1	0.5081992	-0.0314013	1.2361802
3 H H2	-0.0393182	0.1863767	2.1604655
4 H H4	1.5738605	0.0036168	1.4896372
5 C C2	0.1753578	0.9505338	0.1671803
6 H H5	0.3965379	1.9927991	0.4159723
7 C C3	-0.3521172	0.6731294	-1.0253450
8 H H6	-0.5430153	1.4986662	-1.7090024
9 Zn Zn1	-0.7718128	-1.1130786	-1.5597886
10 Cl Cl1	-1.2150335	-3.1008044	-2.1128565

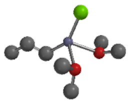

MeCH=CHZnCl·2OMe₂, *E* –2666.81521 au

conformer *syn/s-cis*

1 H H1	1.8573712	3.7643885	-0.6283753
2 C C1	2.0518194	3.6285810	0.4402067
3 H H2	3.1311899	3.7297152	0.6068050
4 H H4	1.5777746	4.4557944	0.9824334
5 C C2	1.5497320	2.3045736	0.9172678
6 H H5	1.7139427	2.1077263	1.9800815
7 C C3	0.9438156	1.3815864	0.1648088
8 H H6	0.8173354	1.6463006	-0.8937996
9 Zn Zn1	0.3003615	-0.3406452	0.8117097
10 O O1	-1.8762778	-0.4144303	0.2691025
11 O O2	0.5170984	-1.7520800	-0.9017001
12 C C4	-2.6774909	-0.0206663	1.3690304
13 H H7	-3.7397625	-0.1590483	1.1325031
14 H H8	-2.4948449	1.0350314	1.6167772
15 C C5	-2.1609124	0.3287177	-0.8996099
16 H H3	-3.2088166	0.1874731	-1.1936395
17 H H11	-1.5034122	-0.0396768	-1.6895058
18 H H12	-1.9675741	1.3978349	-0.7366515
19 C C7	1.8925366	-1.9552410	-1.1674362
20 H H9	2.3627491	-0.9729875	-1.2450077
21 H H16	2.0210600	-2.5006044	-2.1107085
22 H H17	2.3629333	-2.5234314	-0.3526973
23 H H18	-2.3986564	-0.6487927	2.2164345
24 C C6	-0.1935002	-2.9757341	-0.8005185
25 H H10	-1.2326043	-2.7303498	-0.5775942
26 H H13	0.2101761	-3.5880314	0.0151930
27 H H14	-0.1275646	-3.5235481	-1.7494008
28 Cl Cl1	0.2715214	-1.8224559	2.4242913

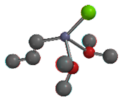

MeCH=CHZnCl·2OMe₂, *E* –2666.81672 au

conformer *anti/s-trans*

1 H H1	3.9012001	-1.8758797	-0.3221692
2 C C1	3.2695180	-1.5267598	-1.1449381
3 H H2	3.8053900	-0.7270209	-1.6714462
4 H H4	3.1600347	-2.3491606	-1.8628786
5 C C2	1.9406528	-1.0588266	-0.6436145
6 H H5	1.2606837	-0.7004937	-1.4248301
7 C C3	1.5426233	-1.0545356	0.6317123
8 H H6	2.2735624	-1.4357059	1.3524148
9 Zn Zn1	-0.2168799	-0.4641953	1.2380954
10 O O1	-0.3734792	1.7326611	0.7698272
11 O O2	-1.5831490	-0.6762332	-0.5053851
12 C C4	0.0434388	2.4940980	1.8890166
13 H H7	-0.1462803	3.5603691	1.7136499
14 H H8	1.1164996	2.3436731	2.0773727
15 C C5	0.2869113	2.1184916	-0.4197440
16 H H3	0.1031876	3.1808530	-0.6258497
17 H H11	-0.1188640	1.5105643	-1.2308472
18 H H12	1.3677567	1.9397692	-0.3419619
19 C C7	-1.7610972	-2.0495247	-0.8040946
20 H H9	-0.7707017	-2.5059502	-0.8572756
21 H H1	-2.2746571	-2.1643729	-1.7668903
22 H H17	-2.3499334	-2.5408246	-0.0171131
23 H H18	-0.5332470	2.1508474	2.7494507
24 C C6	-2.8199161	0.0143953	-0.4188595
25 H H10	-2.5960335	1.0516817	-0.1662902
26 H H13	-3.4455134	-0.4160543	0.3724332
27 H H14	-3.3421392	-0.0391277	-1.3826324
28 Cl Cl1	-1.7395681	-0.5127383	2.8128474

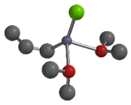

MeCH=CHZnCl·2OMe₂, *Z* –2666.81753 au

conformer *syn*

1 C C2	-1.4073409	-0.6580885	-2.5227183
2 C C3	-1.2150536	0.1437163	-1.4723280
3 H H6	-1.4068296	1.2080909	-1.6390121
4 Zn Zn1	-0.5663278	-0.4722086	0.2659934
5 O O1	1.6422967	-0.0902296	0.1705611
6 O O2	-0.5821713	1.2750441	1.6273218
7 C C4	2.2955362	-1.2475254	-0.3201923
8 H H7	3.3835079	-1.1137616	-0.2829417
9 H H8	1.9906191	-1.4485988	-1.3574918
10 C C5	1.9582395	1.0653263	-0.5815756
11 H H3	3.0380527	1.2604099	-0.5386167
12 H H11	1.4140111	1.9025633	-0.1391221
13 H H12	1.6463890	0.9454230	-1.6284314
14 C C7	-1.9197892	1.6275015	1.9274901
15 H H9	-2.4595531	1.7126047	0.9819746
16 H H16	-1.9505379	2.5899234	2.4537585
17 H H17	-2.3915739	0.8551904	2.5508773
18 H H18	2.0019445	-2.0829407	0.3172358
19 C C6	0.2200109	1.1657727	2.7938457
20 H H10	1.2200518	0.8717717	2.4732263
21 H H13	-0.1794084	0.3948263	3.4640628
22 H H14	0.2556438	2.1338392	3.3112496
23 Cl Cl1	-0.5519005	-2.0534867	1.7857295
24 C C8	-1.2094904	-2.1378148	-2.5336598
25 H H5	-2.1340515	-2.6627432	-2.8050111
26 H H15	-0.8907253	-2.5100370	-1.5515453
27 H H19	-0.4568100	-2.4369978	-3.2744185
28 H H20	-1.7447397	-0.2375711	-3.4762618

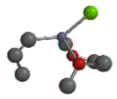

MeCH=CHZnCl·2OMe₂, *Z* –2666.81785 au

conformer *anti*

1 C C2	-0.9366505	-1.4968751	-2.3177144
2 C C3	0.3382961	-1.5370096	-1.9196558
3 H H6	1.0166723	-2.0439649	-2.6102444
4 Zn Zn1	1.1519607	-0.8407645	-0.2803443
5 O O1	1.2335857	1.3569412	-0.4073100
6 O O2	-0.4424844	-0.6141515	1.2981277
7 C C4	2.1284389	2.2110154	0.2780051
8 H H7	1.5819968	2.8616735	0.9759330
9 H H8	2.6757430	2.8380949	-0.4371253
10 C C5	0.2338020	2.0489907	-1.1246481
11 H H3	-0.4586284	2.5529237	-0.4344302
12 H H11	-0.3084136	1.3152543	-1.7239318
13 H H12	0.6858913	2.7976179	-1.7876860
14 C C7	-0.8060961	-1.9119391	1.7369385
15 H H9	-1.0223663	-2.5079249	0.8472659
16 H H16	-1.7002867	-1.8607885	2.3707305
17 H H17	0.0172021	-2.3744249	2.2973984
18 H H18	2.8249068	1.5774905	0.8299059
19 C C6	-0.1745888	0.2609791	2.3762621
20 H H10	0.0177549	1.2490391	1.9540106
21 H H13	0.7073401	-0.0710433	2.9391112
22 H H14	-1.0457860	0.3130754	3.0417622
23 Cl Cl1	2.7911683	-1.1152827	1.1636481
24 C C8	-2.0865141	-0.8829661	-1.5871579
25 H H5	-2.5777155	-0.1106091	-2.1943452
26 H H15	-1.7739782	-0.4320972	-0.6401649
27 H H19	-2.8612763	-1.6297976	-1.3687074
28 H H20	-1.2099742	-1.9534564	-3.2756337

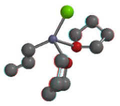

MeCH=CHZnCl·2THF, *E* –2821.58801 au

conformer *anti*

1 H H1	1.6429580	4.4529952	-0.1847989
2 C C1	1.4534314	3.8288572	-1.0637866
3 H H2	0.6168245	4.2685698	-1.6213308
4 H H4	2.3296595	3.8926976	-1.7210118
5 C C2	1.1684924	2.4145623	-0.6680630
6 H H5	0.9664413	1.7404705	-1.5081125
7 C C3	1.1581621	1.9398073	0.5808519
8 H H6	1.3870465	2.6748020	1.3601282
9 Zn Zn1	0.8166846	0.0704145	1.0492917
10 O O1	-1.3520333	-0.2940642	0.9300545
11 O O2	0.8927417	-1.0856753	-0.8124110
12 C C4	-2.0104121	-0.1645193	2.2073679
13 H H8	-1.3452742	0.3902851	2.8831865
14 C C5	-2.0506345	0.4766104	-0.0604439
15 H H11	-2.6734846	-0.2042652	-0.6581838
16 H H12	-1.3060614	0.9470227	-0.7090956
17 C C7	2.2542713	-1.2641807	-1.2402108
18 H H9	2.3235289	-1.0206102	-2.3100423
19 H H17	2.8780548	-0.5593038	-0.6815252
20 H H18	-2.1562438	-1.1650670	2.6220535
21 C C6	0.2146039	-2.3616796	-0.8162752
22 H H10	-0.6832011	-2.2688759	-1.4359127
23 H H13	-0.0821201	-2.5805692	0.2151555
24 Cl Cl1	1.3011293	-1.5547184	2.4553012
25 C C8	2.5525563	-2.7214069	-0.9726377
26 H H15	2.7596269	-2.8649738	0.0938521
27 H H16	3.3982483	-3.0943752	-1.5548515
28 C C9	1.2232884	-3.3662623	-1.3472859
29 H H20	1.1422100	-3.4618503	-2.4367046
30 H H21	1.0827283	-4.3564019	-0.9081126
31 C C10	-3.2896279	0.6008448	1.9279201
32 H H7	-4.0978856	-0.0858466	1.6499096
33 H H19	-3.6204073	1.1817234	2.7916637
34 C C11	-2.8898360	1.4541510	0.7303236
35 H H3	-3.7387092	1.8423185	0.1631680
36 H H22	-2.2667573	2.2985133	1.0505682

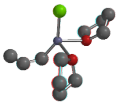

MeCH=CHZnCl·2THF, *Z* –2821.58878 au

conformer *syn*

1 C C2	-1.4130850	-0.8924918	-2.8111760
2 C C3	-1.3529192	-0.0562593	-1.7720142
3 H H6	-1.5965222	0.9893400	-1.9873603
4 Zn Zn1	-0.8121086	-0.5660398	0.0425423
5 O O1	1.3864564	-0.3966958	0.0725555
6 O O2	-0.8399857	1.2831345	1.2165766
7 C C4	2.0438651	-1.6577527	-0.1690319
8 H H8	1.3026438	-2.3628711	-0.5687096
9 C C5	1.9032547	0.6018072	-0.8214286
10 H H11	2.6325629	1.2171547	-0.2757951
11 H H12	1.0678378	1.2329374	-1.1377820
12 C C7	-2.1885127	1.7612064	1.3855220
13 H H9	-2.3981926	2.4801422	0.5886030
14 H H17	-2.8769492	0.9098908	1.2832123
15 H H18	2.4003615	-2.0487690	0.7872959
16 C C6	-0.1562524	1.2858904	2.4862607
17 H H10	0.5446231	2.1318901	2.4926984
18 H H13	0.4004879	0.3503439	2.5730229

19 Cl Cl1	-1.0331229	-2.0013122	1.6969330
20 C C8	-1.1292997	-2.3582861	-2.7611068
21 H H5	-1.9726920	-2.9447579	-3.1449834
22 H H15	-0.9324392	-2.6920001	-1.7345971
23 H H19	-0.2621300	-2.6250317	-3.3810404
24 H H20	-1.6941176	-0.5172163	-3.8017975
25 C C1	-1.2484747	1.4456540	3.5204434
26 H H2	-1.6960452	0.4673821	3.7310384
27 H H4	-0.8829159	1.8736740	4.4566058
28 C C9	-2.2401577	2.3386405	2.7852860
29 H H16	-3.2482155	2.3147970	3.2049745
30 H H21	-1.8929686	3.3787747	2.7865124
31 C C10	3.1362563	-1.3618671	-1.1799894
32 H H1	4.0621161	-1.0650983	-0.6732329
33 H H14	3.3578255	-2.2252815	-1.8112940
34 C C11	2.5514663	-0.1788950	-1.9419634
35 H H22	3.2968235	0.4022908	-2.4897150
36 H H23	1.7805258	-0.5143250	-2.6470654

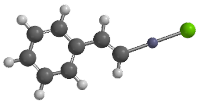

C_8H_7ZnCl –2548.47560 au

1 C C2	-0.5636288	-1.3670426	0.0582290
2 H H5	-0.7565861	-1.8436082	1.0236063
3 C C3	-0.8239215	-2.0353924	-1.0736366
4 H H6	-0.6129538	-1.5474733	-2.0266146
5 Zn Zn1	-1.5389956	-3.7994110	-1.1011190
6 Cl Cl1	-2.3396252	-5.7500853	-1.1267746
7 C C1	-0.0137470	-0.0132922	0.1839314
8 C C4	1.0478166	2.5555460	0.5332635
9 C C5	0.2570206	0.4859839	1.4597064
10 C C6	0.2547673	0.8060292	-0.9171377
11 C C7	0.7792086	2.0741205	-0.7440890
12 C C8	0.7840586	1.7564970	1.6361212
13 H H2	0.0497637	-0.1400324	2.3244651
14 H H1	0.0478866	0.4477647	-1.9211845
15 H H3	0.9809325	2.6962574	-1.6107320
16 H H4	0.9880343	2.1232673	2.6370984
17 H H7	1.4599691	3.5508713	0.6648675

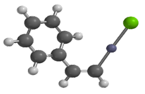

C_8H_7ZnCl –2548.61838 au

1 C C2	-1.2922206	1.3631919	-0.6476559
2 H H5	-1.4233199	2.4116849	-0.3767403
3 C C3	-2.1523336	0.7561378	-1.4751953
4 H H6	-2.9539391	1.3568208	-1.8964859
5 Zn Zn1	-1.9497337	-1.1676246	-1.7916413
6 Cl Cl1	-1.7731280	-3.2741285	-2.1762795
7 C C1	-0.1537968	0.6768627	0.0042730
8 C C4	1.8826964	-0.7404635	1.3128819
9 C C5	0.6269097	-0.2596254	-0.6845108
10 C C6	0.1381210	0.9250592	1.3493341
11 C C7	1.1418575	0.2192888	1.9989124
12 C C8	1.6288401	-0.9724544	-0.0334777
13 H H2	0.4970792	-0.3847209	-1.7568397
14 H H1	-0.4459601	1.6617416	1.8906282
15 H H3	1.3450163	0.4133379	3.0455421
16 H H4	2.2199853	-1.6937236	-0.5851086
17 H H7	2.6639263	-1.2913848	1.8223633

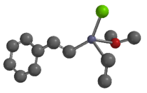

$C_8H_7ZnCl{\cdot}2OMe_2$ –2858.66124 au

1 C C2	0.9993374	1.2108355	0.9360981
2 H H5	1.3710742	0.8834288	1.9077860
3 C C3	0.1071728	0.4547619	0.2768564
4 H H6	-0.1899719	0.8385344	-0.7045564
5 Zn Zn1	-0.6252519	-1.2891852	0.9366616
6 O O1	-2.6078961	-1.5827966	0.1209217
7 O O2	-0.0295186	-2.7707229	-0.5485880
8 C C4	-3.7398635	-1.5853268	0.9849789
9 H H7	-4.5727805	-2.0985286	0.4964144
10 H H8	-4.0311895	-0.5591119	1.2294810
11 C C5	-2.8384636	-0.9164130	-1.1127318
12 H H3	-3.6732930	-1.3878095	-1.6390314
13 H H11	-1.9274579	-1.0125630	-1.7021175

14 H H12	-3.0573271	0.1420178	-0.9401370
15 C C7	1.3734757	-2.7776985	-0.7885356
16 H H9	1.6907571	-1.7386767	-0.8701812
17 H H16	1.5886468	-3.3108809	-1.7188188
18 H H17	1.8956494	-3.2602090	0.0439295
19 H H18	-3.4460042	-2.1156375	1.8888688
20 C C6	-0.5681870	-4.0807830	-0.3999620
21 H H10	-1.6334332	-3.9660507	-0.2099236
22 H H13	-0.1029664	-4.5862522	0.4503423
23 H H14	-0.4038778	-4.6481890	-1.3207052
24 Cl Cl1	-0.7745239	-2.6335352	2.7194817
25 C C1	1.5937500	2.4874614	0.4780636
26 C C8	2.8003643	4.8849077	-0.3595073
27 C C9	2.7757983	2.9454241	1.0692373
28 C C10	1.0166524	3.2687894	-0.5299272
29 C C11	1.6128912	4.4512089	-0.9452206
30 C C12	3.3784921	4.1273570	0.6528167
31 H H2	3.2286989	2.3589662	1.8624609
32 H H1	0.0810319	2.9550133	-0.9783840
33 H H4	1.1461572	5.0444746	-1.7235213
34 H H15	4.2985454	4.4578211	1.1213071
35 H H19	3.2635107	5.8093676	-0.6838572

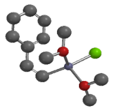

$C_8H_7ZnCl{\cdot}2OMe_2$ –2858.67075 au

1 C C2	-1.8023292	0.2220698	-1.7473071
2 C C3	-1.8838516	0.9594975	-0.6298763
3 H H6	-2.4506686	1.8859677	-0.7361347
4 Zn Zn1	-0.8207804	0.4529331	1.0083618
5 O O1	1.2204050	0.3518021	0.3297032
6 O O2	-0.2561537	2.2862537	2.0474169
7 C C4	1.8925310	-0.9053148	0.2652996
8 H H7	2.9696138	-0.7447715	0.3718526
9 H H8	1.6774849	-1.3979906	-0.6876977
10 C C5	1.5486096	1.2074047	-0.7582180
11 H H3	2.6291155	1.3791247	-0.7759302
12 H H11	1.0221527	2.1473990	-0.5984269
13 H H12	1.2218492	0.7592364	-1.7011984
14 C C7	-1.3989422	2.9115865	2.6198070
15 H H9	-2.1622599	2.9626825	1.8439574
16 H H16	-1.1433692	3.9213724	2.9528308
17 H H17	-1.7657743	2.3206059	3.4650566
18 H H18	1.5177809	-1.5085682	1.0906010
19 C C6	0.8238782	2.1867024	2.9725994
20 H H10	1.6301914	1.6635376	2.4632361
21 H H13	0.5160921	1.6118992	3.8496334
22 H H14	1.1444601	3.1909733	3.2644197
23 Cl Cl1	-0.7258127	-0.8894172	2.8072677
24 H H20	-2.2418518	0.5580038	-2.6890200
25 C C1	-1.0927300	-1.0783337	-1.8270568
26 C C8	0.3112298	-3.5113669	-1.9590825
27 C C9	-0.2939060	-1.3888173	-2.9351360
28 C C10	-1.1969089	-2.0267413	-0.8016799
29 C C11	-0.4975108	-3.2276450	-0.8631597
30 C C12	0.4070116	-2.5880426	-2.9985404
31 H H2	-0.2203625	-0.6746996	-3.7498860
32 H H1	-1.8603668	-1.8376758	0.0357469
33 H H5	-0.5963108	-3.9443023	-0.0558835
34 H H4	1.0257180	-2.8061895	-3.8617064
35 H H15	0.8517657	-4.4491759	-2.0118493

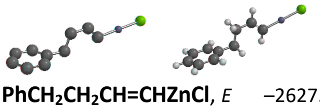

$PhCH_2CH_2CH{=}CHZnCl$, *E* –2627.05395 au

1 H H1	1.4152998	-0.8700135	-0.7969940
2 C C1	0.8305812	0.0000000	-1.1345757
3 H H4	1.4152998	0.8700135	-0.7969941
4 C C2	0.7929204	0.0000000	-2.6308323
5 H H5	1.7830084	0.0000000	-3.0920212
6 C C3	-0.2979091	0.0000000	-3.4015953
7 H H6	-1.2768674	0.0000000	-2.9212293
8 Zn Zn1	-0.2218343	-0.0000001	-5.3046718
9 Cl Cl1	-0.1156799	-0.0000001	-7.4109814
10 C C4	-0.5208890	0.0000000	-0.4327202
11 H H3	-1.0970044	-0.8809295	-0.7457027
12 H H7	-1.0970044	0.8809295	-0.7457027
13 C C5	-0.3642755	0.0000001	1.0595518
14 C C6	0.0098019	0.0000000	3.8336002

15 C C7	-0.2657153	-1.1968536	1.7647953
16 C C8	-0.2657153	1.1968536	1.7647953
17 C C9	-0.0813978	1.1997445	3.1408402
18 C C10	-0.0813978	-1.1997445	3.1408402
19 H H2	-0.3416796	-2.1386888	1.2247230
20 H H8	-0.3416796	2.1386888	1.2247231
21 H H9	-0.0132705	2.1426829	3.6750097
22 H H10	-0.0132705	-2.1426829	3.6750097
23 H H11	0.1486792	-0.0000001	4.9101324

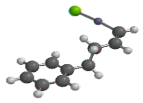

$PhCH_2CH_2CH{=}CHZnCl$, *Z* –2627.05541 au

1 H H1	-0.7549568	-0.8679293	-1.7233688
2 C C1	-0.4290919	0.1667372	-1.5490716
3 H H4	0.6732416	0.1382787	-1.5310737
4 C C2	-0.9045555	1.0660785	-2.6422583
5 H H5	-1.9550198	0.9427460	-2.9246133
6 C C3	-0.1701079	2.0094187	-3.2321801
7 H H6	-0.6285782	2.6399487	-3.9916694
8 Zn Zn1	1.6429045	2.2751882	-2.6827307
9 Cl Cl1	3.6138612	2.5167992	-1.9653677
10 C C4	-0.9269473	0.6181795	-0.1705345
11 H H3	-2.0247765	0.6224055	-0.1617259
12 H H7	-0.6077035	1.6569177	-0.0081580
13 C C5	-0.3950119	-0.2635279	0.9198108
14 C C6	0.6691049	-1.9639504	2.8673032
15 C C7	-1.1114683	-1.3724328	1.3619205
16 C C8	0.8644047	-0.0209660	1.4640772
17 C C9	1.3942371	-0.8624221	2.4314421
18 C C10	-0.5855440	-2.2175108	2.3298103
19 H H2	-2.0957995	-1.5711125	0.9426292
20 H H8	1.4347163	0.8422495	1.1239682
21 H H9	2.3754135	-0.6570796	2.8482285
22 H H10	-1.1587807	-3.0755835	2.6676361
23 H H11	1.0804580	-2.6224325	3.6259259

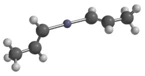

$C_6H_{10}Zn$, *EE* –2013.82212 au

1 C C2	2.7330325	0.2521659	0.2099852
2 C C3	1.8923606	-0.3417801	-0.6430746
3 H H6	2.3427692	-1.0509108	-1.3447553
4 Zn Zn1	0.0000000	0.0000000	-0.6667601
5 C C4	-1.8923606	0.3417801	-0.6430746
6 H H7	-2.3427692	1.0509108	-1.3447553
7 C C5	-2.7330325	-0.2521659	0.2099852
8 H H8	-2.3500747	-0.9716126	0.9394171
9 C C6	-4.2073457	-0.0264522	0.2749948
10 H H3	-4.5095769	0.3424700	1.2626051
11 H H9	-4.7596403	-0.9595041	0.1103343
12 H H10	-4.5306368	0.7005133	-0.4761267
13 C C7	4.2073457	0.0264522	0.2749948
14 H H5	4.5095769	-0.3424700	1.2626051
15 H H11	4.7596403	0.9595041	0.1103343
16 H H12	4.5306368	-0.7005133	-0.4761267
17 H H13	2.3500747	0.9716126	0.9394171

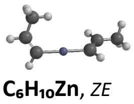

$C_6H_{10}Zn$, *ZE* –2013.82270 au

1 H H1	1.2258126	0.5372714	1.7985473
2 C C1	1.5108851	1.5122842	2.2127993
3 H H2	1.3871924	1.4635712	3.3013112
4 H H4	2.5825325	1.6552481	2.0288043
5 C C2	0.7054109	2.6199171	1.6221545
6 H H5	0.9503398	3.6144668	2.0093092
7 C C3	-0.2490174	2.4900904	0.6968380
8 H H6	-0.7399663	3.4093966	0.3722675
9 Zn Zn1	-0.7745786	0.7958985	-0.0653331
10 C C4	-1.2661966	-0.8993337	-0.8318591
11 H H7	-2.2699980	-1.3110544	-0.6860449
12 C C5	-0.4272663	-1.6473908	-1.5557848
13 H H8	0.5924820	-1.2975748	-1.7407177
14 C C6	-0.7487801	-2.9713217	-2.1665891
15 H H3	-0.0815601	-3.7558091	-1.7892708
16 H H9	-0.6169857	-2.9498051	-3.2551424
17 H H10	-1.7803060	-3.2658544	-1.9512894

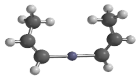

$C_6H_{10}Zn$, *ZZ*, ø 37.6 –2013.82388 au

1 H	H1	1.1569051	-0.6433224	0.9904865
2 C	C1	2.2272652	-0.4007808	0.9925138
3 H	H2	2.4092144	0.3091055	1.8085990
4 H	H4	2.7756948	-1.3152540	1.2481447
5 C	C2	2.6720288	0.1530090	-0.3193792
6 H	H5	3.7391764	0.3922749	-0.3722716
7 C	C3	1.8936450	0.3694623	-1.3828663
8 H	H6	2.3813787	0.7842441	-2.2667258
9 Zn	Zn1	0.0000000	0.0000000	-1.3970023
10 C	C4	-1.8936450	-0.3694623	-1.3828663
11 H	H7	-2.3813786	-0.7842441	-2.2667258
12 C	C5	-2.6720288	-0.1530090	-0.3193792
13 C	C7	-2.2272652	0.4007808	0.9925138
14 H	H8	-2.7756948	1.3152540	1.2481447
15 H	H11	-1.1569051	0.6433224	0.9904866
16 H	H12	-2.4092145	-0.3091055	1.8085991
17 H	H13	-3.7391764	-0.3922749	-0.3722716

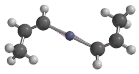

$C_6H_{10}Zn$, *ZZ, anti* –2013.82305 au

1 H	H1	1.4762631	0.0000000	-2.3256022
2 C	C1	0.8244670	0.0000000	-3.2081439
3 H	H2	1.0794678	0.8785594	-3.8129386
4 H	H4	1.0794679	-0.8785593	-3.8129386
5 C	C2	-0.6179417	0.0000000	-2.8288235
6 H	H5	-1.3098548	0.0000000	-3.6773776
7 C	C3	-1.1020234	0.0000000	-1.5843338
8 H	H6	-2.1893694	0.0000000	-1.4877065
9 Zn	Zn1	0.0000000	0.0000000	0.0000000
10 C	C4	1.1020235	0.0000000	1.5843338
11 H	H7	2.1893694	0.0000000	1.4877065
12 C	C5	0.6179417	0.0000000	2.8288235
13 C	C7	-0.8244670	0.0000000	3.2081439
14 H	H8	-1.0794679	-0.8785593	3.8129386
15 H	H11	-1.4762631	0.0000000	2.3256021
16 H	H12	-1.0794678	0.8785593	3.8129386
17 H	H13	1.3098548	0.0000000	3.6773776

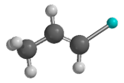

C_3H_5Li, *E* –124.76301 au

1 H	H1	-1.1068031	0.0000000	1.2951999
2 C	C1	-0.0168604	0.0000000	1.2216533
3 H	H2	0.3636306	0.8792282	1.7531365
4 H	H4	0.3636306	-0.8792282	1.7531365
5 C	C2	0.4174663	0.0000000	-0.2252712
6 H	H5	1.5068971	0.0000000	-0.3441678
7 C	C3	-0.3806609	0.0000000	-1.3081064
8 H	H6	-1.4424219	0.0000000	-1.0164306
9 Li	Li1	0.2951217	0.0000000	-3.1291500

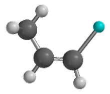

C_3H_5Li, *Z* –124.76371 au

1 H	H1	0.6655508	0.0000000	-1.4171583
2 C	C1	1.0628037	0.0000000	-0.3951458
3 H	H2	1.7076506	-0.8792078	-0.2887964
4 H	H4	1.7076506	0.8792078	-0.2887964
5 C	C2	-0.0509408	0.0000000	0.6223569
6 H	H5	0.3152831	0.0000000	1.6555528
7 C	C3	-1.3637651	0.0000000	0.3387877
8 H	H6	-1.9710262	0.0000000	1.2529322
9 Li	Li1	-2.0732067	0.0000000	-1.4797327

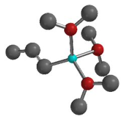

$C_3H_5Li·3OMe_2$ –589.83320 au

1 C	C2	-1.0626060	0.0295934	-2.5177155
2 C	C3	-1.2732032	0.6353931	-1.3335828
3 H	H6	-2.1830830	1.2632256	-1.3992072
4 Li	Li1	0.0416322	0.2885029	0.1941030
5 O	O1	1.8789372	0.0122308	-0.3435441
6 O	O2	-0.1063621	1.4073471	1.7698953
7 C	C4	2.8632777	-0.7661383	0.2984545
8 H	H7	3.6828256	-0.1354847	0.6622263
9 H	H8	3.2719418	-1.5151555	-0.3901660
10 C	C5	2.3430852	0.6542414	-1.5200517
11 H	H3	3.1461819	1.3586168	-1.2763493
12 H	H11	1.4884990	1.1753923	-1.9494491
13 H	H12	2.7158336	-0.0896632	-2.2326591
14 C	C7	-1.2083771	2.2952667	1.8404013
15 H	H9	-1.6423079	2.3332165	0.8429368
16 H	H16	-0.8744859	3.2909573	2.1520221
17 H	H17	-1.9504034	1.9224314	2.5565425
18 H	H18	2.3818289	-1.2699534	1.1370344
19 C	C6	0.5588226	1.2410773	3.0005860
20 H	H10	1.3523674	0.5102810	2.8469720
21 H	H13	-0.1298882	0.8702571	3.7696882
22 H	H14	0.9914341	2.1888345	3.3395608
23 O	O3	-0.2201794	-1.4988592	1.0536580
24 C	C1	-1.5040852	-1.4470860	1.6547098
25 H	H1	-1.4515509	-0.7313057	2.4743528
26 H	H4	-1.7795898	-2.4322267	2.0471966
27 H	H21	-2.2462073	-1.1116163	0.9220166
28 C	C9	-0.1886660	-2.4470902	-0.0053300
29 H	H2	0.8026104	-2.3975027	-0.4547380
30 H	H22	-0.9358756	-2.1894212	-0.7617200
31 H	H23	-0.3667393	-3.4541812	0.3876597
32 C	C10	-1.8886020	0.1398888	-3.7821362
33 H	H20	-1.2993590	0.5349104	-4.6183031
34 H	H24	-2.7402260	0.8061929	-3.6224784
35 H	H25	-2.2733129	-0.8352397	-4.1036489
36 H	H26	-0.1941673	-0.6369332	-2.6389371

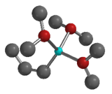

$C_3H_5Li·3OMe_2$ –589.83476 au

1 C	C2	-1.3032204	-0.0449304	-2.6693530
2 C	C3	-1.4934200	0.5115686	-1.4577383
3 H	H6	-2.4333851	1.0919677	-1.4688824
4 Li	Li1	-0.1651508	0.2611967	0.0920336
5 O	O1	1.7528241	0.2687254	-0.1854752
6 O	O2	-0.2868893	1.5818771	1.4934047
7 C	C4	2.6587197	-0.8018748	-0.0340524
8 H	H7	3.6595003	-0.4283561	0.2107329
9 H	H8	2.7143612	-1.3937236	-0.9557946
10 C	C5	2.1269909	1.1754083	-1.2116994
11 H	H3	3.0510433	1.6953353	-0.9359852
12 H	H11	1.3076062	1.8835357	-1.3274201
13 H	H12	2.2692476	0.6434646	-2.1578406
14 C	C7	-1.4654605	2.3552918	1.6170860
15 H	H9	-2.0019925	2.2556636	0.6750092
16 H	H16	-1.2136417	3.4052246	1.8019919
17 H	H17	-2.0844244	1.9842919	2.4431807
18 H	H18	2.2907431	-1.4249400	0.7812388
19 C	C6	0.5229536	1.5914277	2.6469016
20 H	H10	1.4051383	0.9931555	2.4226826
21 H	H13	-0.0101460	1.1579745	3.5021211
22 H	H14	0.8258425	2.6139960	2.8979346
23 C	C8	-0.1171938	-0.9111298	-3.0367915
24 H	H5	-0.4223151	-1.9243479	-3.3293550
25 H	H15	0.5700526	-0.9974637	-2.1885017
26 H	H19	0.4407658	-0.4980062	-3.8862381
27 H	H20	-2.0040400	0.0883904	-3.5039868
28 O	O3	-0.4748020	-1.4686490	1.0395245
29 C	C1	-1.7257049	-1.2670123	1.6775198
30 H	H1	-1.5800984	-0.5154490	2.4527773
31 H	H4	-2.0705792	-2.2002090	2.1359264
32 H	H21	-2.4636868	-0.9098502	0.9502668
33 C	C9	-0.5637583	-2.4994285	0.0640614
34 H	H2	0.4220701	-2.6111582	-0.3862955
35 H	H22	-1.2838694	-2.2208811	-0.7119266
36 H	H23	-0.8540810	-3.4410857	0.5429423

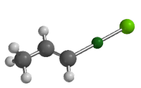

C_3H_5MgCl –777.60411 au

1 H	H1	0.8835681	0.0000000	2.0102549
2 C	C1	-0.1775939	0.0000000	1.7561567
3 H	H2	-0.6450264	-0.8789674	2.2100991
4 H	H4	-0.6450264	0.8789674	2.2100991
5 C	C2	-0.3755572	0.0000000	0.2657693
6 H	H5	-1.4227843	0.0000000	-0.0428414
7 C	C3	0.6016899	0.0000000	-0.6519373
8 H	H6	1.6149507	0.0000000	-0.2402566
9 Mg	Mg	0.2682847	0.0000000	-2.6772136
10 Cl	Cl1	-0.1025052	0.0000000	-4.8401302

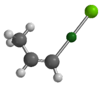

C_3H_5MgCl –777.60521 au

1 H	H1	-1.4147947	0.0000000	-0.1349788
2 C	C1	-1.0088089	0.0000000	0.8843263
3 H	H2	-1.4071759	0.8790714	1.3991473
4 H	H4	-1.4071759	-0.8790714	1.3991473
5 C	C2	0.4934540	0.0000000	0.8952125
6 H	H5	0.9279788	0.0000000	1.8980696
7 C	C3	1.2908104	0.0000000	-0.1796116
8 H	H6	2.3605569	0.0000000	0.0298465
9 Mg	Mg1	0.5093691	0.0000000	-2.0841673
10 Cl	Cl1	-0.3442139	0.0000000	-4.1069917

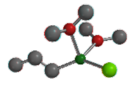

$C_3H_5MgCl·2OMe_2$ –1087.57732 au

1 H	H1	2.0810648	3.9918981	-0.7740450
2 C	C1	1.7877502	3.2484606	-1.5222192
3 H	H2	1.0370285	3.7062962	-2.1797592
4 H	H4	2.6613576	3.0382383	-2.1528913
5 C	C2	1.2657800	2.0066135	-0.8647991
6 H	H5	0.9676863	1.2246495	-1.5795554
7 C	C3	1.1436252	1.7838438	0.4517177
8 H	H6	1.4838593	2.6381658	1.0553667
9 Mg	Mg1	0.3743311	0.0094287	1.2735595
10 O	O1	-1.7101668	-0.0248529	0.9963611
11 O	O2	0.6668857	-1.4176011	-0.2860644
12 C	C4	-2.6600027	-0.7362203	1.7750823
13 H	H7	-3.1370566	-1.5173032	1.1676307
14 H	H8	-3.4296368	-0.0500462	2.1476073
15 C	C5	-2.2605092	0.5590538	-0.1730270
16 H	H3	-2.6295902	-0.2239864	-0.8492825
17 H	H11	-1.4656562	1.1312323	-0.6573165
18 H	H12	-3.0865513	1.2302504	0.0900006
19 C	C7	2.0451198	-1.6046314	-0.5838256
20 H	H9	2.5099924	-0.6161787	-0.5908222
21 H	H16	2.1544568	-2.0740770	-1.5683646
22 H	H17	2.5161367	-2.2353364	0.1816031
23 H	H18	-2.1217047	-1.1913626	2.6084683
24 C	C6	-0.0489041	-2.6403955	-0.2615890
25 H	H10	-1.0962881	-2.4065976	-0.0598352
26 H	H13	0.3322649	-3.2947966	0.5319714
27 H	H14	0.0337379	-3.1408754	-1.2340677
28 Cl	Cl1	0.5849895	-1.3938695	3.0580951

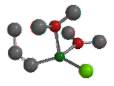

$C_3H_5MgCl·2OMe_2$ –1087.57765 au

1 C	C2	1.4234272	2.4096980	-0.8677799
2 C	C3	1.2874610	2.0619101	0.4202964
3 H	H6	1.6680536	2.8396211	1.0953906
4 Mg	Mg1	0.4924208	0.3065850	1.2705602
5 O	O1	-1.5982651	0.3095887	1.0310105
6 O	O2	0.8024994	-1.2539644	-0.1489702
7 C	C4	-2.5378366	-0.4363951	1.7903292
8 H	H7	-3.0138419	-1.1993096	1.1594871
9 H	H8	-3.3099360	0.2311845	2.1901957
10 C	C5	-2.1690864	0.9443959	-0.1023711
11 H	H3	-2.4755719	0.1931944	-0.8431881
12 H	H11	-1.4092896	1.6048790	-0.5265077
13 H	H12	-3.0415392	1.5371382	0.1966323
14 C	C7	2.1799950	-1.4600445	-0.4363383
15 H	H9	2.6538032	-0.4760633	-0.4817156
16 H	H16	2.2891568	-1.9666869	-1.4024603
17 H	H17	2.6458056	-2.0604879	0.3554661
18 H	H18	-1.9921098	-0.9119223	2.6076500
19 C	C6	0.0844971	-2.4710894	-0.0439022
20 H	H10	-0.9662529	-2.2213500	0.1180434
21 H	H13	0.4491027	-3.0624326	0.8049173

22 H	H14	0.1842647	-3.0440546	-0.9734629
23 Cl	Cl1	0.7132825	-0.9714389	3.1470660
24 C	C8	1.0197901	1.5908940	-2.0566793
25 H	H5	1.8799542	1.3744396	-2.7053430
26 H	H15	0.2964283	2.1252011	-2.6882295
27 H	H19	0.5747062	0.6321305	-1.7659018
28 H	H20	1.8690811	3.3743791	-1.1441949

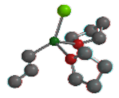

$C_3H_5MgCl{\cdot}2THF$ −1242.34938 au

1 C	C2	-0.9526835	0.2926083	-2.9086269
2 C	C3	-1.4564785	-0.7713073	-2.2677274
3 H	H6	-2.0376474	-1.4265571	-2.9332681
4 Mg	Mg1	-1.1029125	-1.1257990	-0.2319070
5 O	O1	0.8932464	-1.6731288	0.0541449
6 O	O2	-0.7595835	0.7882951	0.5934643
7 C	C4	1.9414669	-0.9258209	-0.6008582
8 H	H8	1.6809136	0.1370144	-0.5264718
9 C	C5	1.4488970	-2.4699399	1.1152733
10 H	H11	1.6929146	-3.4661812	0.7217713
11 H	H12	0.6793793	-2.5722129	1.8852151
12 C	C7	-0.2901971	0.9018670	1.9525925
13 H	H9	-0.1409652	-0.1070693	2.3496567
14 H	H17	0.6659418	1.4444902	1.9407768
15 H	H18	1.9613338	-1.2021542	-1.6588871
16 C	C6	-1.8383484	1.7286246	0.3679674
17 H	H10	-2.7762047	1.1625946	0.2818765
18 H	H13	-1.6511471	2.2372726	-0.5803996
19 Cl	Cl1	-2.0797695	-2.0572095	1.6237242
20 C	C1	-1.8467101	2.6250846	1.5876269
21 H	H2	-1.1357871	3.4508556	1.4647079
22 H	H4	-2.8344179	3.0505380	1.7773712
23 C	C9	-1.3722824	1.6716369	2.6760510
24 H	H16	-1.0034041	2.1748962	3.5723919
25 H	H21	-2.1756041	0.9823781	2.9618216
26 C	C10	3.2181013	-1.2645438	0.1550008
27 H	H1	3.7468082	-2.0904473	-0.3338451
28 H	H14	3.9019344	-0.4145877	0.2082189
29 C	C11	2.6944584	-1.7148007	1.5148158
30 H	H22	3.4041440	-2.3298357	2.0723447
31 H	H23	2.4253554	-0.8494571	2.1337763
32 C	C12	-1.1081117	0.6266862	-4.3620948
33 H	H3	-0.1372628	0.6849695	-4.8721719
34 H	H7	-1.5908845	1.6022619	-4.5073941
35 H	H20	-1.7132463	-0.1297429	-4.8728666
36 H	H24	-0.3512470	1.0287217	-2.3540718

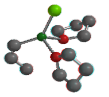

$C_3H_5MgCl{\cdot}2THF$ −1242.35018 au

1 C	C2	-1.0271469	0.2005396	-3.3503591
2 C	C3	-1.5470342	-0.7407928	-2.5503743
3 H	H6	-2.2374635	-1.4049009	-3.0866100
4 Mg	Mg1	-1.1720406	-1.0689389	-0.5046677
5 O	O1	0.8078748	-1.6237085	-0.1387608
6 O	O2	-0.8849035	0.7921571	0.4595227
7 C	C4	1.9108434	-0.8264798	-0.6177839
8 H	H8	1.7396287	0.2082962	-0.2917864
9 C	C5	1.2744319	-2.6008769	0.8122923
10 H	H11	1.4164569	-3.5568702	0.2919082
11 H	H12	0.4945918	-2.7239675	1.5683735
12 C	C7	-0.4766819	0.8341243	1.8432535
13 H	H9	-0.3400764	-0.1934306	2.1908594
14 H	H17	0.4756512	1.3797531	1.9027217
15 H	H18	1.9089301	-0.8507245	-1.7120080
16 C	C6	-1.9692371	1.7269978	0.2389918
17 H	H10	-2.8943663	1.1540117	0.0903550
18 H	H13	-1.7525908	2.2871163	-0.6738437
19 Cl	Cl1	-2.2283353	-2.1114342	1.2502357
20 C	C8	-0.0526832	1.2633700	-2.9388769
21 H	H5	0.8959376	1.1804775	-3.4884517
22 H	H15	0.1753577	1.2131392	-1.8664170
23 H	H19	-0.4364975	2.2702180	-3.1532813
24 H	H20	-1.2972528	0.2521888	-4.4137639
25 C	C1	-2.0356717	2.5619499	1.5001910
26 H	H2	-1.3293725	3.3994893	1.4460390
27 H	H4	-3.0344271	2.9680288	1.6707035
28 C	C9	-1.5947371	1.5595058	2.5584999

29 H	H16	-1.2660438	2.0203250	3.4930277
30 H	H21	-2.4022591	0.8517477	2.7767197
31 C	C10	3.1491078	-1.4283519	0.0213261
32 H	H1	3.5672826	-2.2140811	-0.6174746
33 H	H14	3.9279929	-0.6824627	0.1928102
34 C	C11	2.5886455	-2.0379476	1.3013060
35 H	H22	3.2351878	-2.7994951	1.7420212
36 H	H23	2.4109005	-1.2589729	2.0533012

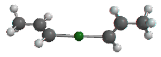

$C_6H_{10}Mg$ −434.52886 au

1 H	H1	4.6942718	-0.6979361	0.4693988
2 C	C1	4.3702827	0.0445400	-0.2665112
3 H	H2	4.9160600	0.9770671	-0.0763400
4 H	H4	4.6817097	-0.2992094	-1.2607665
5 C	C2	2.8910092	0.2554224	-0.2011475
6 H	H5	2.5198117	0.9932126	-0.9224443
7 C	C3	2.0375824	-0.3591014	0.6296041
8 H	H6	2.5246139	-1.0763146	1.3046918
9 Mg	Mg1	0.0000000	-0.0000002	0.6470298
10 C	C4	-2.0375824	0.3591013	0.6296041
11 H	H7	-2.5246138	1.0763145	1.3046917
12 C	C5	-2.8910093	-0.2554224	-0.2011475
13 H	H8	-2.5198117	-0.9932127	-0.9224443
14 C	C6	-4.3702827	-0.0445399	-0.2665112
15 H	H3	-4.9160601	-0.9770670	-0.0763400
16 H	H9	-4.6817097	0.2992095	-1.2607665
17 H	H10	-4.6942717	0.6979363	0.4693987

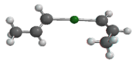

$C_6H_{10}Mg$ −434.52984 au

1 H	H1	1.6598892	3.9641445	0.3388864
2 C	C1	1.7123623	3.4079906	-0.6022774
3 H	H2	1.0641958	3.9077144	-1.3328303
4 H	H4	2.7353223	3.4862789	-0.9908673
5 C	C2	1.3117110	1.9804440	-0.4052903
6 H	H5	1.3505478	1.3817838	-1.3230928
7 C	C3	0.9304748	1.4143458	0.7481531
8 H	H6	0.9285298	2.1092660	1.5992315
9 Mg	Mg1	0.3872943	-0.5754251	0.9132778
10 C	C4	0.1746343	-2.5693572	1.0424952
11 H	H7	0.3630314	-3.3317493	1.6145526
12 C	C5	-1.2628190	-3.0167270	0.4047803
13 C	C7	-2.1730432	-2.1896116	-0.4453343
14 H	H8	-1.8534450	-1.1369771	-0.4907147
15 H	H11	-3.2029175	-2.2005862	-0.0680749
16 H	H12	-2.2149478	-2.5618329	-1.4763003
17 H	H13	-1.5615520	-4.0697016	0.4734055

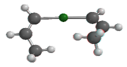

$C_6H_{10}Mg$ −434.53130 au

1 C	C2	2.7300013	0.1699686	0.2839458
2 C	C3	2.0196070	0.4766449	1.3765038
3 H	H6	2.5930684	0.9731944	2.1647977
4 Mg	Mg1	0.0000001	-0.0000005	1.4308712
5 C	C4	-2.0196070	-0.4766449	1.3765039
6 H	H7	-2.5930685	-0.9731944	2.1647977
7 C	C5	-2.7300013	-0.1699685	0.2839459
8 C	C7	-2.1958334	0.5089478	-0.9373235
9 H	H8	-1.1268042	0.7589950	-0.8411431
10 H	H11	-2.7263565	1.4454973	-1.1486771
11 H	H12	-2.3022354	-0.1174059	-1.8316322
12 H	H13	-3.7979702	-0.4122995	0.2180928
13 C	C6	2.1958335	-0.5089478	-0.9373235
14 H	H3	2.7263567	-1.4454972	-1.1486771
15 H	H5	2.3022353	0.1174059	-1.8316322
16 H	H9	1.1268043	-0.7589952	-0.8411430
17 H	H10	3.7979701	0.4123000	0.2180927

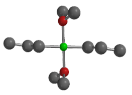

$C_6H_{10}Ni{\cdot}2OMe_2$, *EE trans* −2052.67515 au

1 C	C2	2.7041463	0.9723697	0.7912314
2 C	C3	1.7699824	0.8108171	-0.1491946
3 H	H6	1.9805170	1.3310114	-1.0989443
4 C	C4	3.9749984	1.7587230	0.6565799

5 H	H3	4.8639930	1.1379810	0.8284783
6 H	H5	4.0261023	2.5784038	1.3853601
7 H	H7	4.0567994	2.1953371	-0.3445629
8 H	H8	2.5649547	0.5100049	1.7781454
9 Ni	Ni1	-0.0056611	-0.0006771	-0.0042051
10 O	O1	0.6804966	-1.8302119	-0.0864339
11 O	O2	-0.6897896	1.8309156	0.0703224
12 C	C1	0.9362773	-2.2315922	-1.4220204
13 H	H4	0.0431653	-1.9956344	-2.0033473
14 H	H10	1.1180964	-3.3126806	-1.4461181
15 H	H11	1.8054717	-1.6944728	-1.8225672
16 C	C5	1.7078194	-2.2236347	0.8085772
17 H	H2	1.4238022	-1.8769811	1.8025773
18 H	H12	2.6645386	-1.7708817	0.5249149
19 H	H13	1.7791119	-3.3183832	0.8033754
20 C	C7	-1.7599438	2.2126882	-0.7786705
21 H	H9	-1.8341541	3.3071300	-0.7801494
22 H	H16	-2.7007191	1.7597982	-0.4476282
23 H	H17	-1.5219333	1.8580692	-1.7819517
24 C	C6	-0.8776050	2.2562486	1.4101511
25 H	H1	-1.0603749	3.3374922	1.4251122
26 H	H14	0.0450152	2.0314180	1.9476301
27 H	H15	-1.7252373	1.7257970	1.8631122
28 C	C8	-1.7801408	-0.8105232	0.1459423
29 H	H19	-2.0015996	-1.3118946	1.1039971
30 C	C9	-2.7033531	-0.9875131	-0.8022249
31 C	C11	-3.9772105	-1.7691493	-0.6700913
32 H	H18	-4.0143027	-2.6103596	-1.3749863
33 H	H23	-4.8614417	-1.1526160	-0.8793398
34 H	H24	-4.0811670	-2.1757177	0.3418523
35 H	H25	-2.5506543	-0.5412819	-1.7949235

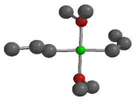

$C_6H_{10}Ni{\cdot}2OMe_2$, *ZE trans* −2052.67835 au

1 C	C2	2.5133351	0.7636859	0.8182214
2 C	C3	1.5609373	0.8615740	-0.1127862
3 H	H6	1.7945621	1.5292362	-0.9588821
4 C	C4	3.8454435	1.4552334	0.8074287
5 H	H3	4.6779001	0.7409167	0.8166964
6 H	H5	3.9801577	2.0964086	1.6881797
7 H	H7	3.9497096	2.0837031	-0.0837674
8 H	H8	2.3396623	0.1319101	1.6998147
9 Ni	Ni1	-0.2292854	0.0844423	0.0237040
10 O	O1	0.4221323	-1.7193852	-0.3422668
11 O	O2	-0.8942146	1.9108286	0.2619807
12 C	C1	1.3743353	-1.9156493	-1.3752181
13 H	H4	1.0594907	-1.3128166	-2.2272166
14 H	H10	1.3793089	-2.9790692	-1.6507172
15 H	H11	2.3716935	-1.5947518	-1.0537851
16 C	C5	0.7119868	-2.4769008	0.8217688
17 H	H2	-0.1406040	-2.3740271	1.4919580
18 H	H12	1.6267019	-2.1049996	1.3024206
19 H	H13	0.8413599	-3.5307709	0.5480718
20 C	C7	-2.0993360	2.2758098	-0.3970642
21 H	H9	-2.1915582	3.3674008	-0.3856662
22 H	H16	-2.9639426	1.8103436	0.0880458
23 H	H17	-2.0325993	1.9204257	-1.4248744
24 C	C6	-0.8585855	2.3372305	1.6154886
25 H	H1	-1.0455175	3.4164463	1.6622446
26 H	H14	0.1414396	2.1163174	1.9912968
27 H	H15	-1.6168667	1.8066784	2.2055229
28 C	C8	-2.0105746	-0.6700897	0.3692386
29 H	H19	-2.4173770	-0.5836479	1.3893934
30 C	C9	-2.7871701	-1.3283418	-0.4945211
31 C	C10	-2.4543159	-1.5468275	-1.9379605
32 H	H20	-1.5535572	-0.9872809	-2.2176089
33 H	H21	-3.2707011	-1.2232072	-2.5960516
34 H	H22	-2.2757839	-2.6078541	-2.1633706
35 H	H25	-3.7481668	-1.7529714	-0.1797185

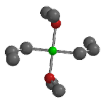

$C_6H_{10}Ni{\cdot}2OMe_2$, *ZZ trans* −2052.67998 au

1 C	C2	2.6296909	1.1919712	0.4943144
2 C	C3	1.7613616	0.7626471	-0.4242610
3 H	H6	2.0857484	0.8783570	-1.4698362
4 Ni	Ni1	0.0009787	0.0020017	-0.0001199

5	O	O1	0.5999103	-1.7954432	-0.4936678
6	O	O2	-0.5995143	1.8000558	0.4903011
7	C	C1	1.4475132	-1.9688662	-1.6178440
8	H	H4	1.0251059	-1.3914889	-2.4406904
9	H	H10	1.4665703	-3.0331035	-1.8823615
10	H	H11	2.4604267	-1.6105930	-1.4013481
11	C	C5	1.0684425	-2.5005098	0.6455508
12	H	H2	0.3024889	-2.4097479	1.4166156
13	H	H12	2.0159571	-2.0694730	0.9957580
14	H	H13	1.2107332	-3.5565813	0.3860503
15	C	C7	-1.0792671	2.4957605	-0.6503439
16	H	H9	-1.2250335	3.5527239	-0.3956423
17	H	H16	-2.0266082	2.0576881	-0.9922796
18	H	H17	-0.3177627	2.4048630	-1.4261237
19	C	C6	-1.4412917	1.9748451	1.6186641
20	H	H1	-1.4693145	3.0410227	1.8743200
21	H	H14	-1.0069126	1.4093800	2.4435210
22	H	H15	-2.4520551	1.6043609	1.4125683
23	C	C8	-1.7587060	-0.7585626	0.4251723
24	H	H19	-2.0829957	-0.8718528	1.4710463
25	C	C9	-2.6269535	-1.1912345	-0.4919070
26	C	C10	-2.3902071	-1.1386303	-1.9711300
27	H	H20	-1.4540541	-0.6123005	-2.1979147
28	H	H21	-3.2017862	-0.6212865	-2.4996421
29	H	H22	-2.3262717	-2.1436481	-2.4102066
30	H	H25	-3.5869961	-1.6334868	-0.1989959
31	C	C11	2.3924233	1.1348642	1.9733340
32	H	H8	3.2044077	0.6166553	2.5006625
33	H	H18	1.4567602	0.6064716	2.1981092
34	H	H23	2.3271177	2.1386238	2.4153613
35	H	H24	3.5900935	1.6345174	0.2029652

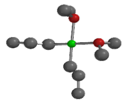

$C_6H_{10}Ni·2OMe_2$, *EE cis* −2052.70687 au

1	C	C2	-0.6345411	2.0635331	-1.6345239
2	C	C3	0.2762882	1.2258218	-1.1341361
3	H	H6	1.2955450	1.2856414	-1.5426125
4	C	C4	-0.3694375	3.1051788	-2.6782078
5	H	H3	-0.6256459	4.1143146	-2.3298530
6	H	H5	-0.9631091	2.9294352	-3.5847282
7	H	H7	0.6872556	3.1070787	-2.9665021
8	H	H8	-1.6735357	1.9932555	-1.2968849
9	Ni	Ni1	0.0000001	-0.0000003	0.2378018
10	O	O1	-0.3489106	-1.3455017	1.7946337
11	O	O2	0.3489106	1.3455012	1.7946336
12	C	C1	-1.4327823	-2.2389704	1.6169134
13	H	H4	-2.3038926	-1.6438694	1.3362382
14	H	H10	-1.6318611	-2.7708598	2.5564671
15	H	H11	-1.2163592	-2.9564325	0.8157845
16	C	C5	0.8406807	-2.0178471	2.1595749
17	H	H2	1.6054213	-1.2594853	2.3393595
18	H	H12	1.1681305	-2.6866113	1.3524605
19	H	H13	0.6782584	-2.5987901	3.0771141
20	C	C7	1.4327823	2.2389699	1.6169133
21	H	H9	1.6318610	2.7708593	2.5564670
22	H	H16	1.2163593	2.9564320	0.8157844
23	H	H17	2.3038926	1.6438689	1.3362382
24	C	C6	-0.8406807	2.0178467	2.1595745
25	H	H1	-0.6782587	2.5987897	3.0771137
26	H	H14	-1.6054214	1.2594849	2.3393589
27	H	H15	-1.1681304	2.6866108	1.3524600
28	C	C8	-0.2762874	-1.2258211	-1.1341350
29	H	H19	-1.2955440	-1.2856408	-1.5426117
30	C	C9	0.6345414	-2.0635327	-1.6345231
31	C	C11	0.3694371	-3.1051781	-2.6782071
32	H	H18	0.6256452	-4.1143142	-2.3298526
33	H	H23	0.9631086	-2.9294348	-3.5847276
34	H	H24	-0.6872561	-3.1070776	-2.9665013
35	H	H25	1.6735361	-1.9932557	-1.2968844

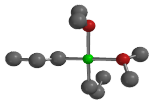

$C_6H_{10}Ni·2OMe_2$, *ZE cis* −2052.70955 au

1	C	C2	2.7535459	0.6898662	-0.3903002
2	C	C3	1.9231841	-0.3109255	-0.0900763
3	H	H6	2.3543165	-1.3121393	0.0285240
4	Ni	Ni1	0.0918744	-0.1513482	0.2116922
5	O	O1	-1.9411068	0.1600881	0.5673608
6	O	O2	0.2563176	0.1196634	2.2681324
7	C	C1	-2.6243206	-1.0515411	0.8205055
8	H	H4	-2.0765407	-1.5781498	1.6049663
9	H	H10	-2.6611572	-1.6718335	-0.0853088
10	H	H11	-3.6455537	-0.8417950	1.1651152
11	C	C5	-2.6097015	0.9608427	-0.3900663
12	H	H2	-2.0199516	1.8705346	-0.5212186
13	H	H12	-3.6123557	1.2163547	-0.0232703
14	H	H13	-2.6820866	0.4397273	-1.3526306
15	C	C7	1.1558906	-0.7430394	2.9388889
16	H	H9	1.0256878	-0.6434719	4.0243677
17	H	H16	2.1923709	-0.5125078	2.6618096
18	H	H17	0.9215273	-1.7635175	2.6299958
19	C	C6	0.4726932	1.4779397	2.5988439
20	H	H1	0.3621508	1.6236801	3.6815244
21	H	H14	-0.2806410	2.0674986	2.0721856
22	H	H15	1.4754070	1.7969105	2.2842230
23	C	C8	-0.0779671	-0.4685535	-1.6142575
24	H	H19	0.0913240	0.3796716	-2.2933829
25	C	C9	-0.4270270	-1.6321248	-2.1673449
26	C	C11	-0.6416239	-1.8615095	-3.6324009
27	H	H18	-1.6502333	-2.2373745	-3.8485889
28	H	H23	0.0595034	-2.6055920	-4.0324507
29	H	H24	-0.5002523	-0.9332963	-4.1963624
30	H	H25	-0.5485622	-2.5158188	-1.5328731
31	C	C10	2.3410859	2.1008488	-0.6695017
32	H	H8	2.8000214	2.8142212	0.0294347
33	H	H20	1.2513952	2.2065536	-0.5963892
34	H	H21	2.6435521	2.4178397	-1.6760857
35	H	H22	3.8272330	0.4922976	-0.4750613

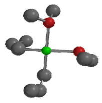

$C_6H_{10}Ni·2OMe_2$, *ZZ cis* −2052.71209 au

1	C	C2	1.9462596	0.8448360	1.9706740
2	C	C3	1.2536965	-0.1585399	1.4286711
3	H	H6	1.4609588	-1.1744405	1.7899819
4	Ni	Ni1	0.0002150	0.0004528	0.0584264
5	O	O1	-1.3560795	0.2947227	-1.4937869
6	O	O2	1.3558519	-0.2963230	-1.4950413
7	C	C1	-2.0159876	-0.9017091	-1.8568861
8	H	H4	-1.2499700	-1.6667087	-2.0039257
9	H	H10	-2.5740053	-0.7565011	-2.7912825
10	H	H11	-2.7018030	-1.2179766	-1.0602277
11	C	C5	-2.2560018	1.3732349	-1.3254226
12	H	H2	-1.6699624	2.2441723	-1.0236500
13	H	H12	-2.9917816	1.1486073	-0.5429082
14	H	H13	-2.7692100	1.5802869	-2.2737229
15	C	C7	2.0108193	0.9012174	-1.8639505
16	H	H9	2.5709535	0.7529795	-2.7966000
17	H	H16	2.6943204	1.2254367	-1.0683571
18	H	H17	1.2418923	1.6613803	-2.0166803
19	C	C6	2.2606396	-1.3707128	-1.3223861
20	H	H1	2.7743701	-1.5792996	-2.2695100
21	H	H14	1.6777073	-2.2428961	-1.0169548
22	H	H15	2.9946469	-1.1397551	-0.5408166
23	C	C8	-1.2558824	0.1581620	1.4268196
24	H	H19	-1.4641133	1.1732825	1.7879995
25	C	C9	-1.9476808	-0.8459985	1.9673306
26	C	C10	1.7424538	2.2959993	1.6708232
27	H	H8	2.6559956	2.7745918	1.2910818
28	H	H20	1.4517216	2.8560803	2.5688091
29	H	H21	0.9506164	2.4295703	0.9219952
30	H	H22	2.7121676	0.6267386	2.7224787
31	C	C4	-1.7399210	-2.2972640	1.6668935
32	H	H3	-0.9489539	-2.4291586	0.9181854
33	H	H5	-2.6523505	-2.7786898	1.2884919
34	H	H7	-1.4466394	-2.8560814	2.5649248
35	H	H25	-2.7149432	-0.6296968	2.7185224

M06/6-31G(d) geometries __________

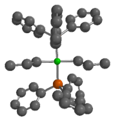

$C_{42}H_{40}NiP_2$, *EE trans syn* −3814.61738 au

M06/6-311+G(d,p)//M06/6-31G(d)

1	H	H1	-4.1441001	0.0983147	-1.8359856
2	C	C1	-3.4124455	0.0109106	-2.6512078
3	H	H2	-3.6911545	-0.8600344	-3.2638392
4	H	H4	-3.5281249	0.8974178	-3.2951156
5	C	C2	-2.0218935	-0.0975664	-2.1024943
6	H	H5	-1.2354601	-0.2093559	-2.8627664
7	C	C3	-1.6998966	-0.0361258	-0.8036138
8	H	H6	-2.5787410	0.0672820	-0.1418450
9	P	P1	-0.0039751	2.1368910	0.1009493
10	P	P2	-0.1257063	-2.1524921	0.2191370
11	C	C10	1.7275528	0.0054989	0.8769098
12	H	H25	1.8468444	-0.2554280	1.9432210
13	C	C11	2.8850454	0.1805436	0.2218442
14	H	H26	2.8734634	0.4283077	-0.8489674
15	C	C12	4.2536806	0.0486723	0.8166542
16	H	H24	4.8326480	0.9818396	0.7224131
17	H	H27	4.8465272	-0.7337505	0.3152115
18	H	H28	4.1929213	-0.2102161	1.8831041
19	C	C4	1.2256257	2.7015524	-1.1182168
20	C	C5	3.1817600	3.2113352	-3.0482128
21	C	C6	0.9985736	2.3755189	-2.4613364
22	C	C7	2.4480534	3.2652152	-0.7506784
23	C	C8	3.4189669	3.5226221	-1.7137455
24	C	C9	1.9694320	2.6363662	-3.4214160
25	H	H3	0.0565443	1.9036919	-2.7484784
26	H	H7	2.6523113	3.4858563	0.2963794
27	H	H8	4.3706948	3.9589394	-1.4150177
28	H	H9	1.7802361	2.3840169	-4.4634784
29	H	H10	3.9451625	3.4080131	-3.7990410
30	C	C13	0.4698985	-2.9563148	-1.3068733
31	C	C14	1.4602835	-4.1128663	-3.6464035
32	C	C15	0.6048861	-4.3453351	-1.4062326
33	C	C16	0.8515038	-2.1550986	-2.3834191
34	C	C17	1.3453143	-2.7306172	-3.5497385
35	C	C18	1.0910975	-4.9211387	-2.5732747
36	H	H11	0.3345181	-4.9754984	-0.5578299
37	H	H12	0.7598537	-1.0720931	-2.2951186
38	H	H13	1.6424629	-2.0965965	-4.3830311
39	H	H14	1.1898776	-6.0029912	-2.6443014
40	H	H15	1.8461070	-4.5638285	-4.5591615
41	C	C19	-1.5515700	3.0241334	-0.3342484
42	C	C20	-4.0175729	4.2370715	-0.8566411
43	C	C21	-1.6892170	3.9115376	-1.3997916
44	C	C22	-2.6597532	2.7648481	0.4794839
45	C	C23	-3.8840750	3.3635861	0.2205323
46	C	C24	-2.9186535	4.5131971	-1.6603292
47	H	H16	-0.8332083	4.1395995	-2.0336699
48	H	H17	-2.5585397	2.0784149	1.3228704
49	H	H18	-4.7395934	3.1460509	0.8576521
50	H	H19	-3.0132641	5.2035982	-2.4970522
51	H	H20	-4.9786645	4.7044950	-1.0640498
52	C	C25	0.3832167	2.9830079	1.6759443
53	C	C26	0.7980143	4.2720618	4.1196154
54	C	C27	0.5494403	4.3715914	1.7259180
55	C	C28	0.4069936	2.2525443	2.8643110
56	C	C29	0.6114755	2.8949330	4.0820057
57	C	C30	0.7660024	5.0112798	2.9394846
58	H	H21	0.5040645	4.9569850	0.8064307
59	H	H22	0.2628023	1.1728096	2.8276898
60	H	H23	0.6268712	2.3142773	5.0026279
61	H	H29	0.9013805	6.0911387	2.9668331
62	H	H30	0.9624185	4.7734365	5.0719610
63	C	C31	0.8850793	-3.0058160	1.4953397
64	C	C32	2.5662690	-4.1120048	3.4438203
65	C	C33	0.3571441	-3.5081610	2.6846964
66	C	C34	2.2697985	-3.0674765	1.2925211
67	C	C35	3.1023041	-3.6169303	2.2577787
68	C	C36	1.1940014	-4.0590008	3.6525365
69	H	H3	-0.7168491	-3.4888588	2.8593803
70	H	H32	2.6935158	-2.6731516	0.3702821
71	H	H34	4.1762561	-3.6561060	2.0833112
72	H	H35	0.7660221	-4.4524585	4.5729505
73	H	H36	3.2196513	-4.5410593	4.2017773
74	C	C37	-1.8189912	-2.7822770	0.5064802
75	C	C38	-4.4748899	-3.5254776	0.9792502
76	C	C39	-2.5178983	-2.2697863	1.6060087
77	C	C40	-2.4754760	-3.6497845	-0.3657147
78	C	C41	-3.7970448	-4.0181242	-0.1293983
79	C	C42	-3.8324562	-2.6468598	1.8482512
80	H	H33	-2.0318341	-1.5455401	2.2646751
81	H	H37	-1.9585209	-4.0281042	-1.2465022
82	H	H38	-4.2996655	-4.6910857	-0.8221508
83	H	H39	-4.3625609	-2.2418337	2.7085950
84	H	H40	-5.5085793	-3.8139897	1.1617289
85	Ni	Ni1	-0.0241932	-0.0081699	0.1118212

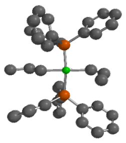

$C_{42}H_{40}NiP_2$, *ZE trans* –3814.62014 au

M06/6-311+G(d,p)//M06/6-31G(d)

1	H	H1	2.9466098	3.1250985	0.2536388
2	C	C1	1.9571600	3.5776907	0.1034666
3	H	H2	1.7595941	4.2439570	0.9583103
4	H	H4	2.0075243	4.2231494	-0.7880184
5	C	C2	0.9008753	2.5273890	-0.0404413
6	H	H5	-0.1048182	2.9228332	-0.2369453
7	C	C3	1.0935891	1.2048855	0.0461119
8	H	H6	2.1437929	0.9347388	0.2590099
9	P	P1	0.0060221	-0.1305173	-2.1662625
10	P	P2	-0.1917880	-0.1311200	2.1214243
11	C	C10	-1.3121906	-1.7722270	-0.1184143
12	H	H25	-2.3297307	-1.4970170	-0.4550879
13	C	C11	-1.1487604	-3.0814922	0.1092200
14	C	C4	-0.6535996	1.4952046	-2.6686260
15	C	C5	-1.7378242	4.0379329	-3.0969178
16	C	C6	0.1435003	2.5280611	-3.1615416
17	C	C7	-1.9974822	1.7542838	-2.3752156
18	C	C8	-2.5382892	3.0167258	-2.5907104
19	C	C9	-0.3987137	3.7904667	-3.3802383
20	H	H3	1.2016551	2.3515617	-3.3512786
21	H	H7	-2.6243296	0.9577468	-1.9671935
22	H	H8	-3.5859921	3.2042232	-2.3604795
23	H	H9	0.2352885	4.5891692	-3.7618856
24	H	H10	-2.1577296	5.0286111	-3.2614432
25	C	C13	-1.4192422	1.1453751	2.5790109
26	C	C14	-3.3552995	3.0539371	3.2183967
27	C	C15	-1.7411686	1.4049192	3.9149172
28	C	C16	-2.0853926	1.8427427	1.5701516
29	C	C17	-3.0507755	2.7925082	1.8874334
30	C	C18	-2.6993084	2.3595782	4.2326605
31	H	H11	-1.2412190	0.8489298	4.7091538
32	H	H12	-1.8356488	1.6374544	0.5285842
33	H	H13	-3.5630017	3.3281497	1.0897909
34	H	H14	-2.9410455	2.5589835	5.2752484
35	H	H15	-4.1098839	3.7977535	3.4689388
36	C	C19	1.6860896	-0.2852472	-2.8730055
37	C	C20	4.2340093	-0.7034848	-3.9407171
38	C	C21	1.9306840	-0.0599817	-4.2321001
39	C	C22	2.7253562	-0.7462053	-2.0629990
40	C	C23	3.9934710	-0.9562294	-2.5952199
41	C	C24	3.1999884	-0.2568037	-4.7604528
42	H	H16	1.1163489	0.2608299	-4.8832679
43	H	H17	2.5334300	-0.9440390	-1.0082574
44	H	H18	4.7954203	-1.3172575	-1.9540413
45	H	H19	3.3819481	-0.0705641	-5.8174071
46	H	H20	5.2274819	-0.8621450	-4.3569215
47	C	C25	-0.8816370	-1.3143118	-3.2554565
48	C	C26	-2.0936297	-3.2705389	-4.8479250
49	C	C27	-1.8612915	-0.9632744	-4.1837800
50	C	C28	-0.5050723	-2.6576506	-3.1444670
51	C	C29	-1.1040982	-3.6280178	-3.9343048
52	C	C30	-2.4676443	-1.9388668	-4.9732506
53	H	H21	-2.1540400	0.0788711	-4.3021553
54	H	H22	0.2686471	-2.9407562	-2.4303983
55	H	H23	-0.8012528	-4.6690354	-3.8354615
56	H	H29	-3.2326438	-1.6499029	-5.6923191
57	H	H30	-2.5684782	-4.0320794	-5.4643514
58	C	C31	-0.7317931	-1.5211304	3.1931066
59	C	C32	-1.6779104	-3.6959398	4.6828805
60	C	C33	0.0703043	-2.1165142	4.1675508
61	C	C34	-2.0267294	-2.0143462	2.9922520
62	C	C35	-2.4951315	-3.0926533	3.7300403
63	C	C36	-0.3991714	-3.2006797	4.9056209
64	H	H31	1.0711750	-1.7350768	4.3614886
65	H	H32	-2.6669879	-1.5550606	2.2412465
66	H	H34	-3.5020774	-3.4684889	3.5573522
67	H	H35	0.2416058	-3.6550587	5.6594505
68	H	H36	-2.0424758	-4.5465174	5.2565537
69	C	C37	1.3931958	0.3928600	2.8647350
70	C	C38	3.9443458	1.1745511	3.7044132
71	C	C39	2.4853374	-0.4714693	2.7239030
72	C	C40	1.5979002	1.6571018	3.4146052
73	C	C41	2.8677296	2.0446952	3.8326328
74	C	C42	3.7511256	-0.0876763	3.1487590
75	H	H33	2.3421513	-1.4505565	2.2608610
76	H	H37	0.7664440	2.3564044	3.4950460
77	H	H38	3.0155575	3.0380061	4.2535088
78	H	H39	4.5910326	-0.7711986	3.0360721
79	H	H40	4.9369480	1.4819926	4.0288709
80	Ni	Ni1	-0.1039862	-0.3077420	-0.0296302
81	C	C43	0.1295297	-3.7243529	0.5483991
82	H	H26	0.9147501	-2.9667008	0.6975824
83	H	H41	0.5082883	-4.4490173	-0.1911754
84	H	H42	0.0100154	-4.2815734	1.4919129
85	H	H43	-1.9866385	-3.7788538	-0.0245479

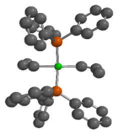

$C_{42}H_{40}NiP_2$, *ZZ trans anti* –3814.61685 au

M06/6-311+G(d,p)//M06/6-31G(d)

1	C	C2	0.3705317	2.9529586	-0.0634196
2	C	C3	0.7076988	1.6589614	0.0539498
3	H	H6	1.7804926	1.5148909	0.2516417
4	P	P1	0.0532819	0.0250786	-2.1336969
5	P	P2	-0.2186321	-0.0094920	2.1432479
6	C	C1	-1.6229569	-1.3704758	-0.1420534
7	H	H2	-2.6034491	-0.9334711	-0.4248348
8	C	C11	-1.6514583	-2.6961322	0.0342519
9	C	C4	-0.6615717	1.4737215	-2.9823016
10	C	C5	-1.9159173	3.7050829	-4.1068190
11	C	C6	0.0959406	2.5588773	-3.4233110
12	C	C7	-2.0567056	1.5332637	-3.0763215
13	C	C8	-2.6806217	2.6367868	-3.6453110
14	C	C9	-0.5305551	3.6671793	-3.9860274
15	H	H3	1.1791761	2.5435106	-3.3126406
16	H	H7	-2.6588875	0.7046179	-2.6980339
17	H	H8	-3.7661259	2.6663740	-3.7215134
18	H	H9	0.0702080	4.5087262	-4.3269991
19	H	H10	-2.4014946	4.5717015	-4.5520989
20	C	C13	-1.1062144	1.2941374	3.0681784
21	C	C14	-2.6236462	3.1732032	4.4701550
22	C	C15	-0.8887954	1.5010061	4.4348869
23	C	C16	-2.1073770	2.0163878	2.4218764
24	C	C17	-2.8645413	2.9536239	3.1192144
25	C	C18	-1.6357963	2.4438128	5.1291159
26	H	H11	-0.1327321	0.9147229	4.9589409
27	H	H12	-2.2867807	1.8446334	1.3609385
28	H	H13	-3.6425945	3.5124190	2.6015314
29	H	H14	-1.4535027	2.6050759	6.1902048
30	H	H15	-3.2107895	3.9099228	5.0158832
31	C	C19	1.8441711	-0.0001524	-2.5245884
32	C	C20	4.5727694	-0.2111197	-3.1026235
33	C	C21	2.3050226	0.1500470	-3.8372618
34	C	C22	2.7637288	-0.2880374	-1.5148732
35	C	C23	4.1207540	-0.3929596	-1.8006258
36	C	C24	3.6616779	0.0553931	-4.1220335
37	H	H16	1.5947410	0.3297109	-4.6455501
38	H	H17	2.4121160	-0.4300726	-0.4933739
39	H	H18	4.8228459	-0.6105309	-0.9970242
40	H	H19	4.0096978	0.1818738	-5.1457776
41	H	H20	5.6352953	-0.2851246	-3.3283347
42	C	C25	-0.4653007	-1.3943303	-3.1861958
43	C	C26	-1.0796338	-3.6889552	-4.6704315
44	C	C27	-1.2424087	-1.3040037	-4.3400548
45	C	C28	0.0204555	-2.6474348	-2.7990920
46	C	C29	-0.2834020	-3.7852508	-3.5311971
47	C	C30	-1.5518340	-2.4474838	-5.0749094
48	H	H21	-1.6108782	-0.3382485	-4.6818794
49	H	H22	0.6486210	-2.7288722	-1.9116024
50	H	H23	0.0997589	-4.7525433	-3.2090455
51	H	H29	-2.1633735	-2.3606827	-5.9717213
52	H	H30	-1.3253356	-4.5810530	-5.2441971
53	C	C31	-0.7770001	-1.4673485	3.1124090
54	C	C32	-1.8219356	-3.6421853	4.5296788
55	C	C33	0.0397726	-2.2211748	3.9559897
56	C	C34	-2.1328846	-1.7979346	3.0107217
57	C	C35	-2.6513278	-2.8773098	3.7123554
58	C	C36	-0.4793204	-3.3071263	4.6570304
59	H	H31	1.0906768	-1.9622687	4.0752281
60	H	H32	-2.7817320	-1.2076709	2.3652167
61	H	H34	-3.7068878	-3.1264883	3.6184214
62	H	H35	0.1714322	-3.8904610	5.3065137
63	H	H36	-2.2269032	-4.4935789	5.0742776
64	C	C37	1.5505284	0.1565154	2.5685546
65	C	C38	4.3269326	0.4365666	2.7972797
66	C	C39	2.4059167	-0.8908512	2.2049803
67	C	C40	2.1050604	1.3510033	3.0300085
68	C	C41	3.4847729	1.4862628	3.1483422
69	C	C42	3.7842178	-0.7552965	2.3249541
70	H	H33	1.9877233	-1.8190761	1.8107328
71	H	H37	1.4578736	2.1922449	3.2739404
72	H	H38	3.9029634	2.4251090	3.5072133
73	H	H39	4.4345515	-1.5817856	2.0417884
74	H	H40	5.4061564	0.5481109	2.8860362
75	Ni	Ni1	-0.3564005	0.0336556	-0.0198689
76	C	C43	-0.4907582	-3.5506644	0.4364316
77	H	H26	0.4389972	-2.9623399	0.4819959
78	H	H41	-0.3254493	-4.3788667	-0.2717571
79	H	H42	-0.6459908	-4.0076394	1.4279296
80	H	H43	-2.5913929	-3.2496429	-0.0933970
81	C	C12	-0.9927496	3.5063723	-0.3303663
82	H	H5	-0.9892249	4.1910507	-1.1924979
83	H	H24	-1.3751627	4.0810634	0.5289119
84	H	H27	-1.7158161	2.7087710	-0.5528522
85	H	H28	1.1476884	3.7217093	0.0475555

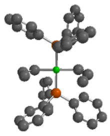

$C_{42}H_{40}NiP_2$, *ZZ trans syn* –3814.61854 au

M06/6-311+G(d,p)//M06/6-31G(d)

1	C	C2	-0.0637967	-2.7875024	0.8655044
2	C	C3	0.0421931	-1.9237117	-0.1526448
3	H	H6	0.1189168	-2.4127373	-1.1372292
4	P	P1	-2.1527291	0.0694683	-0.1843132
5	P	P2	2.1527291	-0.0694683	-0.1843131
6	C	C10	-0.0421931	1.9237115	-0.1526449
7	H	H25	-0.1189168	2.4127371	-1.1372293
8	C	C11	0.0637967	2.7875023	0.8655043
9	C	C4	-2.8642078	1.0172006	1.2057699
10	C	C5	-3.7466370	2.4697588	3.4287585
11	C	C6	-3.4277053	2.2834314	1.0442031
12	C	C7	-2.7184905	0.5006391	2.4974929
13	C	C8	-3.1684430	1.2161123	3.6012256
14	C	C9	-3.8677923	3.0036547	2.1497673
15	H	H3	-3.5115306	2.7172657	0.0488934
16	H	H7	-2.2401577	-0.4694631	2.6391584
17	H	H8	-3.0541785	0.7964812	4.5992185
18	H	H9	-4.3041034	3.9911998	2.0095027
19	H	H10	-4.0925027	3.0354265	4.2922248
20	C	C13	2.8277408	-0.8232155	-1.7145217
21	C	C14	3.8420351	-1.8055407	-4.1290177
22	C	C15	4.2049694	-1.0044241	-1.8853113
23	C	C16	1.9709067	-1.1217812	-2.7750620
24	C	C17	2.4745880	-1.6088893	-3.9775860
25	C	C18	4.7076509	-1.5016431	-3.0807559
26	H	H11	4.8889908	-0.7447889	-1.0762433
27	H	H12	0.8979994	-0.9688925	-2.6541973
28	H	H13	1.7943839	-1.8358669	-4.7965834
29	H	H14	5.7805054	-1.6436895	-3.1991561
30	H	H15	4.2372586	-2.1898330	-5.0678601
31	C	C19	-2.8277408	0.8232155	-1.7145217
32	C	C20	-3.8420351	1.8055408	-4.1290178
33	C	C21	-4.2049694	1.0044242	-1.8853113
34	C	C22	-1.9709067	1.1217812	-2.7750620
35	C	C23	-2.4745881	1.6088893	-3.9775860
36	C	C24	-4.7076510	1.5016432	-3.0807558
37	H	H16	-4.8889909	0.7447891	-1.0762433
38	H	H17	-0.8979994	0.9688925	-2.6541974
39	H	H18	-1.7943839	1.8358669	-4.7965834
40	H	H19	-5.7805054	1.6436897	-3.1991561
41	H	H20	-4.2372587	2.1898330	-5.0678601
42	C	C25	-3.1082622	-1.5039058	-0.2037477
43	C	C26	-4.4018118	-3.9813423	-0.3980684
44	C	C27	-3.9882011	-1.9228142	0.7925820
45	C	C28	-2.8991758	-2.3365270	-1.3097416
46	C	C29	-3.5400548	-3.5616617	-1.4096923
47	C	C30	-4.6264867	-3.1586113	0.6969063
48	H	H21	-4.1928929	-1.2847051	1.6505021
49	H	H22	-2.2213875	-2.0182052	-2.1035646
50	H	H23	-3.3628037	-4.1960481	-2.2765186
51	H	H29	-5.3083034	-3.4724840	1.4857915
52	H	H30	-4.9001684	-4.9465855	-0.4701250
53	C	C31	3.1082622	1.5039058	-0.2037477
54	C	C32	4.4018118	3.9813423	-0.3980683

55 C	C33	3.9882012	1.9228142	0.7925820
56 C	C34	2.8991758	2.3365270	-1.3097415
57 C	C35	3.5400548	3.5616617	-1.4096922
58 C	C36	4.6264867	3.1586113	0.6969063
59 H	H31	4.1928929	1.2847050	1.6505021
60 H	H32	2.2213873	2.0182053	-2.1035645
61 H	H34	3.3628037	4.1960481	-2.2765186
62 H	H35	5.3083036	3.4724838	1.4857915
63 H	H36	4.9001684	4.9465854	-0.4701249
64 C	C37	2.8642078	-1.0172006	1.2057700
65 C	C38	3.7466370	-2.4697588	3.4287585
66 C	C39	2.7184905	-0.5006392	2.4974929
67 C	C40	3.4277054	-2.2834314	1.0442031
68 C	C41	3.8677923	-3.0036547	2.1497673
69 C	C42	3.1684430	-1.2161123	3.6012256
70 H	H33	2.2401576	0.4694631	2.6391584
71 H	H37	3.5115307	-2.7172657	0.0488934
72 H	H38	4.3041035	-3.9911998	2.0095027
73 H	H39	3.0541783	-0.7964813	4.5992186
74 H	H40	4.0925029	-3.0354265	4.2922248
75 Ni	Ni1	0.0000000	-0.0000001	-0.1493659
76 C	C43	0.2097494	2.4110615	2.3062696
77 H	H26	0.1251726	1.3216055	2.4408026
78 H	H41	-0.5585036	2.8791294	2.9411733
79 H	H42	1.1865179	2.7300885	2.7085061
80 H	H43	0.0779905	3.8682467	0.6723695
81 C	C12	-0.2097493	-2.4110614	2.3062697
82 H	H5	0.5585036	-2.8791293	2.9411734
83 H	H24	-1.1865178	-2.7300882	2.7085063
84 H	H27	-0.1251725	-1.3216054	2.4408027
85 H	H28	-0.0779906	-3.8682467	0.6723698

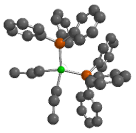

$C_{42}H_{40}NiP_2$, *EE cis* –3814.61654 au
M06/6-311+G(d,p)//M06/6-31G(d)

1 H	H1	2.2000780	-3.0451805	3.8877369
2 C	C1	2.6926182	-2.0643909	3.9491290
3 H	H2	2.7772653	-1.7935309	5.0127836
4 H	H4	3.7202148	-2.1919171	3.5720912
5 C	C2	1.9326915	-1.0471250	3.1540413
6 H	H5	2.3216751	-0.0210760	3.1970490
7 C	C3	0.8571783	-1.3252333	2.4046038
8 H	H6	0.4878197	-2.3588135	2.4681310
9 P	P1	1.1346922	-1.2127860	-0.4827315
10 P	P2	-1.1761118	1.3680933	0.1790544
11 C	C10	-0.9417117	-0.0007420	2.7558741
12 H	H25	-0.6369945	0.8618289	3.3680614
13 C	C11	-1.9925527	-0.7175161	3.1816621
14 H	H26	-2.3013006	-1.6075808	2.6165339
15 C	C12	-2.8086038	-0.4008525	4.3975731
16 H	H24	-2.8003126	-1.2255222	5.1267693
17 H	H27	-3.8675406	-0.2131863	4.1546336
18 H	H28	-2.4202598	0.4957350	4.9000170
19 C	C4	0.3606906	-1.4033930	-2.1456955
20 C	C5	-1.0149784	-1.5240498	-4.5926771
21 C	C6	0.2541882	-0.2739239	-2.9674315
22 C	C7	-0.2177976	-2.5983695	-2.5833366
23 C	C8	-0.9038957	-2.6562403	-3.7938600
24 C	C9	-0.4241719	-0.3337878	-4.1782386
25 H	H3	0.7167191	0.6643056	-2.6654058
26 H	H7	-0.1298343	-3.5017324	-1.9830914
27 H	H8	-1.3447667	-3.5984264	-4.1159207
28 H	H9	-0.4888781	0.5586619	-4.7993600
29 H	H10	-1.5521438	-1.5697477	-5.5383605
30 C	C13	-0.2603446	2.3852469	-1.0480266
31 C	C14	1.3243431	3.8016741	-2.8701841
32 C	C15	-0.8179698	2.8957397	-2.2221621
33 C	C16	1.0975667	2.6065944	-0.7931966
34 C	C17	1.8873682	3.3100188	-1.6956822
35 C	C18	-0.0282435	3.5972207	-3.1283666
36 H	H11	-1.8694733	2.7186183	-2.4468110
37 H	H12	1.5452860	2.1998119	0.1157805
38 H	H13	2.9446082	3.4620253	-1.4846505
39 H	H14	-0.4712303	3.9816281	-4.0459986
40 H	H15	1.9404003	4.3432632	-3.5858468
41 C	C19	2.7704294	-0.4801944	-0.8691933
42 C	C20	5.2906528	0.6422005	-1.3337961
43 C	C21	3.3803540	-0.6019903	-2.1203380
44 C	C22	3.4453900	0.1964179	0.1516267
45 C	C23	4.6987991	0.7535644	-0.0790570
46 C	C24	4.6308674	-0.0381881	-2.3523978
47 H	H16	2.8724576	-1.1382832	-2.9211913
48 H	H17	2.9851107	0.2707661	1.1370151
49 H	H18	5.2146627	1.2736869	0.7263245
50 H	H19	5.0927880	-0.1364121	-3.3334510
51 H	H20	6.2704670	1.0809375	-1.5155820
52 C	C25	1.5486257	-2.9408915	-0.0287800
53 C	C26	2.0763057	-5.5473493	0.8472616
54 C	C27	0.5037150	-3.7917930	0.3541421
55 C	C28	2.8594222	-3.4066599	0.0538725
56 C	C29	3.1206883	-4.7034637	0.4906881
57 C	C30	0.7630725	-5.0882749	0.7776966
58 H	H21	-0.5247482	-3.4212633	0.3524462
59 H	H22	3.6883375	-2.7524667	-0.2133982
60 H	H23	4.1503137	-5.0513582	0.5551999
61 H	H2	-0.0609830	-5.7358807	1.0718298
62 H	H30	2.2832418	-6.5595244	1.1901275
63 C	C31	-2.7050716	0.8618910	-0.6870664
64 C	C32	-4.9414891	0.0064953	-2.1334512
65 C	C33	-3.7489756	1.7500295	-0.9713307
66 C	C34	-2.8077910	-0.4616985	-1.1206005
67 C	C35	-3.9149845	-0.8863436	-1.8475910
68 C	C36	-4.8602083	1.3244344	-1.6892663
69 H	H31	-3.6923343	2.7808594	-0.6198096
70 H	H32	-2.0063412	-1.1644041	-0.8911274
71 H	H34	-3.9675628	-1.9181285	-2.1911270
72 H	H35	-5.6664776	2.0240806	-1.9031563
73 H	H36	-5.8109951	-0.3231539	-2.6998001
74 C	C37	-1.7705884	2.6556746	1.3425968
75 C	C38	-2.5776144	4.4783201	3.3098327
76 C	C39	-2.8864635	2.3897474	2.1449354
77 C	C40	-1.0637418	3.8425087	1.5429966
78 C	C41	-1.4662914	4.7488821	2.5198804
79 C	C42	-3.2881607	3.2966574	3.1177802
80 H	H33	-3.4352432	1.4585844	2.0135806
81 H	H37	-0.1937469	4.0705010	0.9285405
82 H	H38	-0.9071238	5.6724911	2.6601651
83 H	H39	-4.1562816	3.0701331	3.7352929
84 H	H40	-2.8913835	5.1866542	4.0747269
85 Ni	Ni1	-0.0273870	-0.2531379	1.1484615

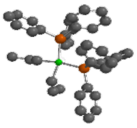

$C_{42}H_{40}NiP_2$, *ZE cis* –3814.61915 au
M06/6-311+G(d,p)//M06/6-31G(d)

1 H	H1	-3.9870269	-3.8501206	-0.0537581
2 C	C1	-3.7380117	-3.5464768	-1.0796703
3 H	H2	-3.2802717	-4.4119752	-1.5859514
4 H	H4	-4.6833100	-3.3446456	-1.6065670
5 C	C2	-2.8318043	-2.3544903	-1.0792275
6 H	H5	-2.5220269	-2.0032803	-2.0717876
7 C	C3	-2.4053760	-1.7127850	0.0164886
8 H	H6	-2.7937463	-2.0954735	0.9726869
9 P	P1	0.4286517	-1.5746895	-0.1114573
10 P	P2	-0.3244533	1.7827285	0.1320789
11 C	C10	-3.0110277	0.5439781	0.1934569
12 H	H25	-3.3425496	0.8739473	-0.8037522
13 C	C11	-3.8738362	0.7383739	1.1964070
14 C	C4	0.5083515	-2.9288717	1.1213529
15 C	C5	0.7223149	-4.8761929	3.1165627
16 C	C6	-0.3028690	-2.8738722	2.2555265
17 C	C7	1.4450707	-3.9624955	1.0073382
18 C	C8	1.5441682	-4.9356184	1.9932328
19 C	C9	-0.1955092	-3.8411851	3.2503710
20 H	H3	-1.0262175	-2.0653749	2.3543476
21 H	H7	2.1095927	-3.9976793	0.1429447
22 H	H8	2.2725783	-5.7381236	1.8901922
23 H	H9	-0.8345080	-3.7860902	4.1299055
24 H	H10	0.8054311	-5.6361704	3.8917415
25 C	C13	-1.3750083	3.2903605	0.2075104
26 C	C14	-2.9714977	5.5624858	0.5608659
27 C	C15	-1.4171872	4.2839924	-0.7694330
28 C	C16	-2.1280442	3.4581605	1.3746779
29 C	C17	-2.9196823	4.5840422	1.5506910
30 C	C18	-2.2162982	5.4124915	-0.5951734
31 H	H11	-0.8208120	4.1909762	-1.6755921
32 H	H12	-2.0875046	2.6996259	2.1556109
33 H	H13	-3.5025588	4.6961613	2.4634274
34 H	H14	-2.2411274	6.1781330	-1.3689752
35 H	H15	-3.5966322	6.4436315	0.6952106
36 C	C19	0.3093955	-2.3625184	-1.7547497
37 C	C20	-0.0912534	-3.3543209	-4.3401231
38 C	C21	-0.0343090	-3.6998540	-1.9510869
39 C	C22	0.4315207	-1.5226782	-2.8671081
40 C	C23	0.2385005	-2.0150756	-4.1517157
41 C	C24	-0.2283105	-4.1923834	-3.2382123
42 H	H16	-0.1801437	-4.3563152	-1.0943887
43 H	H17	0.6652193	-0.4667534	-2.7294260
44 H	H18	0.3368203	-1.3430770	-5.0036115
45 H	H19	-0.5029581	-5.2366847	-3.3778691
46 H	H20	-0.2505127	-3.7434574	-5.3443139
47 C	C25	2.2037840	-1.0561819	-0.0322183
48 C	C26	4.8398877	-0.1467124	0.2959495
49 C	C27	3.1019531	-1.0752640	-1.1008785
50 C	C28	2.6607231	-0.6041884	1.2108398
51 C	C29	3.9595015	-0.1434535	1.3759256
52 C	C30	4.4101610	-0.6253487	-0.9360627
53 H	H21	2.7870258	-1.4445361	-2.0751182
54 H	H22	1.9885174	-0.6163461	2.0684050
55 H	H23	4.2877890	0.2105737	2.3517687
56 H	H29	5.0954406	-0.6518652	-1.7824718
57 H	H30	5.8606631	0.2109369	0.4199248
58 C	C31	0.8581226	2.2401004	1.4549467
59 C	C32	2.4867689	2.9722155	3.6076359
60 C	C33	1.7419847	3.3175634	1.3410746
61 C	C34	0.7782282	1.5572999	2.6702650
62 C	C35	1.5854226	1.9217570	3.7439218
63 C	C36	2.5621159	3.6713663	2.4050328
64 H	H31	1.7796307	3.8926768	0.4153647
65 H	H32	0.0785737	0.7243919	2.7718073
66 H	H34	1.5101728	1.3824088	4.6865585
67 H	H35	3.2539301	4.5054985	2.3011726
68 H	H36	3.1225296	3.2568432	4.4443528
69 C	C37	0.5448527	1.9591246	-1.4678986
70 C	C38	1.7126695	1.9701881	-4.0147177
71 C	C39	1.9329834	1.9560158	-1.6093465
72 C	C40	-0.2538845	1.9393876	-2.6219998
73 C	C41	0.3246039	1.9591404	-3.8847274
74 C	C42	2.5113060	1.9621411	-2.8770888
75 H	H33	2.5718828	1.9419231	-0.7270615
76 H	H37	-1.3404477	1.8961243	-2.5257703
77 H	H38	-0.3095838	1.9503417	-4.7696821
78 H	H39	3.5965836	1.9510560	-2.9680625
79 H	H40	2.1686803	1.9780664	-5.0031796
80 Ni	Ni1	-1.2881052	-0.1951646	0.1478866
81 C	C43	-3.6440352	0.3531311	2.6242186
82 H	H26	-2.6450086	-0.0884689	2.7577006
83 H	H41	-4.3809839	-0.3829067	2.9818466
84 H	H42	-3.7185466	1.2177751	3.3053505
85 H	H43	-4.8371240	1.2220295	0.9956580

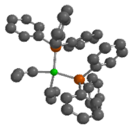

$C_{42}H_{40}NiP_2$, *ZZ cis anti* –3814.62096 au
M06/6-311+G(d,p)//M06/6-31G(d)

1 C	C2	-3.0389505	-2.2729112	-1.1012828
2 C	C3	-2.4585970	-1.8024341	0.0100757
3 H	H6	-2.6761599	-2.3571176	0.9325480
4 P	P1	0.3759941	-1.6566620	-0.0947719
5 P	P2	-0.3870007	1.6956464	0.1950004
6 C	C10	-3.0658369	0.4255045	0.4187211
7 H	H25	-3.4991092	0.7889235	-0.5257069
8 C	C11	-3.8191969	0.5745617	1.5137089
9 C	C4	0.4383053	-3.0164164	1.1355879
10 C	C5	0.6471052	-4.9610030	3.1350689
11 C	C6	-0.3375211	-2.9308951	2.2924845
12 C	C7	1.3376000	-4.0799330	1.0007602
13 C	C8	1.4342333	-5.0509682	1.9891159
14 C	C9	-0.2333018	-3.8967464	3.2891247
15 H	H3	-1.0316301	-2.0983599	2.4084678
16 H	H7	1.9764148	-4.1403125	0.1186198
17 H	H8	2.1342673	-5.8761917	1.8702594
18 H	H9	-0.8460494	-3.8173868	4.1852930
19 H	H10	0.7283405	-5.7197739	3.9115941
20 C	C13	-1.4477004	3.1995430	0.2202119
21 C	C14	-3.0588681	5.4742497	0.4920427
22 C	C15	-1.4973260	4.1579965	-0.7917853

23 C	C16	-2.2016205	3.4055823	1.3813519
24 C	C17	-2.9997422	4.5327605	1.5162803
25 C	C18	-2.3033602	5.2866113	-0.6584316
26 H	H11	-0.9002708	4.0385040	-1.6941554
27 H	H12	-2.1554495	2.6784108	2.1907649
28 H	H13	-3.5810990	4.6759667	2.4254651
29 H	H14	-2.3325399	6.0233663	-1.4596219
30 H	H15	-3.6890235	6.3560971	0.5946566
31 C	C19	0.2892058	-2.4535303	-1.7354136
32 C	C20	0.0074403	-3.4866930	-4.3206063
33 C	C21	-0.1529495	-3.7634410	-1.9265087
34 C	C22	0.5543653	-1.6578078	-2.8548023
35 C	C23	0.4261555	-2.1717655	-4.1388683
36 C	C24	-0.2883991	-4.2757483	-3.2131344
37 H	H16	-0.4090510	-4.3807435	-1.0669391
38 H	H17	0.8532493	-0.6181840	-2.7250669
39 H	H18	0.6429987	-1.5341408	-4.9953174
40 H	H19	-0.6360519	-5.2983377	-3.3498420
41 H	H20	-0.1005772	-3.8944705	-5.3243008
42 C	C25	2.1496545	-1.1387708	0.0361424
43 C	C26	4.7742618	-0.2327738	0.4626202
44 C	C27	3.0935839	-1.1788571	-0.9915503
45 C	C28	2.5559057	-0.6692918	1.2903540
46 C	C29	3.8491304	-0.2122356	1.5039562
47 C	C30	4.3947581	-0.7287413	-0.7790154
48 H	H21	2.8224923	-1.5655457	-1.9720046
49 H	H22	1.8476332	-0.6662068	2.1183069
50 H	H23	4.1389799	0.1498612	2.4888273
51 H	H29	5.1153624	-0.7714040	-1.5948533
52 H	H30	5.7902083	0.1234884	0.6243575
53 C	C31	0.7717567	2.1845524	1.5282659
54 C	C32	2.3612762	2.9620092	3.6944248
55 C	C33	1.6501754	3.2660804	1.4110282
56 C	C34	0.6764974	1.5211990	2.7534439
57 C	C35	1.4639508	1.9083982	3.8337262
58 C	C36	2.4514279	3.6419296	2.4819896
59 H	H31	1.6987503	3.8267050	0.4769306
60 H	H32	-0.0228643	0.6886052	2.8587205
61 H	H34	1.3756263	1.3852987	4.7843422
62 H	H35	3.1398717	4.4785317	2.3755511
63 H	H36	2.9825110	3.2635633	4.5361183
64 C	C37	0.5059432	1.8429978	-1.3959617
65 C	C38	1.7123076	1.8083775	-3.9256374
66 C	C39	1.8961838	1.8536523	-1.5173704
67 C	C40	-0.2742739	1.7818880	-2.5613245
68 C	C41	0.3228319	1.7775036	-3.8157702
69 C	C42	2.4934539	1.8388574	-2.7761588
70 H	H33	2.5241771	1.8616158	-0.6274578
71 H	H37	-1.3621310	1.7305142	-2.4805412
72 H	H38	-0.2982436	1.7364429	-4.7090138
73 H	H39	3.5800420	1.8393438	-2.8504684
74 H	H40	2.1830064	1.7986650	-4.9072376
75 Ni	Ni1	-1.3438780	-0.2873738	0.2112540
76 C	C43	-3.4284538	0.1468333	2.8936484
77 H	H26	-2.4351808	-0.3274323	2.8879074
78 H	H41	-4.1389474	-0.5744818	3.3258648
79 H	H42	-3.3830165	0.9953780	3.5979346
80 H	H43	-4.8009693	1.0563251	1.4339591
81 C	C12	-2.9785131	-1.6390003	-2.4540182
82 H	H5	-2.3206386	-0.7575753	-2.4511410
83 H	H24	-3.9756428	-1.3083976	-2.7856953
84 H	H27	-2.6033516	-2.3328615	-3.2225184
85 H	H28	-3.6539483	-3.1794178	-1.0425838

M06/6-31G(d)·LANL2DZ geometries _____

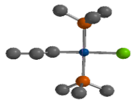

$C_9H_{23}ClP_2Pd$, *E trans* –1627.53673 au
M06/6-311+G(d,p)·LANL2DZ//...

1 H	H1	1.6190627	-2.4397901	2.7297795
2 C	C1	1.6149985	-1.3793366	3.0148430
3 H	H2	1.0776524	-1.2914876	3.9709153
4 H	H4	2.6549806	-1.0812097	3.2149626
5 C	C2	0.9876631	-0.5381172	1.9426870
6 H	H5	0.9437479	0.5392500	2.1461016
7 C	C3	0.4886852	-1.0138718	0.7982972
8 H	H6	0.5440000	-2.0969089	0.6111816
9 P	P1	1.7964289	0.3320505	-1.5883487
10 P	P2	-2.4306199	0.0296305	0.3618371
11 C	C4	1.8887671	0.5757271	-3.3959084
12 H	H10	2.9055544	0.8538407	-3.7040045
13 H	H13	1.1747216	1.3596591	-3.6730827
14 H	H14	1.5916252	-0.3461166	-3.9088600
15 C	C5	3.0778895	-0.9356188	-1.2674919
16 H	H3	4.0422560	-0.6354575	-1.6986702
17 H	H15	2.7710152	-1.8928256	-1.7054344
18 H	H16	3.1854585	-1.0728763	-0.1854333
19 C	C6	2.5498463	1.8540842	-0.9117395
20 H	H9	3.5541650	2.0274989	-1.3217102
21 H	H17	2.6120469	1.7649791	0.1798126
22 H	H18	1.9019149	2.7045382	-1.1555870
23 C	C7	-3.7921400	-0.4848179	-0.7440362
24 H	H11	-3.6600365	-1.5344526	-1.0305186
25 H	H19	-3.7486728	0.1301101	-1.6503455
26 H	H20	-4.7672773	-0.3630167	-0.2532254
27 C	C8	-2.7001799	-0.9879734	1.8586903
28 H	H8	-2.4918457	-2.0407776	1.6344426
29 H	H21	-3.7336066	-0.8908344	2.2174886
30 H	H22	-2.0083027	-0.6713046	2.6473657
31 C	C9	-2.9368940	1.7013400	0.9007396
32 H	H12	-2.2261069	2.0751925	1.6475166
33 H	H23	-3.9478512	1.6954619	1.3303897
34 H	H24	-2.9024683	2.3635342	0.0275234
35 Cl	Cl1	-1.2969014	1.5906811	-2.3669486
36 Pd	Pd1	-0.3395764	0.0992155	-0.6732287

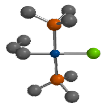

$C_9H_{23}ClP_2Pd$, *Z trans* –1627.54074 au
M06/6-311+G(d,p)·LANL2DZ//...

1 C	C2	2.4425571	-0.0003952	0.4997063
2 C	C3	1.6119632	-0.0002079	-0.5438824
3 H	H6	2.0288097	-0.0008994	-1.5582538
4 P	P1	-0.4528321	2.3316521	-0.3504022
5 P	P2	-0.4524833	-2.3316415	-0.3509431
6 C	C4	-1.5241542	3.1271251	-1.5981420
7 H	H10	-1.6235749	4.2038130	-1.4038669
8 H	H13	-2.5083215	2.6459429	-1.5576617
9 H	H14	-1.1060803	2.9774394	-2.5996942
10 C	C5	1.0942356	3.3042841	-0.4392968
11 H	H3	0.8859230	4.3786060	-0.3460468
12 H	H15	1.6005296	3.1147569	-1.3925593
13 H	H16	1.7689035	2.9911836	0.3654807
14 C	C6	-1.1861882	2.8756838	1.2325371
15 H	H9	-1.2949774	3.9681997	1.2673216
16 H	H17	-0.5486631	2.5493434	2.0631609
17 H	H18	-2.1676442	2.3974880	1.3389207
18 C	C7	1.0946413	-3.3044203	-0.4413327
19 H	H11	1.7693878	-2.9932177	0.3639557
20 H	H19	1.6008681	-3.1131418	-1.3936663
21 H	H20	0.8866285	-4.3785011	-0.3503800
22 C	C8	-1.1845082	-2.8751308	1.2332003
23 H	H8	-0.5495572	-2.5440178	2.0632609
24 H	H21	-1.2889725	-3.9679669	1.2713301
25 H	H22	-2.1672259	-2.4008982	1.3365157
26 C	C9	-1.5262633	-3.1275781	-1.5976374
27 H	H12	-2.5110303	-2.6476617	-1.5554830
28 H	H23	-1.6245166	-4.2037217	-1.4030371
29 H	H24	-1.1103073	-2.9777037	-2.6006066
30 Cl	Cl1	-2.8673529	-0.0004842	-0.3267863
31 Pd	Pd1	-0.4120378	-0.0000031	-0.4623336
32 C	C10	2.0316172	0.0005865	1.9390761
33 H	H5	2.4195494	0.8793715	2.4772331
34 H	H7	2.4119911	-0.8823023	2.4773650
35 H	H25	0.9350149	0.0056074	2.0275986
36 H	H26	3.5240710	-0.0011897	0.3153562

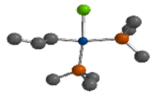

$C_9H_{23}ClP_2Pd$, *E cis* –1627.51893 au
M06/6-311+G(d,p)·LANL2DZ//...

1 H	H1	0.6406051	-1.7024743	4.8437742
2 C	C1	0.8565652	-0.6390932	4.6807297
3 H	H2	0.2564333	-0.0622961	5.3980486
4 H	H4	1.9120548	-0.4612596	4.9380277
5 C	C2	0.5491402	-0.2387493	3.2686539
6 H	H5	0.7434667	0.8137571	3.0214770
7 C	C3	0.0453608	-1.0543484	2.3440896
8 H	H6	-0.1842053	-2.0997842	2.5771651
9 P	P1	1.5046340	-0.0750460	-0.1136923
10 C	C4	2.9112954	-0.6461656	0.9073396
11 H	H10	3.8587836	-0.4525625	0.3863230
12 H	H13	2.8089661	-1.7197096	1.1005945
13 H	H14	2.9088354	-0.1358248	1.8751643
14 C	C5	1.9523542	1.6764391	-0.4183520
15 H	H3	2.9833425	1.7659785	-0.7888416
16 H	H15	1.8587993	2.2397733	0.5187422
17 H	H16	1.2655456	2.1166993	-1.1512892
18 C	C6	1.8879348	-0.8859434	-1.7140899
19 H	H9	2.9166143	-0.6712955	-2.0347758
20 H	H17	1.1917438	-0.5446863	-2.4887640
21 H	H18	1.7653517	-1.9696925	-1.6042923
22 Pd	Pd1	-0.6534067	-0.4524646	0.5356398
23 Cl	Cl2	-2.8082717	-1.2317800	1.2779261
24 P	P2	-1.8659012	0.2857714	-1.4544129
25 C	C7	-1.1838776	1.2924180	-2.8434935
26 H	H7	-0.3406976	0.7850586	-3.3286614
27 H	H12	-0.8353955	2.2642884	-2.4692881
28 H	H19	-1.9571340	1.4747005	-3.6038655
29 C	C8	-2.5914603	-1.1354832	-2.3525937
30 H	H11	-1.7901116	-1.7443894	-2.7897131
31 H	H20	-3.2719596	-0.8050859	-3.1490309
32 H	H21	-3.1363042	-1.7505932	-1.6270640
33 C	C9	-3.3321574	1.2897304	-1.0127091
34 H	H8	-3.0073108	2.2551055	-0.6061863
35 H	H22	-3.8882136	0.7567395	-0.2333486
36 H	H23	-3.9714194	1.4622678	-1.8892310

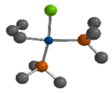

$C_9H_{23}ClP_2Pd$, *E cis* –1627.52402 au
M06/6-311+G(d,p)·LANL2DZ//...

1 C	C2	1.8187020	0.9319356	2.8082919
2 C	C3	1.8301771	-0.0386495	1.8977267
3 H	H6	2.4250745	-0.9454477	2.0513150
4 P	P2	-0.7128213	-1.0620978	1.1112050
5 C	C7	-2.0723926	-1.7603392	0.0942885
6 H	H11	-1.6626190	-2.4650000	-0.6391562
7 H	H19	-2.5886541	-0.9586913	-0.4462426
8 H	H20	-2.7999356	-2.2878528	0.7264143
9 C	C8	-0.1918295	-2.5358373	2.0646738
10 H	H8	0.3279511	-3.2413114	1.4060389
11 H	H21	-1.0611380	-3.0332524	2.5154017
12 H	H22	0.5001135	-2.2283983	2.8560661
13 C	C9	-1.6360811	-0.0932675	2.3584252
14 H	H12	-0.9271787	0.2841895	3.1038161
15 H	H23	-2.3949274	-0.7164365	2.8503437
16 H	H24	-2.1247496	0.7630433	1.8792816
17 Pd	Pd1	0.9620910	0.0771692	0.0497420
18 C	C10	1.1326124	2.2536285	2.6719077
19 H	H5	1.8727905	3.0600397	2.5682587
20 H	H7	0.5111352	2.4981490	3.5467581
21 H	H25	0.5050509	2.2781950	1.7703085
22 H	H26	2.4145751	0.7989569	3.7201572
23 Cl	Cl1	2.9023009	1.2913684	-0.6961761
24 P	P1	0.2182234	0.3702664	-2.2561295
25 C	C1	0.1914416	2.1429402	-2.7181972
26 H	H1	1.1470872	2.5866046	-2.4162095
27 H	H3	-0.6154808	2.6555103	-2.1807754
28 H	H9	0.0418948	2.2687631	-3.7994042
29 C	C4	-1.3728245	-0.1960445	-2.9989457
30 H	H4	-1.4711940	-1.2857032	-2.9285662
31 H	H10	-1.4203037	0.0892325	-4.0589405
32 H	H13	-2.2219418	0.2657286	-2.4796601
33 C	C5	1.4284838	-0.3335436	-3.4382371
34 H	H2	1.4254774	-1.4277434	-3.3674366
35 H	H14	2.4231033	0.0306970	-3.1562564
36 H	H15	1.1957862	-0.0368015	-4.4700873

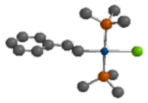

$C_{14}H_{25}ClP_2Pd$, *E trans* –1819.17397 au
M06/6-311+G(d,p)·LANL2DZ//...

1 C	C2	0.4528797	0.0449809	1.1843650
2 H	H5	0.2275117	1.1167803	1.2575454

3 C	C3	0.0867110	-0.6259681	0.0821808
4 H	H6	0.2858510	-1.7043561	0.0193139
5 P	P1	1.3905979	0.5071481	-2.4199783
6 P	P2	-2.8644317	0.1240555	-0.5475904
7 C	C4	2.7942981	-0.4429365	-1.7323598
8 H	H10	3.7426949	-0.1147952	-2.1776320
9 H	H13	2.6522617	-1.5105542	-1.9358659
10 H	H14	2.8311017	-0.3063924	-0.6438703
11 C	C5	1.8873186	2.2453258	-2.1476201
12 H	H3	2.8778907	2.4536984	-2.5747028
13 H	H15	1.9062266	2.4521483	-1.0710949
14 H	H16	1.1380566	2.8963029	-2.6149923
15 C	C6	1.5646635	0.2891508	-4.2248065
16 H	H9	2.5438974	0.6452669	-4.5720563
17 H	H17	0.7590624	0.8501356	-4.7131998
18 H	H18	1.4497536	-0.7686923	-4.4843994
19 C	C7	-4.2364171	-0.3520247	-1.6544383
20 H	H11	-4.1347374	-1.4053359	-1.9392187
21 H	H19	-4.1690732	0.2571344	-2.5636830
22 H	H20	-5.2078308	-0.2003327	-1.1656650
23 C	C8	-3.1436475	-0.8946429	0.9456658
24 H	H8	-2.9909416	-1.9536881	0.7046138
25 H	H21	-4.1624652	-0.7557871	1.3306231
26 H	H22	-2.4167251	-0.6194425	1.7176414
27 C	C9	-3.3306410	1.8050248	-0.0009673
28 H	H12	-2.5973549	2.1719668	0.7277628
29 H	H23	-4.3311965	1.8165773	0.4526066
30 H	H24	-3.3133108	2.4660199	-0.8753381
31 Cl	Cl1	-1.7172261	1.3774835	-3.4633432
32 Pd	Pd1	-0.7572564	0.2039798	-1.5476510
33 C	C1	1.1377746	-0.5133374	2.3588737
34 C	C10	2.4125726	-1.5200504	4.6574697
35 C	C11	1.7725492	-1.7632254	2.3430017
36 C	C12	1.1774061	0.2255442	3.5473182
37 C	C13	1.8032495	-0.2703273	4.6845977
38 C	C14	2.3975937	-2.2620201	3.4776409
39 H	H2	1.7827814	-2.3450353	1.4220779
40 H	H1	0.6978138	1.2055361	3.5728874
41 H	H4	1.8124160	0.3230650	5.5980275
42 H	H7	2.8836073	-3.2366570	3.4404794
43 H	H25	2.9047144	-1.9117238	5.5457806

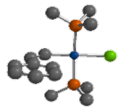

$C_{14}H_{25}ClP_2Pd$, *Z trans* −1819.18127 au
M06/6-311+G(d,p)·LANL2DZ//...

1 C	C2	1.8343535	-0.0125547	-1.1300705
2 C	C3	0.5934620	0.0097064	-1.6390193
3 H	H6	0.5028067	0.0250044	-2.7320421
4 P	P1	-1.2010548	2.3657609	-0.6689520
5 P	P2	-1.1691160	-2.3046494	-0.5019011
6 C	C4	0.2068315	3.3026788	-1.3634912
7 H	H10	0.0623428	4.3833108	-1.2309302
8 H	H13	0.3170460	3.0804606	-2.4315172
9 H	H14	1.1293514	2.9917711	-0.8588194
10 C	C5	-1.2852611	2.9709620	1.0542756
11 H	H3	-1.3886020	4.0640136	1.0848722
12 H	H15	-0.3732133	2.6792471	1.5889972
13 H	H16	-2.1449125	2.4997454	1.5448737
14 C	C6	-2.6653948	3.1251513	-1.4548045
15 H	H9	-2.6920452	4.2079874	-1.2741565
16 H	H17	-3.5608539	2.6531231	-1.0343026
17 H	H18	-2.6489489	2.9379590	-2.5342366
18 C	C7	-2.5998549	-3.1216248	-1.2931536
19 H	H11	-2.5510633	-2.9898479	-2.3801173
20 H	H19	-3.5128588	-2.6389591	-0.9251030
21 H	H20	-2.6213662	-4.1941557	-1.0578863
22 C	C8	0.2677447	-3.2726953	-1.0863563
23 H	H8	0.4117906	-3.1208056	-2.1618347
24 H	H21	0.1226561	-4.3426211	-0.8852741
25 H	H22	1.1711672	-2.9234328	-0.5722914
26 C	C9	-1.3146044	-2.8054967	1.2493282
27 H	H12	-0.4254914	-2.4736652	1.7995262
28 H	H23	-1.4106245	-3.8960744	1.3394859
29 H	H24	-2.1961578	-2.3153201	1.6781884
30 Cl	Cl1	-3.3904435	0.0528737	0.3837088
31 Pd	Pd1	-1.1936304	0.0267816	-0.7085392
32 H	H26	2.6733546	-0.0236666	-1.8381030
33 C	C1	2.2625572	-0.0394017	0.2729477
34 C	C10	3.2251329	-0.1229709	2.9264852
35 C	C1	3.6332505	-0.1511517	0.5517215
36 C	C12	1.3859089	0.0360637	1.3675627
37 C	C13	1.8608350	-0.0067652	2.6726055
38 C	C14	4.1105975	-0.1937596	1.8552503
39 H	H2	4.3341242	-0.2075280	-0.2827997
40 H	H1	0.3110069	0.1302783	1.1899523
41 H	H5	1.1546500	0.0531364	3.5005921
42 H	H4	5.1809072	-0.2830746	2.0351220
43 H	H7	3.5936203	-0.1557943	3.9502062

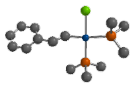

$C_{14}H_{25}ClP_2Pd$, *E cis* −1819.15565 au
M06/6-311+G(d,p)·LANL2DZ//...

1 C	C2	0.2506237	-0.1224470	2.1466977
2 H	H5	0.4468822	0.9324313	1.9127350
3 C	C3	-0.2323636	-0.9253370	1.1930076
4 H	H6	-0.4371263	-1.9840640	1.3786345
5 P	P1	1.2584055	0.1629003	-1.2064786
6 C	C4	2.6555372	-0.3474991	-0.1416182
7 H	H10	3.6075276	-0.1365672	-0.6477441
8 H	H13	2.5810084	-1.4191741	0.0729166
9 H	H14	2.6234678	0.1816529	0.8165931
10 C	C5	1.6685137	1.9183103	-1.5400968
11 H	H3	2.7031922	2.0226574	-1.8940989
12 H	H15	1.5467681	2.4985633	-0.6173699
13 H	H16	0.9861514	2.3282352	-2.2933039
14 C	C6	1.6916532	-0.6741550	-2.7803492
15 H	H9	2.7148231	-0.4265859	-3.0945279
16 H	H17	0.9918775	-0.3851555	-3.5728471
17 H	H18	1.6120031	-1.7587296	-2.6411432
18 Pd	Pd1	-0.9061747	-0.2747147	-0.6033938
19 Cl	Cl2	-3.0488999	-1.1474050	0.0569256
20 P	P2	-2.0867320	0.4929846	-2.5916795
21 C	C7	-1.3984950	1.5927408	-3.9036149
22 H	H7	-0.4914596	1.1695016	-4.3520715
23 H	H12	-1.1475885	2.5706779	-3.4733485
24 H	H19	-2.1369818	1.7493451	-4.7018594
25 C	C8	-2.7070397	-0.9220165	3.5731641
26 H	H11	-1.8630440	-1.4703602	-4.0103461
27 H	H20	-3.3798579	-0.5927302	-4.3765481
28 H	H21	-3.2410917	-1.5953204	-2.8923721
29 C	C9	-3.6166218	1.4050912	-2.1692469
30 H	H8	-3.3590337	2.3438639	-1.6648820
31 H	H22	-4.2000191	0.7917844	-1.4736436
32 H	H23	-4.2053072	1.6261248	-3.0699019
33 C	C1	0.5909077	-0.4973337	3.5253911
34 C	C10	1.3264750	-1.1486218	6.1610345
35 C	C11	0.1598409	-1.6920013	4.1179578
36 C	C12	1.3844956	0.3666193	4.2895904
37 C	C13	1.7511747	0.0471578	5.5914874
38 C	C14	0.5272310	-2.0141837	5.4164991
39 H	H2	-0.4891887	-2.3625053	3.5558523
40 H	H1	1.7173680	1.3066307	3.8453456
41 H	H4	2.3696117	0.7365411	6.1648512
42 H	H24	0.1755254	-2.9458880	5.8576153
43 H	H25	1.6059604	-1.4010190	7.1825155

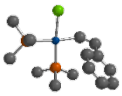

$C_{14}H_{25}ClP_2Pd$, *Z cis* −1819.16583 au
M06/6-311+G(d,p)·LANL2DZ//...

1 C	C2	2.3925349	1.4688099	1.4277245
2 C	C3	2.6041716	0.3905617	0.6670604
3 H	H6	3.5822990	-0.0986422	0.6674287
4 P	P2	0.1156466	-1.2897750	1.0871317
5 C	C7	-0.2380427	-3.0854357	0.9493815
6 H	H11	0.7043095	-3.6445041	0.9228673
7 H	H19	-0.7851609	-3.2935010	0.0231089
8 H	H20	-0.8366136	-3.4313786	1.8031444
9 C	C8	0.7437339	-1.1775524	2.8007777
10 H	H8	1.7340256	-1.6405117	2.8714844
11 H	H21	0.0510779	-1.6859625	3.4852605
12 H	H22	0.8361887	-0.1249227	3.0895757
13 C	C9	-1.5578408	-0.5581907	1.2564845
14 H	H12	-1.4638288	0.4980236	1.5399735
15 H	H23	-2.1423988	-1.0897267	2.0200668
16 H	H24	-2.0896149	-0.6033945	0.2974101
17 Pd	Pd1	1.3069221	-0.4243057	-0.6704300
18 H	H26	3.1943446	1.8164933	2.0914403
19 Cl	Cl1	2.6542930	0.6104586	-2.3805980
20 P	P1	-0.1190093	-1.1229938	-2.5105045
21 C	C1	-0.8818220	0.3871353	-3.2156064
22 H	H1	-1.5719155	0.8238973	-2.4812446
23 H	H3	-1.4329731	0.1666802	-4.1398755
24 H	H9	-0.0858130	1.1124129	-3.4213594
25 C	C4	-1.5849233	-2.2383162	-2.3994526
26 H	H4	-1.2663733	-3.2619243	-2.1648753
27 H	H10	-2.1277877	-2.2582608	-3.3544871
28 H	H13	-2.2773587	-1.9030121	-1.6164482
29 C	C5	0.7577801	-1.8529859	-3.9406920
30 H	H2	1.1286039	-2.8501839	-3.6760407
31 H	H14	1.6172656	-1.2154131	-4.1754099
32 H	H15	0.0930914	-1.9314975	-4.8114625
33 C	C6	1.1398997	2.2328161	1.5059562
34 C	C10	-1.3124522	3.5953151	1.7300310
35 C	C11	0.7043821	2.7401604	2.7379410
36 C	C12	0.3387906	2.4630278	0.3770327
37 C	C13	-0.8742048	3.1337557	0.4908326
38 C	C14	-0.5124387	3.4025059	2.8540581
39 H	H5	1.3314668	2.5928221	3.6192822
40 H	H7	0.7026481	2.1359481	-0.5979843
41 H	H16	-1.4738197	3.3161799	-0.4003483
42 H	H17	-0.8371234	3.7742922	3.8249210
43 H	H18	-2.2619605	4.1210949	1.8164438

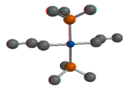

$C_{12}H_{28}P_2Pd$, *EE trans* −1284.50972 au
M06/6-311+G(d,p)·LANL2DZ//...

1 H	H1	4.7687842	0.0092422	-0.2698182
2 C	C1	4.3306305	0.0058094	0.7376674
3 H	H2	4.7220641	-0.8748718	1.2697641
4 H	H4	4.7179712	0.8857861	1.2739207
5 C	C2	2.8303495	0.0026068	0.6668417
6 H	H5	2.3217632	-0.0005405	1.6427085
7 C	C3	2.0965420	0.0033839	-0.4551034
8 H	H6	2.6703119	0.0064880	-1.3985012
9 P	P1	0.0028836	-2.3071175	-0.3943598
10 C	C4	0.0015550	-2.7915030	1.3704198
11 H	H10	0.0036348	-3.8832784	1.4931201
12 H	H13	-0.8902291	-2.3715100	1.8512082
13 H	H14	0.8901940	-2.3677583	1.8537624
14 C	C5	1.4311142	-3.2363092	-1.0636888
15 H	H3	1.3570264	-4.3067659	-0.8286247
16 H	H15	2.3510521	-2.8232111	-0.6346373
17 H	H16	1.4772312	-3.1093465	-2.1518596
18 C	C6	-1.4218198	-3.2396668	-1.0666440
19 H	H9	-1.3457859	-4.3098788	-0.8311118
20 H	H17	-1.4657615	-3.1131416	-2.1549586
21 H	H18	-2.3436831	-2.8285774	-0.6397716
22 Pd	Pd1	0.0000000	0.0000000	-0.5406688
23 P	P2	-0.0028836	2.3071175	-0.3943597
24 C	C7	-1.4311142	3.2363091	-1.0636888
25 H	H7	-1.4772310	3.1093465	-2.1518596
26 H	H12	-1.3570265	4.3067658	-0.8286247
27 H	H19	-2.3510521	2.8232109	-0.6346375
28 C	C8	-0.0015551	2.7915030	1.3704199
29 H	H11	-0.0036346	3.8832784	1.4931201
30 H	H20	0.8902289	2.3715099	1.8512084
31 H	H21	-0.8901941	2.3677585	1.8537624
32 C	C9	1.4218198	3.2396667	-1.0666438
33 H	H8	1.4657616	3.1131414	-2.1549585
34 H	H22	2.3436831	2.8285775	-0.6397714
35 H	H23	1.3457857	4.3098788	-0.8311119
36 C	C10	-2.0965420	-0.0033839	-0.4551034
37 H	H25	-2.6703120	-0.0064880	-1.3985012
38 C	C11	-2.8303494	-0.0026067	0.6668418
39 H	H26	-2.3217632	0.0005406	1.6427085
40 C	C12	-4.3306304	-0.0058094	0.7376675
41 H	H24	-4.7220641	0.8748720	1.2697640
42 H	H27	-4.7179712	-0.8857860	1.2739210
43 H	H28	-4.7687842	-0.0092425	-0.2698182

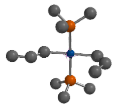

$C_{12}H_{28}P_2Pd$, *ZE trans* −1284.51223 au
M06/6-311+G(d,p)·LANL2DZ//...

1 H H1	-0.2496353	-1.4768810	4.3120362
2 C C1	0.7432664	-1.2086203	3.9256555
3 H H2	1.1668836	-0.4433880	4.5939987
4 H H4	1.3903821	-2.0938745	4.0229653
5 C C2	0.6508166	-0.7319228	2.5044255
6 H H5	1.6110157	-0.4441671	2.0493806
7 C C3	-0.4717765	-0.6386780	1.7779649
8 H H6	-1.3990044	-0.9448603	2.2938475
9 P P1	-0.2194928	-2.1713050	-0.9053426
10 C C4	-0.8940299	-3.5160318	0.1396774
11 H H10	-0.6005916	-4.5036970	-0.2409929
12 H H13	-1.9881611	-3.4494986	0.1661753
13 H H14	-0.5196360	-3.3843392	1.1614056
14 C C5	1.5698441	-2.5607147	-0.9410316
15 H H3	1.7478530	-3.6070257	-1.2253098
16 H H15	1.9950810	-2.3755478	0.0529092
17 H H16	2.0661874	-1.8988381	-1.6608910
18 C C6	-0.7613014	-2.6606976	-2.5850112
19 H H9	-0.4519108	-3.6874263	-2.8228037
20 H H17	-0.3290787	-1.9593908	-3.3083102
21 H H18	-1.8530347	-2.5864868	-2.6560394
22 Pd Pd1	-0.5842832	0.0091937	-0.2164745
23 P P2	-0.7822090	2.1906406	0.5398413
24 C C7	0.8567028	2.9082078	0.9269116
25 H H7	1.4639302	2.9366035	0.0145702
26 H H12	1.3596752	2.2671995	1.6607268
27 H H19	0.7624074	3.9245242	1.3331543
28 C C8	-1.5022476	3.4456021	-0.5827911
29 H H11	-0.9435878	3.4238480	-1.5257169
30 H H20	-1.4590731	4.4508987	-0.1423831
31 H H21	-2.5457007	3.1911083	-0.8035677
32 C C9	-1.7038407	2.4640749	2.0995290
33 H H8	-1.2540124	1.8398427	2.8805370
34 H H22	-2.7473011	2.1522872	1.9729473
35 H H23	-1.6758517	3.5195174	2.4027709
36 C C10	-0.5785193	0.6671126	-2.2107553
37 H H25	-1.5499227	0.7561510	-2.7217104
38 C C11	0.4963504	1.0082839	-2.9345100
39 C C13	1.9131246	0.9670397	-2.4430305
40 H H26	1.9492244	0.5907568	-1.4082441
41 H H29	2.3885439	1.9610654	-2.4581604
42 H H30	2.5510948	0.3190081	-3.0653831
43 H H31	0.3818191	1.3504258	-3.9729705

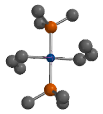

$C_{12}H_{28}P_2Pd$, *ZZ trans* –1284.51490 au

M06/6-311+G(d,p)·LANL2DZ//...

1 C C2	0.8709782	1.1126675	2.7693093
2 C C3	0.7965510	0.0566493	1.9494207
3 H H6	1.1928542	-0.8900420	2.3498703
4 P P1	2.1738483	0.2696614	-0.7545136
5 C C4	3.2731711	-1.0902821	-0.2082869
6 H H10	4.3039070	-0.9410503	-0.5574294
7 H H13	2.8929420	-2.0413566	-0.6010910
8 H H14	3.2609135	-1.1394601	0.8866312
9 C C5	3.0429993	1.7559311	-0.1325965
10 H H3	4.0915811	1.7722113	-0.4595340
11 H H15	2.9910588	1.7491334	0.9624499
12 H H16	2.5370639	2.6585565	-0.4949976
13 C C6	2.5153372	0.3354209	-2.5532065
14 H H9	3.5914504	0.4456695	-2.7456029
15 H H17	1.9754371	1.1767074	-3.0023015
16 H H18	2.1485823	-0.5826861	-3.0259621
17 Pd Pd1	-0.0005034	0.0007358	-0.0001468
18 P P2	-2.1748389	-0.2685385	0.7541009
19 C C7	-3.2764288	1.0878667	0.2037600
20 H H7	-3.2630819	1.1346104	-0.8912444
21 H H12	-2.8985066	2.0405683	0.5948550
22 H H19	-4.3072022	0.9373027	0.5522022
23 C C8	-3.0409432	-1.7580501	0.1357628
24 H H11	-2.9889910	-1.7535575	-0.9593154
25 H H2	-4.0894877	-1.7758488	0.4627300
26 H H21	-2.5331050	-2.6588076	0.5001014
27 C C9	-2.5166541	-0.3301465	2.5528737
28 H H8	-2.1497354	0.5888878	3.0236053
29 H H22	-1.9769140	-1.1704485	3.0039943
30 H H23	-3.5927897	-0.4397697	2.7454329
31 C C10	-0.7977027	-0.0568459	-1.9497069
32 H H25	-1.1953732	0.8889043	-2.3509858
33 C C11	-0.8708279	-1.1137720	-2.7685337
34 C C13	-0.3719874	-2.4890954	-2.4388425
35 H H26	0.0622696	-2.5077887	-1.4272935
36 H H29	0.4003741	-2.8292947	-3.1475754
37 H H30	-1.1724741	-3.2453318	-2.4760757
38 H H31	-1.3085418	-1.0115155	-3.7718478
39 C C12	0.3750378	2.4892987	2.4405755
40 H H5	1.1775201	3.2434743	2.4767768
41 H H24	-0.3954813	2.8313836	3.1503787
42 H H27	-0.0603886	2.5093261	1.4295445

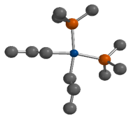

$C_{12}H_{28}P_2Pd$, *EE cis* –1284.50421 au

M06/6-311+G(d,p)·LANL2DZ//...

1 H H1.	3.0495283	-0.2962208	3.7363067
2 C C1	2.9897814	0.7496682	3.4062014
3 H H2	2.6726450	1.3531625	4.2698281
4 H H4	4.0082326	1.0812343	3.1519187
5 C C2	2.0422415	0.8898827	2.2508446
6 H H5	1.9230309	1.9088020	1.8604880
7 C C3	1.3403944	-0.1106668	1.7094239
8 H H6	1.4595072	-1.1121430	2.1496421
9 P P1	1.8971835	-0.1446849	-1.2918082
10 C C4	3.0672780	-1.4717374	-0.8045370
11 H H10	3.9728813	-1.4588028	-1.4263446
12 H H13	2.5800745	-2.4497862	-0.8965625
13 H H14	3.3408616	-1.3242221	0.2466072
14 C C5	2.9787788	1.3359777	-1.2360688
15 H H3	3.9141139	1.1705928	-1.7879591
16 H H15	3.2081545	1.5579284	-0.1870855
17 H H16	2.4544288	2.1977515	-1.6658488
18 C C6	1.7930420	-0.4343765	-3.1096126
19 H H9	2.7924959	-0.5186067	-3.5587195
20 H H17	1.2600703	0.3929156	-3.5942348
21 H H18	1.2400818	-1.3615118	-3.3053348
22 Pd Pd1	0.0013688	0.0028002	0.1534123
23 P P2	-1.8961171	0.1434673	-1.2901280
24 C C7	-1.7956983	0.4407093	-3.1069430
25 H H7	-1.2567151	-0.3806547	-3.5950001
26 H H12	-1.2501030	1.3728053	-3.2997465
27 H H19	-2.7961425	0.5188106	-3.5549474
28 C C8	-2.9668077	-1.3452328	-1.2388746
29 H H1	-2.4367671	-2.2013410	-1.6729692
30 H H20	-3.9041712	-1.1844795	-1.7886868
31 H H21	-3.1927765	-1.5731596	-0.1904121
32 C C9	-3.0753200	1.4601077	-0.7965872
33 H H8	-2.5954169	2.4420529	-0.8853705
34 H H22	-3.3468014	1.3066216	0.2542594
35 H H23	-3.9814505	1.4428860	-1.4175055
36 C C10	-1.3370971	0.1172955	1.7099028
37 H H25	-1.4510457	1.1176064	2.1541428
38 C C11	-2.0445762	-0.8816702	2.2468814
39 H H26	-1.9312475	-1.8994291	1.8517590
40 C C12	-2.9917679	-0.7412803	3.4025039
41 H H24	-2.6787522	-1.3510002	4.2632538
42 H H27	-4.0121005	-1.0653753	3.1461353
43 H H28	-3.0453008	0.3033032	3.7377755

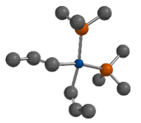

$C_{12}H_{28}P_2Pd$, *ZE cis* –1284.50757 au

M06/6-311+G(d,p)·LANL2DZ//...

1 H H1	1.4193321	-0.9125641	4.5441529
2 C C1	1.3657101	0.1779009	4.4245393
3 H H2	0.7032587	0.5668257	5.2122312
4 H H4	2.3664420	0.5888692	4.6277536
5 C C2	0.8723141	0.5412653	3.0545511
6 H H5	0.8011360	1.6173828	2.8511179
7 C C3	0.4964527	-0.3341734	2.1170758
8 H H6	0.5434864	-1.4037852	2.3737418
9 P P1	2.0538560	0.3506319	-0.4252851
10 C C4	3.1544442	-0.9890356	0.1748015
11 H H10	4.2054293	-0.7888852	-0.0746846
12 H H13	2.8526895	-1.9444953	-0.2706935
13 H H14	3.0421507	-1.0686057	1.2622398
14 C C5	2.8549617	1.8343092	0.2976867
15 H H3	3.9305682	1.8569364	0.0755616
16 H H15	2.7081475	1.8152319	1.3838824
17 H H16	2.3854231	2.7422142	-0.0990644
18 C C6	2.5982578	0.4802472	-2.1817370
19 H H9	3.6878775	0.6006890	-2.2569230
20 H H17	2.1136147	1.3385869	-2.6627799
21 H H18	2.3069574	-0.4260101	-2.7273048
22 Pd Pd1	-0.2231706	0.0726430	0.2342901
23 P P2	-1.5110190	0.4468310	-1.7375432
24 C C7	-0.8095955	1.0209651	-3.3440150
25 H H7	-0.0764499	0.2952558	-3.7167782
26 H H12	-0.3007105	1.9832941	-3.2058974
27 H H19	-1.5968346	1.1459024	-4.1003811
28 C C8	-2.4844572	-1.0101218	-2.2827214
29 H H11	-1.8127573	-1.8274270	-2.5712469
30 H H20	-3.1374898	-0.7606755	-3.1302919
31 H H21	-3.0945505	-1.3488323	-1.4370143
32 C C9	-2.8414822	1.6877998	-1.4905463
33 H H8	-2.4017776	2.6688573	-1.2748742
34 H H22	-3.4457389	1.3850460	-0.6276705
35 H H23	-3.4847466	1.7678227	-2.3775442
36 C C10	-2.0280783	-0.2115251	1.1910155
37 H H25	-2.4676512	0.6981755	1.6198772
38 C C11	-2.6819352	-1.3640661	1.3609193
39 C C13	-2.2047226	-2.7098933	0.9090920
40 H H26	-1.2537365	-2.6175046	0.3646921
41 H H29	-2.9306547	-3.2133745	0.2505964
42 H H30	-2.0383726	-3.3858139	1.7616149
43 H H31	-3.6365784	-1.3668947	1.9035636

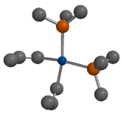

$C_{12}H_{28}P_2Pd$, *ZZ cis* –1284.50931 au

M06/6-311+G(d,p)·LANL2DZ//...

1 C C2	1.9934448	-0.2114979	2.6579410
2 C C3	1.0453107	-0.9761276	2.1098496
3 H H6	0.8376420	-1.9459231	2.5764393
4 P P1	1.6886102	-0.8412795	-1.0171056
5 C C4	3.2694910	-1.4422825	-0.3108926
6 H H10	4.0132691	-1.6217304	-1.0992829
7 H H1	3.0851297	-2.3689562	0.2440383
8 H H14	3.6552092	-0.7095327	0.4067428
9 C C5	2.2866460	0.5315953	-2.0897031
10 H H3	3.1074180	0.2055167	-2.7434293
11 H H15	2.6427706	1.3548410	-1.4579872
12 H H16	1.4663883	0.9112529	-2.7114661
13 C C6	1.3587333	-2.1581131	-2.2620149
14 H H9	2.2252679	-2.3255001	-2.9166935
15 H H17	0.4926208	-1.8920754	-2.8813251
16 H H18	1.1211305	-3.0919974	-1.7382368
17 Pd Pd1	-0.1275235	-0.4777971	0.4924954
18 P P2	-1.8451136	0.1308274	-1.0629335
19 C C7	-1.5447403	0.5806495	-2.8269851
20 H H7	-1.0142033	-0.2266796	-3.3481900
21 H H12	-0.9244761	1.4847927	-2.8759405
22 H H19	-2.4880920	0.7754811	-3.3558014
23 C C8	-3.0763516	-1.2195039	-1.2499293
24 H H11	-2.5907855	-2.1011532	-1.6870938
25 H H20	-3.9212010	-0.9181708	-1.8843626
26 H H21	-3.4485565	-1.4949603	-0.2558606
27 C C9	-2.9087934	1.5315412	-0.5449111
28 H H8	-2.3291179	2.4625144	-0.5574709
29 H H22	-3.2353812	1.3469781	0.4855405
30 H H23	-3.7809413	1.6387251	-1.2043017
31 C C10	-1.5764948	-0.4592430	1.9589975
32 H H25	-2.0421811	-1.4405855	2.1267025
33 C C11	-1.9726766	0.5547880	2.7323340
34 C C13	-1.4045373	1.9386787	2.7067058
35 H H26	-0.6811770	2.0403768	1.8850583
36 H H29	-0.8762354	2.1729761	3.6433929
37 H H30	-2.1824074	2.7091111	2.5831642
38 H H31	-2.7538611	0.3846414	3.4855078
39 C C12	2.3942325	1.1584107	2.2031184
40 H H5	3.4642191	1.2168901	1.9407869
41 H H24	2.2296482	1.9147431	2.9854641
42 H H27	1.8045174	1.4629454	1.3252790
43 H H28	2.5431489	-0.5851674	3.5323594

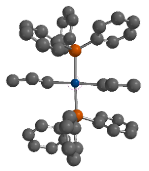

$C_{42}H_{40}P_2Pd$, *EE trans* –2434.27107 au

M06/6-311+G(d,p)·LANL2DZ//…

1 H	H1	4.3964554	1.5022281	0.1716046
2 C	C1	3.6868882	2.3385096	0.0969929
3 H	H2	3.8656328	3.0105571	0.9513696
4 H	H4	3.9364404	2.9093629	-0.8113610
5 C	C2	2.2777257	1.8278349	0.0620925
6 H	H5	1.5020439	2.6021453	-0.0235447
7 C	C3	1.9304470	0.5382768	0.1010094
8 H	H6	2.7611092	-0.1834803	0.1977571
9 P	P1	-0.0309832	-0.0922770	-2.3258682
10 Pd	Pd1	0.0129494	-0.2984119	-0.0007724
11 P	P2	0.1461359	-0.3437779	2.3386391
12 C	C10	-1.9310860	-1.0982992	-0.0334613
13 H	H25	-2.0525381	-2.1374939	0.3221319
14 C	C11	-3.0721343	-0.4507709	-0.2996625
15 H	H26	-3.0428371	0.5937452	-0.6390343
16 C	C12	-4.4502855	-1.0233543	-0.1486507
17 H	H24	-5.0201925	-0.9771317	-1.0899428
18 H	H27	-5.0422213	-0.4701782	0.5987858
19 H	H28	-4.4068296	-2.0742664	0.1712464
20 C	C4	-1.4278147	0.9745423	-2.8213345
21 C	C5	-3.6266473	2.6287492	-3.3103478
22 C	C6	-1.4783248	2.2665479	-2.2833296
23 C	C7	-2.4913436	0.5190979	-3.5997779
24 C	C8	-3.5870820	1.3438971	-3.8396505
25 C	C9	-2.5662884	3.0924979	-2.5344178
26 H	H3	-0.6555076	2.6216419	-1.6592601
27 H	H7	-2.4731728	-0.4912764	-4.0065408
28 H	H8	-4.4161107	0.9764529	-4.4419984
29 H	H9	-2.5935607	4.0966365	-2.1147042
30 H	H10	-4.4856161	3.2702410	-3.4990860
31 C	C13	-0.2265124	1.2957907	3.0540560
32 C	C14	-0.9108924	3.7867559	4.1062284
33 C	C15	-0.1163994	1.5350514	4.4277358
34 C	C16	-0.6991432	2.3069306	2.2166707
35 C	C17	-1.0417494	3.5493042	2.7425426
36 C	C18.	-0.4490618	2.7785575	4.9498990
37 H	H11	0.2292634	0.7402199	5.0904166
38 H	H12	-0.8031608	2.1026509	1.1490309
39 H	H13	-1.4124750	4.3323039	2.0834331
40 H	H14	-0.3556567	2.9602498	6.0191686
41 H	H15	-1.1760011	4.7593984	4.5174146
42 C	C19	1.4302557	0.6572503	-3.1422183
43 C	C20	3.7679818	1.7389752	-4.2294131
44 C	C21	1.3825549	1.8354770	-3.8852275
45 C	C22	2.6619667	0.0185508	-2.9569885
46 C	C23	3.8211490	0.5518465	-3.5001309
47 C	C24	2.5494326	2.3760695	-4.4227464
48 H	H16	0.4315309	2.3402873	-4.0488070
49 H	H17	2.7088654	-0.9004484	-2.3709576
50 H	H18	4.7735306	0.0480265	-3.3448091
51 H	H19	2.4996693	3.2987567	-4.9987161
52 H	H20	4.6797903	2.1633962	-4.6462559
53 C	C25	-0.2338163	-1.6457861	-3.2689677
54 C	C26	-0.5284284	-4.0367617	-4.6775412
55 C	C27	-0.0477049	-1.6757785	-4.6557565
56 C	C28	-0.5639115	-2.8228566	-2.5957540
57 C	C29	-0.7101164	-4.0148568	-3.2995195
58 C	C30	-0.1977847	-2.8658637	-5.3565557
59 H	H21	0.2197736	-0.7609654	-5.1858967
60 H	H22	-0.7060443	-2.7904224	-1.5164920
61 H	H23	-0.9648786	-4.9285924	-2.7655879
62 H	H29	-0.0520282	-2.8818803	-6.4352568
63 H	H30	-0.6403743	-4.9701458	-5.2268276
64 C	C31	-0.9621530	-1.4159206	3.3404456
65 C	C32	-2.7886002	-2.9329490	4.8210227
66 C	C33	-0.5123563	-2.3191758	4.3059697
67 C	C34	-2.3398693	-1.2705800	3.1344597
68 C	C35	-3.2451987	-2.0246636	3.8692838
69 C	C36	-1.4231677	-3.0754856	5.0391591
70 H	H31	0.5544148	-2.4309803	4.4946084
71 H	H32	-2.6995465	-0.5723265	2.3787148
72 H	H34	-4.3130197	-1.9026522	3.6941683
73 H	H35	-1.0584895	-3.7768859	5.7880152
74 H	H36	-3.4978883	-3.5270672	5.3949135
75 C	C37	1.8338021	-0.7848043	2.8785191
76 C	C38	4.5029457	-1.4876741	3.3270848
77 C	C39	2.2817511	-2.0843102	2.6116268
78 C	C40	2.7431666	0.1622679	3.3502955
79 C	C41	4.0709739	-0.1895695	3.5740586
80 C	C42	3.6047634	-2.4367050	2.8456417
81 H	H33	1.5890922	-2.8217087	2.2017426
82 H	H37	2.4210798	1.1888109	3.5190195
83 H	H38	4.7723717	0.5602691	3.9359159
84 H	H39	3.9385056	-3.4521104	2.6388452
85 H	H40	5.5425463	-1.7595165	3.5014350

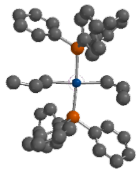

$C_{42}H_{40}P_2Pd$, *ZE trans* –2434.27139 au

M06/6-311+G(d,p)·LANL2DZ//…

1 H	H1	3.2856423	1.4075099	2.8275263
2 C	C1	2.3459039	1.1960436	3.3567487
3 H	H2	2.5445809	0.3953150	4.0877965
4 H	H4	2.0751352	2.0877091	3.9437170
5 C	C2	1.2656029	0.8105474	2.3907388
6 H	H5	0.2966044	0.5709602	2.8500753
7 C	C3	1.4090744	0.7357930	1.0629562
8 H	H6	2.4138101	0.9783271	0.6756952
9 P	P1	1.2508743	-2.0075323	-0.3959448
10 Pd	Pd1	0.0070157	-0.0181924	-0.3133945
11 P	P2	-1.1440730	2.0089813	-0.0875859
12 C	C10	-1.3306815	-0.8832449	-1.6934047
13 H	H25	-0.9192548	-1.0129185	-2.7060526
14 C	C11	-2.5804130	-1.3340001	-1.5318900
15 C	C4	1.2063346	-2.8662440	-2.0112278
16 C	C5	1.1383529	-4.0351079	-4.5504409
17 C	C6	1.4832041	-2.1048795	-3.1531474
18 C	C7	0.8775715	-4.2135094	-2.1579467
19 C	C8	0.8414110	-4.7926965	-3.4238034
20 C	C9	1.4621502	-2.6870851	-4.4138678
21 H	H3	1.7011672	-1.0398335	-3.0466087
22 H	H7	0.6371666	-4.8143741	-1.2819940
23 H	H8	0.5765741	-5.8436366	-3.5266513
24 H	H9	1.6847899	-2.0847399	-5.2928649
25 H	H10	1.1070669	-4.4907678	-5.5385908
26 C	C13	-0.2024387	3.4156493	-0.7866323
27 C	C14	1.2352146	5.5278583	-1.9130914
28 C	C15	-0.7725386	4.6905767	-0.8765231
29 C	C16	1.0889497	3.2090663	-1.2739269
30 C	C17	1.8042907	4.2620265	-1.8374448
31 C	C18	-0.0544003	5.7424972	-1.4304612
32 H	H11	-1.7884786	4.8551280	-0.5150372
33 H	H12	1.5253362	2.2124477	-1.2054020
34 H	H13	2.8090601	4.0899639	-2.2193048
35 H	H14	-0.5046542	6.7315522	-1.4957753
36 H	H15	1.7935997	6.3512323	-2.3554730
37 C	C19	3.0441701	-1.9482521	-0.0028958
38 C	C20	5.7527624	-1.6855161	0.6618723
39 C	C21	4.0319309	-2.0910051	-0.9793548
40 C	C22	3.4318398	-1.6795016	1.3168520
41 C	C23	4.7751892	-1.5503281	1.6446388
42 C	C24	5.3781367	-1.9592398	-0.6479419
43 H	H16	3.7557158	-2.3189164	-2.0077792
44 H	H17	2.6726327	-1.5586358	2.0881173
45 H	H18	5.0562338	-1.3368956	2.6749974
46 H	H19	6.1358916	-2.0795077	-1.4204852
47 H	H20	6.8054615	-1.5811476	0.9192395
48 C	C25	0.6169185	-3.2100503	0.8238328
49 C	C26	-0.4423643	-5.0164528	2.6684336
50 C	C27	1.3450231	-4.3451913	1.1969312
51 C	C28	-0.6363206	-2.9820046	1.3941536
52 C	C29	-1.1663316	-3.8843516	2.3103925
53 C	C30	0.8152058	-5.2448373	2.1137252
54 H	H21	2.3346899	-4.5151156	0.7708586
55 H	H22	-1.1888846	-2.0857787	1.1110477
56 H	H23	-2.1463641	-3.6986054	2.7470196
57 H	H29	1.3877202	-6.1253755	2.4004163
58 H	H30	-0.8541521	-5.7225911	3.3876971
59 C	C31	-2.7775235	2.2426546	-0.8935412
60 C	C32	-5.1948822	2.5260606	-2.2704287
61 C	C33	-3.9110553	2.7289425	-0.2385953
62 C	C34	-2.8652759	1.9119702	-2.2499345
63 C	C35	-4.0640470	2.0574576	-2.9348566
64 C	C36	-5.1155043	2.8650570	-0.9247452
65 H	H31	-3.8601221	3.0005241	0.8150061
66 H	H32	-1.9899712	1.5170275	-2.7642880
67 H	H34	-4.1186150	1.7910382	-3.9889131
68 H	H35	-5.9937125	3.2394834	-0.4013137
69 H	H36	-6.1383996	2.6284800	-2.8040528
70 C	C37	-1.4451500	2.4506903	1.6607888
71 C	C38	-1.7935398	2.9014915	4.3993410
72 C	C39	-2.3043191	1.6403793	2.4115970
73 C	C40	-0.7560355	3.4817620	2.2996547
74 C	C41	-0.9321818	3.7062736	3.6618267
75 C	C42	-2.4831449	1.8680222	3.7705909
76 H	H33	-2.8385884	0.8233077	1.9238076
77 H	H37	-0.0657078	4.1052944	1.7330735
78 H	H38	-0.3866252	4.5124070	4.1496788
79 H	H39	-3.1562543	1.2314830	4.3423669
80 H	H40	-1.9266391	3.0760451	5.4655724
81 C	C43	-3.3959587	-1.2581549	-0.2798179
82 H	H26	-2.8260936	-0.7707921	0.5254619
83 H	H41	-3.7030067	-2.2524501	0.0833501
84 H	H42	-4.3203956	-0.6792003	-0.4380377
85 H	H43	-3.1119067	-1.7763765	-2.3861267

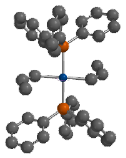

$C_{42}H_{40}P_2Pd$, *ZZ trans* –2434.27295 au

M06/6-311+G(d,p)·LANL2DZ//…

1 C	C2	2.5406468	1.3417555	1.3425537
2 C	C3	1.2982772	0.8528162	1.4408607
3 H	H6	0.8482181	0.9282163	2.4424303
4 P	P1	1.2110423	-2.0133006	-0.1161625
5 Pd	Pd1	0.0119436	0.0001391	-0.0026780
6 P	P2	-1.2008622	2.0078514	0.1198463
7 C	C10	-1.2730603	-0.8527763	-1.4486338
8 H	H25	-0.8120158	-0.9694298	-2.4408366
9 C	C11	-2.5351104	-1.2893013	-1.3564917
10 C	C4	1.3406212	-2.6600287	-1.8242153
11 C	C5	1.6067486	-3.5050238	-4.4770318
12 C	C6	1.8015365	-1.7788809	-2.8093506
13 C	C7	0.9943352	-3.9605130	-2.1875196
14 C	C8	1.1259307	-4.3788645	-3.5096528
15 C	C9	1.9440097	-2.2001928	-4.1248360
16 H	H3	2.0380652	-0.7471348	-2.5407934
17 H	H7	0.6092162	-4.6522834	-1.4398854
18 H	H8	0.8482832	-5.3958435	-3.7814464
19 H	H9	2.3094628	-1.5052433	-4.8787890
20 H	H10	1.7100842	-3.8355633	-5.5089909
21 C	C13	-0.4320116	3.3328385	-0.8796541
22 C	C14	0.7987434	5.3210883	-2.4066031
23 C	C15	-1.0881719	4.5453098	-1.1227433
24 C	C16	0.8337557	3.1208195	-1.4257841
25 C	C17	1.4482376	4.1117190	-2.1853592
26 C	C18	-0.4718276	5.5361504	-1.8769778
27 H	H11	-2.0893596	4.7103806	-0.7226151
28 H	H12	1.3301417	2.1674457	-1.2468328
29 H	H13	2.4373793	3.9345532	-2.6051646
30 H	H14	-0.9867718	6.4781437	-2.0576082
31 H	H15	1.2791438	6.0981267	-2.9988476
32 C	C19	2.9418117	-2.1199931	0.4880346
33 C	C20	5.5341876	-2.2320669	1.5270112
34 C	C21	4.0147426	-2.4788245	0.3294139
35 C	C22	3.1794632	-1.8218662	1.8350999
36 C	C23	4.4665539	-1.8828454	2.3515102
37 C	C24	5.3065586	-2.5310482	0.1889873
38 H	H16	3.8439287	-2.7238533	-1.3768852
39 H	H17	2.3487394	-1.5283661	2.4757669
40 H	H18	4.6393947	-1.6484169	3.4003915
41 H	H19	6.1362294	-2.8116132	-0.4577788
42 H	H20	6.5441198	-2.2743395	1.9316174
43 C	C25	0.4121574	-3.3371549	0.8628859
44 C	C26	-0.8678095	-5.3254808	2.3487289
45 C	C27	1.0589628	-4.5507496	1.1245996
46 C	C28	-0.8704467	-3.1248329	1.3686163
47 C	C29	-1.5090039	-4.1153673	2.1081448
48 C	C30	0.4182397	-5.5416074	1.8583069
49 H	H21	2.0713553	-4.7173313	0.7544871
50 H	H22	-1.3604590	-2.1719858	1.1726716

51	H	H23	-2.5105411	-3.9373872	2.4970390
52	H	H29	0.9261701	-6.4845326	2.0533803
53	H	H30	-1.3671495	-6.1027986	2.9247643
54	C	C31	-2.9421648	2.0933184	-0.4589077
55	C	C32	-5.5528087	2.1442146	-1.4568714
56	C	C33	-4.0118438	2.4132883	0.3788020
57	C	C34	-3.1921358	1.8083272	-1.8063581
58	C	C35	-4.4885187	1.8375451	-2.3022857
59	C	C36	-5.3124175	2.4353157	-0.1191416
60	H	H31	-3.8317554	2.6499419	1.4266848
61	H	H32	-2.3640789	1.5457162	-2.4635217
62	H	H34	-4.6707857	1.6105562	-3.3512336
63	H	H35	-6.1391826	2.6853667	0.5436293
64	H	H36	-6.5701012	2.1601606	-1.8445186
65	C	C37	-1.3083080	2.6613951	1.8267874
66	C	C38	-1.5300089	3.5133227	4.4820172
67	C	C39	-1.7576555	1.7844620	2.8212167
68	C	C40	-0.9497125	3.9605992	2.1825299
69	C	C41	-1.0590033	4.3823992	3.5055462
70	C	C42	-1.8786032	2.2094541	4.1376755
71	H	H33	-2.0017268	0.7527532	2.5592534
72	H	H37	-0.5704262	4.6485666	1.4285448
73	H	H38	-0.7716111	5.3983592	3.7709263
74	H	H39	-2.2355906	1.5177159	4.8986095
75	H	H40	-1.6161059	3.8464509	5.5147268
76	C	C43	-3.4154086	-1.2302701	-0.1478075
77	H	H26	-2.9089688	-0.7155404	0.6819606
78	H	H41	-3.7107372	-2.2327781	0.2025077
79	H	H42	-4.3444513	-0.6769674	-0.3565086
80	H	H4	-3.0257829	-1.7103074	-2.2457791
81	C	C12.	3.3991160	1.3629035	0.1169926
82	H	H5	3.5712549	2.3876415	-0.2518651
83	H	H24	2.9361229	0.7840169	-0.6957366
84	H	H27	4.3936269	0.9310612	0.3083897
85	H	H28	3.0259368	1.7604957	2.2355815

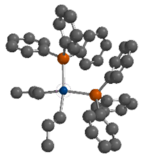

$C_{42}H_{40}P_2Pd$, *EE cis* –2434.27735 au

M06/6-311+G(d,p)·LANL2DZ//...

1	H	H1	0.9399737	-3.0016989	-4.8926862
2	C	C1	1.5655330	-3.3091569	-4.0433908
3	H	H2	1.5462820	-4.4082051	-3.9915978
4	H	H4	2.6038942	-3.0194566	-4.2703902
5	C	C2	1.0876965	-2.6732911	-2.7733980
6	H	H5	1.6783704	-2.9086922	-1.8786180
7	C	C3	0.0589124	-1.8293804	-2.6873112
8	H	H6	-0.5304571	-1.6216081	-3.5917288
9	P	P1	1.4495729	0.5927824	-1.0672391
10	Pd	Pd1	-0.5235154	-0.8468052	-0.9969823
11	P	P2	-1.4551236	-0.2303014	1.1945584
12	C	C10	-2.1242561	-2.1038157	-1.2278820
13	H	H25	-2.3379451	-2.7937109	-0.3962678
14	C	C11	-3.0231169	-2.0524364	-2.2131062
15	H	H2	-2.8530054	-1.3925919	-3.0721455
16	C	C12	-4.3074433	-2.8236379	-2.2286298
17	H	H24	-4.3770288	-3.4968181	-3.0964107
18	H	H27	-5.1807404	-2.1543033	-2.2787602
19	H	H28	-4.4093634	-3.4288425	-1.3170448
20	C	C4	1.1475831	2.3902725	-1.2864425
21	C	C5	0.6211977	5.1251621	-1.5338888
22	C	C6	-0.0985841	2.9084821	-0.9253481
23	C	C7	2.1241473	3.2578479	-1.7895668
24	C	C8	1.8623070	4.6173902	-1.9102755
25	C	C9	-0.3590383	4.2697389	-1.0431118
26	H	H3	-0.8704496	2.2341781	-0.5525701
27	H	H7	3.0929544	2.8622001	-2.0966913
28	H	H8	2.6277255	5.2836351	-2.3048105
29	H	H9	-1.3339775	4.6586337	-0.7552464
30	H	H10	0.4168519	6.1900796	-1.6319238
31	C	C13	-1.2346031	1.4091481	2.0012728
32	C	C14	-0.7358983	3.9899615	2.9777863
33	C	C15	-2.2504531	2.3688355	2.0259176
34	C	C16	0.0330352	1.7598824	2.4805865
35	C	C17	0.2801467	3.0384699	2.9642574
36	C	C18	-2.0018291	3.6490811	2.5138459
37	H	H11	-3.2485518	2.1146474	1.6709246
38	H	H12	0.8333599	1.0204175	2.4847172
39	H	H13	1.2742626	3.2895688	3.3316186
40	H	H14	-2.8073195	4.3815995	2.5335422
41	H	H15	-0.5426407	4.9924196	3.3554178
42	C	C19	2.4865547	0.4887840	0.4445550
43	C	C20	3.9753631	0.1672070	2.7910470
44	C	C21	3.1815479	1.5691935	0.9931378
45	C	C22	2.5550497	-0.7559820	1.0798365
46	C	C23	3.2949598	-0.9183056	2.2459738
47	C	C24	3.9203003	1.4078065	2.1614521
48	H	H16	3.1275622	2.5482347	0.5173633
49	H	H17	2.0013787	-1.5990273	0.6620046
50	H	H18	3.3283285	-1.8904431	2.7349244
51	H	H19	4.4513794	2.2583772	2.5864678
52	H	H20	4.5466098	0.0475137	3.7099829
53	C	C25	2.6576012	0.2536939	-2.4027969
54	C	C26	4.3725095	-0.3227634	-4.5347375
55	C	C27	2.2078606	0.3575827	-3.7244106
56	C	C28	3.9695261	-0.1506014	-2.1621405
57	C	C29	4.8229238	-0.4403263	-3.2261782
58	C	C30	3.0605718	0.0802440	-4.7825233
59	H	H2	1.1752377	0.6497532	-3.9207131
60	H	H22	4.3348443	-0.2388528	-1.1393208
61	H	H23	5.8459136	-0.7540945	-3.0247661
62	H	H29	2.6987770	0.1669304	-5.8056165
63	H	H30	5.0392565	-0.5489343	-5.3651523
64	C	C31	-3.2701920	-0.4754107	1.2851972
65	C	C32	-6.0459892	-0.7955126	1.2815267
66	C	C33	-3.8957788	-1.2086531	2.2932246
67	C	C34	-4.0478085	0.0827878	0.2639303
68	C	C35	-5.4289771	-0.0607779	0.2712440
69	C	C36	-5.2788391	-1.3720542	2.2868515
70	H	H31	-3.3012893	-1.6639062	3.0846656
71	H	H32	-3.5582513	0.6189194	-0.5509203
72	H	H34	-6.0233816	0.3828628	-0.5256729
73	H	H35	-5.7569669	-1.9522837	3.0742564
74	H	H36	-7.1270497	-0.9239005	1.2785619
75	C	C37	-0.7820560	-1.3987496	2.4403602
76	C	C38	0.2565731	-3.2620183	4.2503740
77	C	C39	-0.2626738	-2.6162423	1.9872414
78	C	C40	-0.7792849	-1.1289154	3.8124574
79	C	C41	-0.2595359	-2.0538562	4.7110619
80	C	C42	0.2510212	-3.5438514	2.8878701
81	H	H33	-0.2540770	-2.8269455	0.9150451
82	H	H37	-1.1809594	-0.1843875	4.1799481
83	H	H38	-0.2589427	-1.8296040	5.7764425
84	H	H39	0.6494969	-4.4881761	2.5208026
85	H	H40	0.6624402	-3.9849966	4.9561613

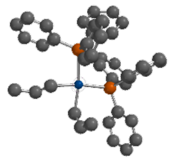

$C_{42}H_{40}P_2Pd$, *ZE cis* –2434.27918 au

M06/6-311+G(d,p)·LANL2DZ//...

1	C	C2	-0.9611402	2.6677987	-3.0601822
2	C	C3	-0.0767073	1.6891524	-2.8733606
3	H	H6	0.3986709	1.2288091	-3.7490007
4	P	P1	-1.4605509	-0.6067113	-1.1497895
5	Pd	Pd1	0.4943296	0.8485079	-1.0977806
6	P	P2	1.4297771	0.2388740	1.1056910
7	C	C10	2.0743897	2.1306547	-1.3541335
8	H	H25	2.2873536	2.8209870	-0.5218532
9	C	C11	2.9609726	2.1094419	-2.3521148
10	H	H26	2.7965085	1.4509214	-3.2127880
11	C	C12	4.2236491	2.9148316	-2.3835880
12	H	H24	4.2713540	3.5750783	-3.2625583
13	H	H27	5.1149518	2.2689833	-2.4254918
14	H	H28	4.3115685	3.5376589	-1.4828556
15	C	C4	-1.1343052	-2.3989427	-1.3706508
16	C	C5	-0.5503081	-5.1213828	-1.6277964
17	C	C6	0.1382962	-2.8845410	-1.0609836
18	C	C7	-2.1085943	-3.2931026	-1.8312460
19	C	C8	-1.8181185	-4.6461046	-1.9567482
20	C	C9	0.4280110	-4.2396699	-1.1823050
21	H	H3	0.9077027	-2.1891786	-0.7236336
22	H	H7	-3.0959345	-2.9212805	-2.1079044
23	H	H8	-2.5814394	-5.3328560	-2.3188600
24	H	H9	1.4224979	-4.6018191	-0.9277460
25	H	H10	-0.3243311	-6.1817397	-1.7278882
26	C	C13	1.2091874	-1.4181934	1.8775750
27	C	C14	0.7354900	-4.0230194	2.8021986
28	C	C15	2.2447709	-2.3557809	1.9260360
29	C	C16	-0.0669166	-1.8032596	2.3083458
30	C	C17	-0.3009919	-3.0936541	2.7657385
31	C	C18	2.0084192	-3.6482497	2.3870968
32	H	H11	3.2480027	-2.0750018	1.6077480
33	H	H12	-0.8883778	-1.0867653	2.2911190
34	H	H13	-1.3025273	-3.3718858	3.0910724
35	H	H14	2.8289048	-4.3632001	2.4239566
36	H	H15	0.5512757	-5.0352402	3.1577980
37	C	C19	-2.5040748	-0.5079309	0.3594436
38	C	C20	-3.9674554	-0.2162139	2.7252823
39	C	C21	-3.2687463	-1.5686011	0.8517774
40	C	C22	-2.4886862	0.7016612	1.0616620
41	C	C23	-3.2160036	0.8494635	2.2381481
42	C	C24	-3.9947311	-1.4223219	2.0293221
43	H	H16	-3.2710787	-2.5253571	0.3299520
44	H	H17	-1.8726413	1.5237672	0.6926596
45	H	H18	-3.1838932	1.7932815	2.7798366
46	H	H19	-4.5800856	-2.2581848	2.4096462
47	H	H20	-4.5289947	-0.1097209	3.6518187
48	C	C25	-2.6569910	-0.2637650	-2.4937020
49	C	C26	-4.3054839	0.4535463	-4.6331259
50	C	C27	-2.2921124	-0.5795062	-3.8071474
51	C	C28	-3.8545367	0.4122289	-2.2646476
52	C	C29	-4.6731109	0.7732631	-3.3319448
53	C	C30	-3.1146845	-0.2312387	-4.8684441
54	H	H21	-1.3497014	-1.0951140	-3.9976004
55	H	H22	-4.1480976	0.6694294	-1.2470873
56	H	H23	-5.6042172	1.3042943	-3.1407864
57	H	H29	-2.8218817	-0.4840124	-5.8860733
58	H	H30	-4.9452804	0.7360842	-5.4674117
59	C	C31	3.2466675	0.4703882	1.1890224
60	C	C32	6.0272280	0.7320568	1.2019355
61	C	C33	3.8895328	1.0976377	2.2561268
62	C	C34	4.0089662	-0.0162496	0.1211041
63	C	C35	5.3930253	0.1010185	0.1345619
64	C	C36	5.2752689	1.2321315	2.2592810
65	H	H31	3.3046267	1.4904963	3.0873001
66	H	H32	3.5055334	-0.4762173	-0.7305036
67	H	H34	5.9768874	-0.2834377	-0.6999821
68	H	H35	5.7679502	1.7317402	3.0917284
69	H	H36	7.1106700	0.8390251	1.2051675
70	C	C37	0.7817420	1.3842903	2.3850630
71	C	C38	-0.2253138	3.2269432	4.2342099
72	C	C39	0.3532669	2.6490997	1.9673318
73	C	C40	0.7084519	1.0578253	3.7423249
74	C	C41	0.2045676	1.9734633	4.6602220
75	C	C42	-0.1457916	3.5657128	2.8865951
76	H	H33	0.4044899	2.9070825	0.9078506
77	H	H37	1.0424145	0.0779021	4.0833088
78	H	H38	0.1497853	1.7058930	5.7141845
79	H	H39	-0.4771707	4.5452065	2.5462648
80	H	H40	-0.6210166	3.9412656	4.9543692
81	C	C43	-1.6688644	3.4315356	-1.9876626
82	H	H5	-2.7600002	3.2864877	-2.0284285
83	H	H41	-1.4914978	4.5142428	-2.0723472
84	H	H42	-1.3173007	3.1121561	-0.9959800
85	H	H43	-1.2174713	2.9571311	-4.0877430

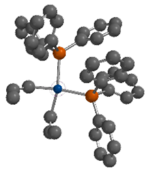

$C_{42}H_{40}P_2Pd$, *ZE cis* –2434.28132 au

M06/6-311+G(d,p)·LANL2DZ//...

1	C	C2	0.9701374	-2.7597206	-3.0214341
2	C	C3	0.0891695	-1.7733361	-2.8547530
3	H	H6	-0.3554088	-1.3058237	-3.7412299
4	P	P1	1.4360995	0.5422504	-1.1065220
5	Pd	Pd1	-0.5035623	-0.9377912	-1.0780664
6	P	P2	-1.4913818	-0.3485637	1.1025468
7	C	C10	-1.9629960	-2.3562312	-1.3947761
8	H	H25	-1.8186848	-3.3061909	-0.8634128
9	C	C11	-3.0392286	-2.2432614	-2.1758036
10	C	C4	1.1164733	2.3351684	-1.3249785
11	C	C5	0.5319061	5.0566844	-1.5895656
12	C	C6	-0.1594424	2.8195223	-1.0269631
13	C	C7	2.0944208	3.2299104	-1.7759422
14	C	C8	1.8035206	4.5826442	-1.9052542

15	C	C9	-0.4493323	4.1744365	-1.1520532
16	H	H3	-0.9301629	2.1239168	-0.6916551
17	H	H7	3.0852935	2.8593042	-2.0410611
18	H	H8	2.5697952	5.2700484	-2.2598911
19	H	H9	-1.4459929	4.5358586	-0.9050329
20	H	H10	0.3057943	6.1166583	-1.6930849
21	C	C13	-1.2788945	1.3118447	1.8761481
22	C	C14	-0.8101697	3.9080484	2.8311337
23	C	C15	-2.3091992	2.2558231	1.9090502
24	C	C16	-0.0103798	1.6875360	2.3366800
25	C	C17	0.2212649	2.9728545	2.8099308
26	C	C18	-2.0755428	3.5435432	2.3851265
27	H	H11	-3.3079286	1.9843383	1.5695078
28	H	H12	0.8055551	0.9648346	2.3337823
29	H	H13	1.2159972	3.2429872	3.1617364
30	H	H14	-2.8929687	4.2625773	2.4098014
31	H	H15	-0.6285609	4.9159444	3.2000780
32	C	C19	2.4632439	0.4330207	0.4131914
33	C	C20	3.8851446	0.1286773	2.8032505
34	C	C21	3.2111707	1.4939051	0.9293480
35	C	C22	2.4459099	-0.7840180	1.1029584
36	C	C23	3.1508346	-0.9377797	2.2919870
37	C	C24	3.9178365	1.3406297	2.1179954
38	H	H16	3.2159951	2.4557443	0.4170267
39	H	H17	1.8492812	-1.6092502	0.7101179
40	H	H18	3.1115491	-1.8860729	2.8255695
41	H	H19	4.4909251	2.1764925	2.5166289
42	H	H20	4.4288147	0.0177918	3.7398487
43	C	C25	2.6425738	0.1952169	-2.4396241
44	C	C26	4.3000938	-0.5385413	-4.5672552
45	C	C27	2.2920517	0.5158473	-3.7558673
46	C	C28	3.8309280	-0.4939097	-2.2020781
47	C	C29	4.6540442	-0.8627349	-3.2633655
48	C	C30	3.1188552	0.1593016	-4.8112993
49	H	H21	1.3569579	1.0419366	-3.9539742
50	H	H22	4.1133970	-0.7554399	-1.1824198
51	H	H23	5.5777434	-1.4041323	-3.0653148
52	H	H29	2.8365059	0.4157651	-5.8309717
53	H	H30	4.9431392	-0.8278800	-5.3966800
54	C	C31	-3.3086700	-0.5692989	1.1674394
55	C	C32	-6.0933021	-0.7985296	1.1561606
56	C	C33	-3.9649583	-1.2263202	2.2078232
57	C	C34	-4.0604306	-0.0361427	0.1159645
58	C	C35	-5.4452713	-0.1383935	0.1151951
59	C	C36	-5.3522808	-1.3442456	2.1983120
60	H	H31	-3.3905379	-1.6568721	3.0274357
61	H	H32	-3.5503582	0.4578088	-0.7128977
62	H	H34	-6.0180298	0.2830816	-0.7097014
63	H	H35	-5.8550479	-1.8653811	3.0113825
64	H	H36	-7.1779204	-0.8924848	1.1504334
65	C	C37	-0.8635142	-1.4821089	2.4032656
66	C	C38	0.1217761	-3.2980737	4.2906989
67	C	C39	-0.4047765	-2.7421379	2.0061510
68	C	C40	-0.8322233	-1.1468853	3.7603012
69	C	C41	-0.3389688	-2.0489884	4.6969776
70	C	C42	0.0828203	-3.6462626	2.9439218
71	H	H33	-0.4280910	-3.0082018	0.9484416
72	H	H37	-1.1906163	-0.1706092	4.0865391
73	H	H38	-0.3163693	-1.7742625	5.7503118
74	H	H39	0.4369278	-4.6229237	2.6185652
75	H	H40	0.5093641	-4.0019343	5.0254109
76	C	C43	1.6430675	-3.5305056	-1.9316429
77	H	H5	2.7331270	-3.3710054	-1.9278312
78	H	H41	1.4836839	-4.6143355	-2.0349413
79	H	H42	1.2513105	-3.2247983	-0.9506671
80	H	H43	1.2547796	-3.0457930	-4.0423213
81	C	C1	-3.3793417	-1.0707782	-3.0401056
82	H	H2	-3.3743790	-1.3405022	-4.1075166
83	H	H1	-2.6506113	-0.2594806	-2.8961083
84	H	H4	-4.3848763	-0.6750747	-2.8242099
85	H	H2	-3.7488679	-3.0789462	-2.2259022

References

S1. Takai, K.; Nitta, K.; Utimoto, K. Simple and Selective Method for Aldehydes (RCHO) → (*E*)-Haloalkenes (RCH:CHX) Conversion by Means of a Haloform–Chromous Chloride System. *J. Am. Chem. Soc.* **1986**, *108*, 7408–7410.

S2. Reaction with DIBALH: (a) Zweifel, G.; Steele, R. B. A New and Convenient Method for the Preparation of Isomerically Pure *α,β*-Unsaturated Derivatives via Hydroalumination of Alkynes. *J. Am. Chem. Soc.* **1967**, *89*, 2754–2755. (b) Alexakis, A.; Duffault, J. M. The Hydroalumination of *ω-tert*-Butoxyalkynes: An Easy Access to *ω*-Hydroxy Alkenyl Iodides; Application to the Synthesis of Dienic Insect Pheromones. *Tetrahedron Lett.* **1988**, *29*, 6243–6246. (c) Jackson, S. K.; Banfield, S. C.; Kerr, M. A. Total Synthesis of (±)-Herbindole B and (±)-cis-Trikentrin B. *Org. Lett.* **2005**, *7*, 1215–1218. Reaction with $ZrCp_2ClH$ (Schwartz' reagent): (d) Huang, Z.; Negishi, E. A Convenient and Genuine Equivalent to $HZrCp_2Cl$ Generated in situ from $ZrCp_2Cl_2$–DIBAL-H. *Org. Lett.* **2006**, *8*, 3675–3678. (e) Lipshutz, B. H.; Keil, R.; Ellsworth, E. L. A New Method for the *in situ* Generation of $Cp_2Z(H)Cl$ (Schwartz' reagent) *Tetrahedron Lett.* **1990**, *31*, 7257–7260.

S3. (a) Boden, C. D. J.; Pattenden, G.; Ye, T. Palladium-Catalyzed Hydrostannylations of 1-Bromoalkynes. A Practical Synthesis of (*E*)-1-Stannylalk-1-enes. *J. Chem. Soc. Perkin Trans. 1* **1996**, 2417–2419. (b) Takai, K.; Ichiguchi, T.; Hikasa, S. A Practical Transformation of Aldehydes into (*E*)-Iodoalkenes with Geminal Dichromium Reagents. *Synlett* **1999**, 1268–1270.

S4. (a) Weber, M.; Singh, F. V.; Vieira, A. S.; Stefani, H. A.; Paixao, M. W. Palladium-Catalyzed Oxidative Homocoupling of Potassium Alkenyltrifluoroborates: Synthesis of Symmetrical 1,3-Dienes. *Tetrahedron Lett.* **2009**, *50*, 4324–4327. (b) Armstrong, R. J.; Garcia-Ruiz, C.; Myers, E. L.; Aggarwal, V. K. Stereodivergent Olefination of Enantioenriched Boronic Esters. *Angew. Chem., Int. Ed.* **2017**, *56*, 786–790.

S5. (a) Moreno, M.; Murruzzu, C.; Riera, A. Enantioselective Synthesis of Sphingadienines and Aromatic Ceramide Analogs. *Org. Lett.* **2011**, *13*, 5184–5187. (b) Pou, A.; Abad, J.-L.; Ordoñez, Y. F.; Garrido, M.; Casas, J.; Fabriàs, G.; Delgado, A. From the Configurational Preference of Dihydroceramide Desaturase-1 towards Δ^6-Unsaturated Substrates to the Discovery of a New Inhibitor. *Chem. Comm.* **2017**, *53*, 4394–4397.

S6. Auge, J.; Boucard, V.; Gil, R.; Lubin-Germain, N.; Picard, J.; Uziel, J. An Alternative Procedure in the Takai Reaction Using Chromium(III) Chloride Hexahydrate as a Convenient Source of Chromium(II). *Synth. Commun.* **2003**, *33*, 3733–3739.

S7. See, for example: (a) Liard, A.; Marek, I. Stereoselective Preparation of *E* Vinyl Zirconium Derivatives from *E* or *Z* Enol Ethers. *J. Org. Chem.* **2000**, *65***,** 7218-7220. (b) Bull, J. A.; Mousseau, J. J.; Charette, A. B. Convenient One-Pot Synthesis of (*E*)-β-Aryl Vinyl Halides from Benzyl Bromides and Dihalomethanes. *Org. Lett.* **2008**, *10*, 5485-5488. (c) Cheung, L. L. W.; Yudin, A. K. Synthesis of Aminocyclobutanes through Ring Expansion of N-Vinyl-β-Lactams. *Org. Lett.* **2009**, *11*, 1281–1284.

S8. See, for example, (a) Barluenga, J.; Moriel, P.; Aznar, F.; Valdés, C. Palladium-Catalyzed Cross-Coupling between Vinyl Halides and *tert*-Butyl Carbazate: First General Synthesis of the Unusual N-Boc-N-alkenylhydrazines. *Org. Lett.* **2007**, *9*, 275278. (b) Wang, Z.; Zhang, G.; Guzei, I.; Verkade, J. G. PhCH:P($MeNCH_2CH_2$)$_3$N: A Novel Ylide for Quantitative *E* Selectivity in the Wittig Reaction. *J. Org. Chem.* **2001**, *66*, 3521–3524. (c) Zhong, Z.; Wang, Z.-Y.; Ni, S.-F.; Dang, L.; Lee, H. K.; Peng, X.-S.; Wong, H. N. C. Ligand-Free Iron-Catalyzed Carbon (sp^2)-Carbon (sp^2) Oxidative Homo-Coupling of Alkenyllithium. *Org. Lett.* **2019**, *21*, 700–704.

S9. (a) Barluenga, J.; Alvarez-García, L. J.; González, J. M. IPy_2BF_4 is also a Useful Reagent for Stereospecific Iodine-Silicon Exchange in Open Chain Trimethylsilylalkenes. *Tetrahedron Lett.* **1995**, *36*, 2153–2156. (b) Kuang, C.; Senboku, H.; Tokuda, M. Stereoselective Synthesis of (*E*)-*β*-Arylvinyl Halides by Microwave-Induced Hunsdiecker Reaction. *Synlett* **2000**, 1439–1442. (c) Shastin, A. V.; Korotchenko, V. N.; Varseev, G. N.; Nenaidenko, V. G.; Balenkova, E. S. New Synthesis of β-Iodostyrenes. *Russ. J. Org. Chem.* **2003**, *39*, 403–407. (d) Kabalka, G. W.; Mereddy, A. R. Iodination of Organotrifluoroborates. Synthesis of Vinyl and Alkynyl Iodides. *Tetrahedron Lett.* **2004**, *45*, 1417–1419.

S10. See, for example: (a) Takagi, K.; Mimura, H.; Inokawa, S. The in situ-Generated Nickel(0)-Catalyzed Homo-coupling of Alkenyl Halides with Zinc Powder. A Specific Outcome in Stereochemistry. *Bull. Chem. Soc. Jpn.* **1984**, *57*, 3517–3522. (b) Kamigata, N.; Ozaki, J.; Kobayashi, M. Reaction of Alkenesulfonyl Chlorides with Olefins Catalyzed by a Ruthenium(II) Complex. A Novel Method for Synthesis of (*E,E*)-1,4-Diaryl-1,3-butadienes. *J. Org. Chem.* **1985**, *50*, 5045–5050. (c) Sasaki, K.; Nakao, K.; Kobayashi, Y.; Sakai, M.; Uchino, N.; Sakakibara, Y.; Takagi, K. Nickel(0)-Triphenylphosphine Complex-Catalyzed Homo-coupling of 1-Alkenyl halides with Zinc Powder. *Bull. Chem. Soc. Jpn.* **1993**, *66*, 2446–2448.

S11. (a) Lipshutz, B. H.; Alami, M.; Susfalk, R. B. Stannylated Enynes as Linchpins for Palladium-Catalyzed Couplings with Unactivated Vinyl Iodides. *Synlett* **1993**, 693–695. (b) Hu, D. X.; Clift, M. D.; Lazarski, K. E.; Thomson, R. J. Enantioselective Total Synthesis and Confirmation of the Absolute and Relative Stereochemistry of Streptorubin B. *J. Am. Chem. Soc.* **2011**, *133*, 1799–1804.

S12. (a) Ghosal, S.; Luke, G. P.; Kyler, K. S. Formation of 1,3-Diynes, 1,3-Dienes, and Biphenyls via the Copper(II) Nitrate Mediated Coupling of Organotin Compounds. *J. Org. Chem.* **1987**, *52*, 4296–4298. (b) Brichacek, M.; Batory, L. A.; Njardarson, J. T. Stereoselective Ring Expansion of Vinyl Oxiranes: Mechanistic Insights and Natural Product Total Synthesis. *Angew. Chem., Int. Ed.* **2010**, *49*, 1648–1651. (c) Itoh, T.; Emoto, S.; Kondo, M.; Ohara, H.; Tanaka, H.; Torii, S. Synthesis of Optically Active 1,3-Dienes from Alkenylstannanes by a Combination of Electrooxidation and Copper-Mediated Homocoupling Reaction. *Electrochim. Acta* **1997**, *42*, 2133–2142.

S13. See Refs 10–12 and 15 of the main text.

S14. Rummel, L.; Schreiner, P. R. Advances and Prospects in Understanding London Dispersion Interactions in Molecular Chemistry. *Angew. Chem., Int. Ed.* **2024**, *63*, e202316364, and references cited therein.
